# Synthesis of Acyclic Quaternary α‑CF_3_‑β-Oxo Carbonyls via Nucleophilic Substitution Induced by Single-Electron Transfer

**DOI:** 10.1021/acs.joc.5c01559

**Published:** 2025-11-06

**Authors:** Xiangyu Tan, Pau Sarró, Elies Molins, Roser Pleixats, Carolina Gimbert-Suriñach, Adelina Vallribera, Albert Granados

**Affiliations:** † Department of Chemistry and Centro de Innovación en Química Avanzada (ORFEO−CINQA), 16719 Universitat Autónoma de Barcelona, 08193 Cerdanyola, Spain; ‡ 54449 Institut de Ciència de Materials de Barcelona (ICMAB-CSIC), Campus UAB, 08193 Bellaterra, Spain

## Abstract

The development of efficient methods for the formation of quaternary centers in organic compounds has been a constant challenge in synthetic chemistry. Quaternary β-oxo esters are fundamental structures in the synthesis of natural products, pharmaceutical compounds, and other bioactive molecules. In this work, a direct and versatile approach to quaternary noncyclic β-oxo esters is reported using trifluoromethyl thianthrenium salt as highly reactive and selective reagent under mild conditions. Mechanistic investigations supported the operation via single-electron transfer-induced nucleophilic substitution.

## Graphical abstract

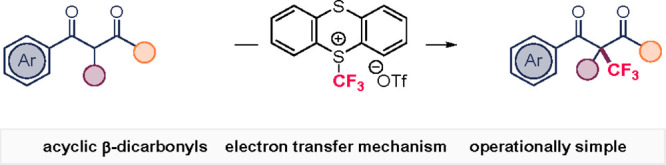

## 1 Introduction

The trifluoromethyl moiety (CF_3_) is a pivotal functional group in modern chemistry due to its deep influence on the physicochemical and biological properties of molecules.^1^ Its high electronegativity and lipophilicity make it invaluable for enhancing metabolic stability, bioavailability, and target affinity in pharmaceuticals or agrochemicals.^2^ In particular, the incorporation of CF_3_ groups on quaternary Csp^3^ centers has garnered significant attention.^3^ Unlike Csp^2^ carbons, quaternary sp^3^ hybridized carbons offer unique three-dimensional structural flexibility, enabling fine-tuning of steric and electronic properties for optimal interactions with biological targets. These features are especially advantageous in drug design, where Csp^3^-enriched compounds often exhibit improved efficacy and reduced off-target effects.^4^ Given their relevance, methods for the selective trifluoromethylation of quaternary Csp^3^ centers are in the cutting research area.

Particularly, quaternary α-substituted α-trifluoromethyl β-keto esters are attractive compounds because they are regarded as nonenolizable β-keto esters.^5^ Since ketones can be readily converted into other functional groups, these compounds serve as versatile synthetic precursors for various α-trifluoromethyl carbonyl derivatives. The introduction of such organofluorinated moieties is typically achieved using electrophilic CF_3_ reagents (Scheme 1).^6^ In 1990, Umemoto^7^ reported efficient trifluoromethyl dibenzoheterocyclic salts for the trifluoromethylation of cyclic β-oxoesters. Subsequently, in 2003, Cahard^8^ utilized 5-(trifluoromethyl)­dibenzothiophenium tetrafluoroborate (Umemoto’s reagent) in the presence of ^*n*^Bu_4_NI as a phase-transfer catalyst to synthesize α-substituted α-trifluoromethyl β-keto esters. Later, Shibata^9^ and Umemoto^10^ designed new trifluoromethyl sulfonium reagents and demonstrated their applicability to β-dicarbonyl compounds. Although these methods proved effective, they often rely on CF_3_ reagents requiring lengthy syntheses, operationally demanding reaction conditions, expensive bases, or exhibit limited functional group tolerance. Although earlier works include selected examples of acyclic substrates, comprehensive methodologies addressing noncyclic 3-oxocarbonyl compounds are still relatively uncommon (Scheme 1).^8–10^

**Scheme 1:**
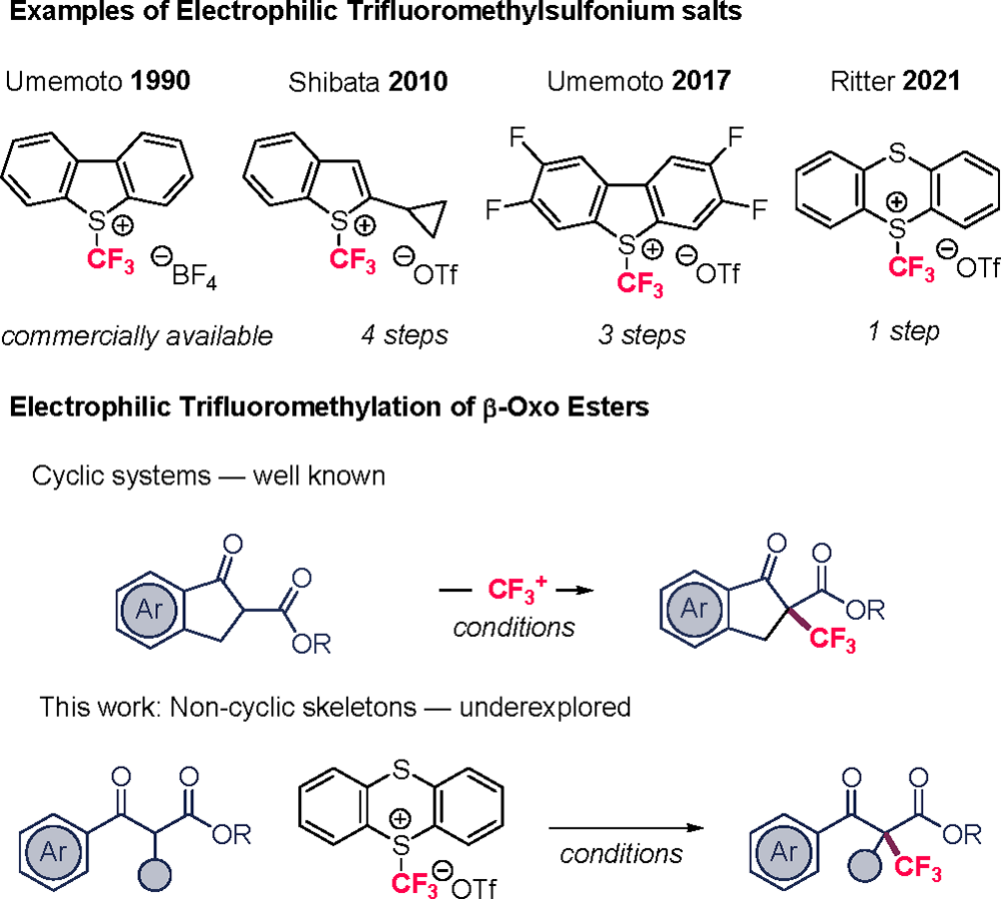
Electrophilic CF_3_-Sulfonium Reagents and Their Use in the Trifluoromethylation of β-Oxo Carbonyls

To address these challenges, we employed trifluoromethyl thianthrenium triflate (**2** in Table 1), an accessible electrophilic CF_3_ reagent from thianthrene in one step reported by Ritter in 2021.^11^ Ritter demonstrated its electrophilic reactivity with a single example involving the trifluoromethylation of a 3-oxo ketone derivative. Additionally, this reagent engaged in radical and nucleophilic trifluoromethylation reactions. Since this pioneering work, different research groups have extensively exploited its use in different radical transformations.^12^ This advance is part of a broader platform involving thianthrenium salts as versatile handles, enabling site-selective functionalization.^13^

**Table 1:**
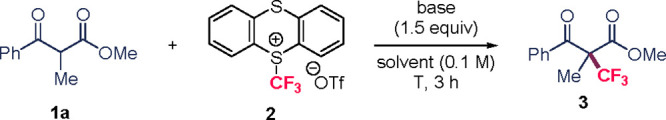
Optimization of the Reaction Conditions^a^

entry	base	solvent	*T* (°C)	isolated yield of **3** (%)
1	NaH	DCM	rt	15
2	NaH	THF	rt	10
3	NaH	MeCN	rt	0
4	NaH	DMSO	rt	8
5	NaH	DMF	rt	0
6	DMAP	DCM	rt	0
7	DBU	DCM	rt	40
8	^*t*^BuOK	DCM	rt	51
9	^*t*^BuOK	DCM	35	69
10^b^	^*t*^BuOK	DCM	35	80

a Reaction conditions: 0.20 mmol of **1a** (1 equiv), 0.24 mmol of **2** (1.2 equiv) and 0.24 mmol of the corresponding base (1.2 equiv) in 0.1 M of the indicated solvent.

b Using 1.5 equiv of **2** in 10 min at 35 °C.

Herein, we expand the range of substrates amenable to electrophilic trifluoromethylation using trifluoromethyl thianthrenium triflate. This work provides an efficient and versatile approach for the synthesis of CF_3_-functionalized quaternary Csp^3^ centers, thereby enriching the synthetic toolbox for biologically and chemically relevant scaffolds (Scheme 1).

## 2 Results and Discussion

Encouraged by the potential synthetic applications of acyclic 3-oxo esters, we investigated the feasibility of the trifluoromethylation reaction using β-keto ester **1a** as model substrate and CF_3_-thianthrenium triflate (**2**) (Table 1). First, we employed reaction conditions similar to those previously reported,^11^ using NaH as base, which afforded the desired compound **3** in 15% yield (Table 1, entry 1). Next, we explored the impact of the solvent, which proved to play a critical role in this process (Table 1, entries 1–5 and Table S1 in the ESI). Notably, dichloromethane (DCM) emerged as essential for the success of this method. We then evaluated the influence of the base on the trifluoromethylation’s efficacy by testing different bases under the optimal solvent. While 4-(dimethylamino)­pyridine (DMAP) failed to promote the formation of compound **3**, amidine 1,8-diazabicyclo(5.4.0)­undec-7-ene (DBU) yielded the desired product in a promising 40% (Table 1, entries 6–7). Remarkably, the use of potassium *tert*-butoxide proved to be the most effective base, achieving up to 80% yield under optimized reaction conditions (1.5 equiv of reagent **2**, 35 °C, 10 min, Table 1 entry 10). Importantly, this method does not require phase-transfer agents or complex bases, proving to be a competent trifluoromethylation protocol.

After establishing the optimal reaction conditions for the trifluoromethylation of acyclic β-keto esters, we evaluated the scope of the reaction using various 3-oxo esters (Table 2). Initially, we investigated the effect of different substituents on the arene ring. Notably, this transformation was amenable to both electron-rich (**4**-**7**) and electron-withdrawing groups (**8**-**9**), with higher yields observed for substrates bearing electron-deficient and moderately activating substituents. The isolated aryl iodide intermediate (**8**) can be subjected to further transformations via cross-coupling methodologies. Additionally, the naphthyl derivative (**10**) was isolated in a good 65% yield. Using an *ortho*-methyl-substituted substrate the product **11** was accessed in a moderate yield of 53%, indicating the reaction is influenced by steric effects. Next, we explored the effect of ester variation. Pleasingly, primary, secondary, and tertiary 3-oxo esters were all successfully isolated (**12**-**15**), yielding the desired quaternary trifluoromethylated products in yields ranging from 56 to 61%. Compound **12** was isolated from 2 mmol in 58% yield, confirming scalability. Of note, the adamantyl derivative (**15**) is not only interesting from the sterics point of view but also presents unique properties from medicinal chemistry to materials science.^14^ Importantly, the yield was independent of the ester chain length or steric bulk. We further replaced the intercarbonyl methyl group with longer hydrocarbon chains (**16**), bulkier isopropyl (**17**), allyl (**18**), and benzylic moieties (**19**), all of which provided the corresponding products with notable efficiency. Remarkably, the reaction exhibited excellent compatibility with terminal alkenes, as exemplified by substrate **18**. Furthermore, we applied the optimized conditions to 3-oxo ketones, which furnished the corresponding products **20** and **21** in 71 and 45% yield, respectively. Notably, the methodology proved amenable to late-stage trifluoromethylation of medicinally and naturally relevant heteroaromatic scaffolds. Representative examples include derivatives of pyridine **(22**), thiophene (**23**), and furan (**24**), all of which delivered the desired products in moderate yields. Finally, alkynyl β-dicarbonyls are also amenable substrates (**25**) for trifluoromethylation. The obtained compounds hold potential for downstream derivatization.^15^

**Table 2:**
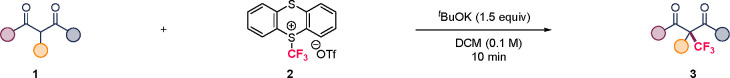

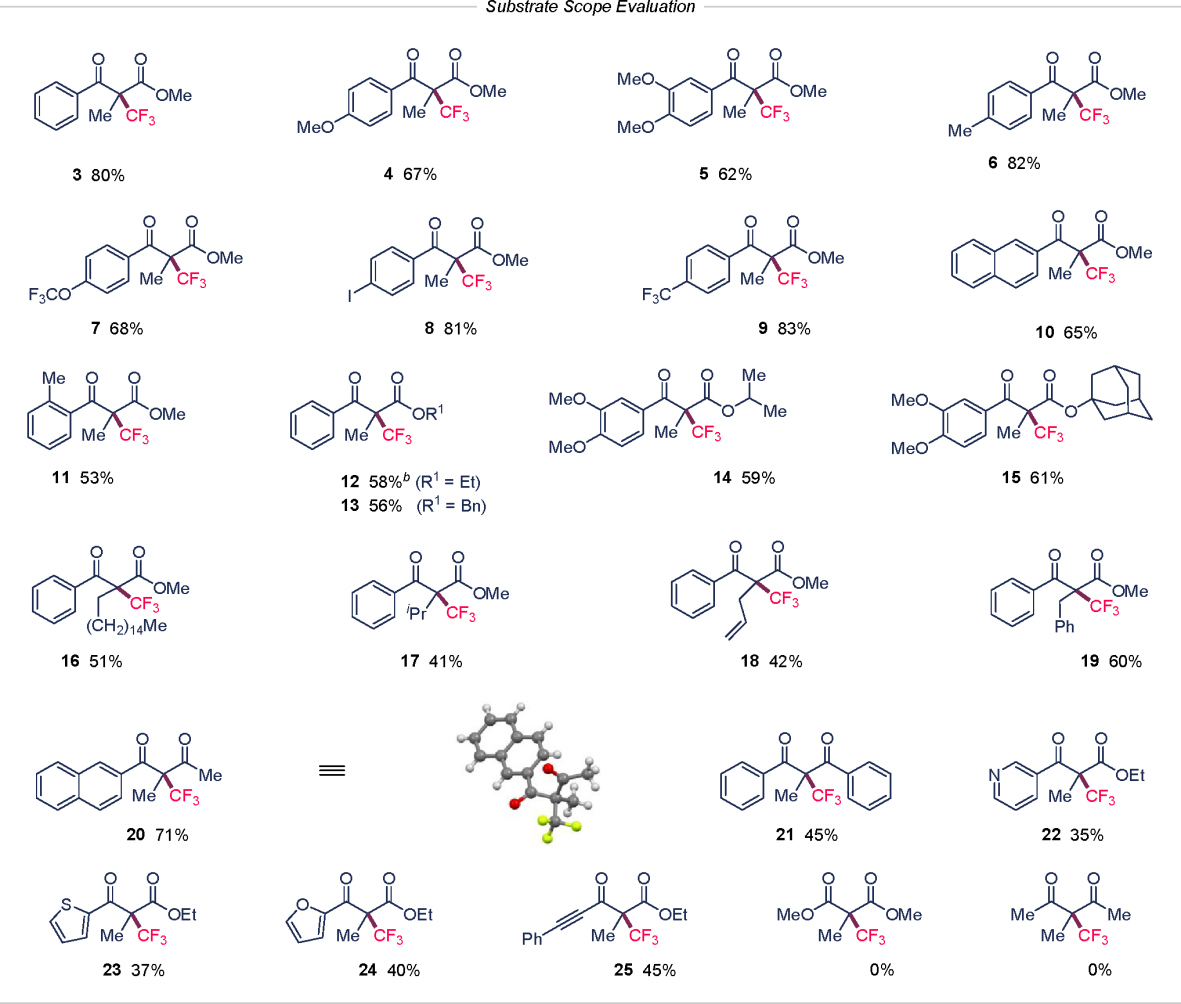
Evaluation of Substrate Scope^a^

a General reaction conditions: **1** (0.5 mmol, 1 equiv), **2** (0.75 mmol, 1.5 equiv), ^*t*^BuOK (0.75 mmol,1.5 equiv) in dry DCM at 35 °C for 10 min. Indicated yield values after purification process.

b From 2 mmol.

Next, we sought to explore the plausible mechanism of this trifluoromethylation reaction through control experiments. The use of reagent **2** as a suitable source of CF_3_ radicals under light irradiation is well-documented in the literature.^12^ To investigate the possible intermediacy of radicals in this reaction, we examined the combination of **1a** with reagent **2** in the presence of Galvinoxyl, a free radical scavenger. Notably, no formation of the desired product was observed, and we were able to isolate the trifluoromethylated Galvinoxyl derivative **26** in 18% yield (Figure 1A). This result strongly suggests that the reaction proceeds via radical species.

**Figure 1:**
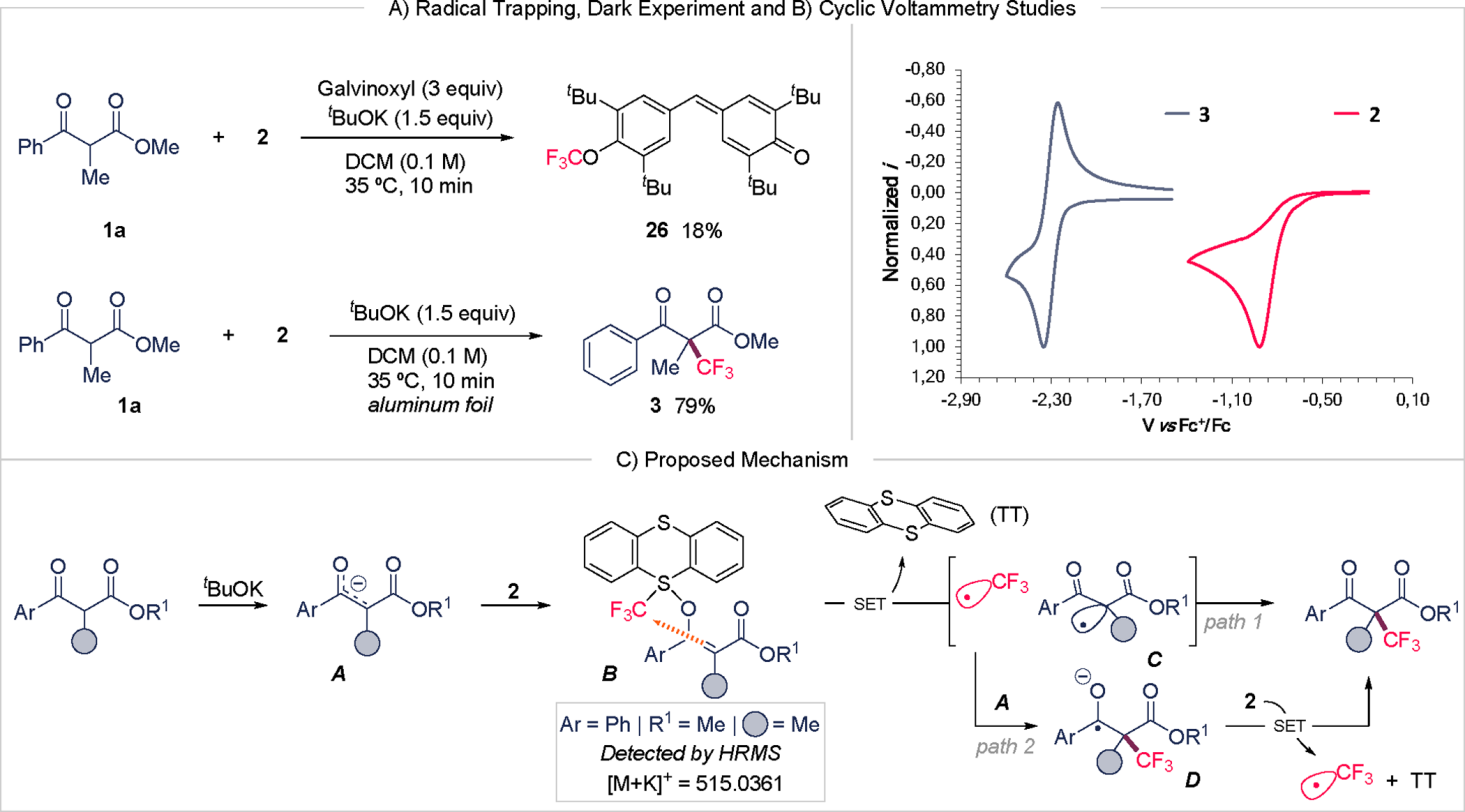
Mechanistic investigations: (A) Radical trapping and dark experiments. (B) Cyclic voltammetry analysis (conditions: **2** [2.3 mM] (100 mV/s), **3** [2.5 mM] (100 mV/s) both in MeCN, TBAPF_6_ 0.1 M at rt. All measurements start at 0.0 V vs reference electrode. Scan direction to negative potentials Glassy carbon disk as working electrode, platinum wire as auxiliary electrode and SCE as reference electrode. IUPAC plotting). (C) Proposed Mechanism. SET = single-electron transfer. TT = thianthrene.

Given that β-dicarbonyl substrates, upon deprotonation with ^*t*^BuOK, generate C-centered nucleophiles, we hypothesized the formation of an electron donor–acceptor (EDA) complex between the enolate anion (electron donor) and reagent **2** (electron acceptor).^16^ To prove this possibility, UV/vis studies were conducted (see Figure S2 in the ESI). Reagent **2** displayed an absorption band in the visible range, whereas β-dicarbonyl **1a** showed no absorption band in this region. When the two were combined, the resulting spectrum resembled that of **1a**, with no significant new absorption bands observed. Remarkably, even upon the addition of ^*t*^BuOK, no spectral changes occurred. These results suggest that a photochemically induced EDA complex is not operative in this reaction since no red shift is manifested. To further corroborate this conclusion, additional experiments were performed. The reaction conducted in the absence of ambient light yielded the desired product without any compromise in yield. Furthermore, lanthanide catalysis, which is well-known to proceed via an enolate mechanism,^3e^ did not afford the desired product (see Table S3 in the ESI), leaving **2** unreacted. The lack of reactivity suggests a sulfurane intermediate formation, which is inhibited when the β-dicarbonyl coordinates to the metal center. To our delight, we could detect the sulfurane intermediate ***B*** of the model reaction via HR-ESI-MS (see Figure 1C and Scheme S1 in the ESI). Overall, these findings suggest that a sulfurane intermediate forms in situ, leading to the final β-oxo esters via open-shell CF_3_ species.

On the basis of our mechanistic investigations and related literature,^8,17^ a plausible mechanism is depicted in Figure 1C. The reaction begins with the generation of the enolate ***A*** intermediate via deprotonation of the β-dicarbonyl substrate using ^*t*^BuOK. The enolate then attacks the cationic sulfur center of reagent **2**, forming an *S*–*O* sulfurane intermediate (***B***).^8^ Subsequently, we propose an intramolecular single-electron transfer (SET) event between the enolate the trifluoromethyl group of the intermediate ***B***. Specifically, this electron transfer process generates thianthrene (TT), species ***C*** and ·CF_3_ radical, which rapidly can undergo geminate recombination to yield the final trifluoromethylated quaternary β-oxo carbonyl product (path 1). Alternatively, we propose a chain propagation mechanism in which the ·CF_3_ radical escapes the solvent cage and couples with nucleophile ***A***, forming the anionic radical intermediate ***D***. This intermediate subsequently produces the desired product through a SET with reagent **2**, regenerating ·CF_3_ radicals and TT (path 2). The complete inhibition of the reaction by Galvinoxyl, along with the thermodynamic feasibility of the SET process between intermediate ***D*** (*E*_*red*_^**3**/**3**·–^ = −2.29 V vs Fc^+^/Fc) and reagent **2** (*E*_*p/2*_^**2**/**2**·^ = −0.81 V vs Fc^+^/Fc) (Figure 1B), suggests that the geminate recombination pathway (path 1) is less likely.^18^ Notably, replacing DCM with DMSO resulted in a lower yield, possibly due to the higher viscosity of DMSO, which may affect radical diffusion and solvent cage dynamics.

## 3 Conclusions

In summary, a trifluoromethylation reaction of acyclic β-dicarbonyl compounds is presented, utilizing readily accessible trifluoromethyl thianthrenium triflate as the key reagent. This method is operationally straightforward and eliminates the need for special bases or additives. Importantly, mechanistic investigations indicate that this trifluoromethylation reaction is the first to be governed by a nucleophilic substitution induced by a SET process using CF_3_-sulfonium reagents. This approach expands the synthetic toolbox for incorporating trifluoromethyl groups.

## 4 Experimental Section

### 4.1 General Information

All chemical transformations requiring inert atmosphere were done using Schlenk line techniques. For violet light irradiation, a Kessil PR160-violet LED lamp (30 W High Luminous DEX 2100 LED, λ_max_ = 427 nm) was placed 4 cm away from the reaction vials. Photoinduced reactions were performed using 4 or 8 mL Chemglass vials (15–425 Green Open Top Cap, TFE Septa). Reactions were monitored by TLC or NMR. TLC analysis was performed using hexanes/EtOAc mixtures as the eluent unless specified and visualized using UV light and/or Vanillin solution. The cyclic voltammetry (CV) experiments were performed with a BioLogic SP-50 Single Channel Potentiostat in a one-compartment three-electrode setup using a glassy carbon disk as the working electrode (o̷ = 3 mm), platinum wire as the auxiliary electrode, and SCE or AgNO_3_/Ag (0.01 M AgNO_3_, 0.1 M [N^*n*^Bu_4_N]­PF_6_ (TBAPF_6_), MeCN) as reference electrodes. CV were performed at room temperature using the appropriate solvent, degassing with argon for 60 s and using TBAPF_6_ as supporting electrolyte (0.1 M). All the experiments were referred to ferrocene as an internal standard. Polishing of the working electrode has been done using an alumina polishing pad with a solution of 0.05 μm alumina in water (purchased from BAS INC.). NMR experiments (^1^H, ^13^C­{^1^H}, ^19^F­{^1^H}) were performed in the *Servei de Ressonància Magnètica Nuclear*, UAB, using NEO 300, 400, NEO 500, or NEO 600 spectrometers. Chemical shifts are referenced to residual, nondeuterated CHCl_3_ (δ 7.26 in ^1^H NMR and 77.16 in ^13^C NMR). The HRMS (ESI+) and elemental analyses were done by the *Servei d’Anàlisi Química* of UAB and *Parque Científico Tecnológico* of UBU. HRMS is determined by a Bruker microTOF-QII mass spectrometer (fly time analyzer) through positive electrospray ionization. IR spectra were recorded on an FT-IR PerkinElmer using either neat oil or solid products. Fluorescence measurements were obtained using septa-capped UV-Quartz cuvettes (10 mm path length) from Hellma Analytics and were recorded in a PerkinElmer LS 55 Fluorescence Spectrometer attached to a PTP 1 Peltier Temperature Programmer maintaining the temperature at 25 °C. Melting points (°C) are uncorrected. Deuterated NMR solvents were purchased from Eurisotop. Dry solvents were obtained from Aldrich or Fisher and used as received. Bulk DCM, EtOAc and hexane were purchased from VWR. Chemicals were purchased from Fluorochem and Merck and used as received unless specified.

### 4.2 General Procedure for the Trifluoromethylation of 1,3-Dicarbonyl Compounds (**3–25**)

To a flame-dried 10 mL vial equipped with a magnetic stirring bar, 1,3-dicarbonyl compound **1** (0.5 mmol, 1 equiv), KO^*t*^Bu (0.60 mmol, 1.2 equiv) and 5 mL of anhydrous DCM were added. The vial was closed with a screwcap and stirred for 5 min. Then, compound **2** (0.75 mmol, 1.5 equiv) was added. The reaction mixture was stirred vigorously at 35 °C using an oil bath. After 10 min, the mixture was diluted with water and extracted with EtOAc (30 mL × 3). The organics were combined, washed with water (15 mL) and brine (15 mL × 2), and finally dried over anhydrous Na_2_SO_4_. After solvent removal under high vacuum, the product was purified by flash column chromatography through silica gel. *Caution!* Standard protective labware should be used, including gloves, lab coat, and safety goggles. Practitioners are advised to take similar precautions when reproducing this work.

### 4.3 Large-Scale Synthesis of Compound **12**

To a flame-dried 50 mL Schlenk flask equipped with a magnetic stirring bar, 1,3-dicarbonyl compound **1j** (2.0 mmol, 1 equiv), KO^*t*^Bu (2.4 mmol, 1.2 equiv) and 20 mL of anhydrous DCM were added. The reaction vessel was closed with a septa and stirred for 5 min. Then, compound **2** (3.0 mmol, 1.5 equiv) was added. The reaction mixture was stirred vigorously at 35 °C using an oil bath. After 10 min, the mixture was diluted with water and extracted with EtOAc (50 mL × 3). The organics were combined, washed with water (15 mL) and brine (15 mL × 2), and finally dried over anhydrous Na_2_SO_4_. After the solvent removal under high vacuum, the product was purified by flash column chromatography through silica gel yielding compound **12** in 58% yield (317.8 mg, 1.16 mmol).

#### 4.3.1 Methyl 2-Benzoyl-3,3,3-trifluoro-2-methylpropanoate (**3**)

Prepared according to the *General Procedure* from the corresponding methyl 2-methyl-3-oxo-3-phenylpropanoate **1a** (96 mg, 0.50 mmol, 1.0 equiv) and TT-CF_3_^+^OTf^–^ **2** (327 mg, 0.75 mmol, 1.5 equiv). After purification by flash column chromatography (hexane: EtOAc 30:1), the title compound **3** was obtained as a pale green oil (104.0 mg, 0.40 mmol, 80%). ^**1**^ **H NMR** (400 MHz, CDCl_3_) δ (ppm) = 7.80–7.73 (m, 2H), 7.56–7.50 (m, 1H), 7.41 (dd, *J* = 8.4, 7.2 Hz, 2H), 3.70 (s, 3H), 1.80 (d, *J* = 1.1 Hz, 3H). ^**13**^ **C­{** ^**1**^ **H} NMR** (151 MHz, CDCl_3_) δ (ppm) = 189.9, 167.9, 134.7, 133.5, 128.7, 128.6, 123.9 (q, *J* = 283.9 Hz), 62.3 (q, *J* = 24.4 Hz), 53.4, 17.6 (q, *J* = 2.7 Hz). ^**19**^ **F­{** ^**1**^ **H} NMR** (376 MHz, CDCl_3_) δ (ppm) = −68.9. **FT-IR** (cm^–1^, neat, ATR), ṽ = 1750, 1696, 1449, 1266, 1222, 1187, 1123, 1094, 972, 843, 795, 690, 628, 572. **HRMS (ESI+)** calcd for C_12_H_11_F_3_O_3_Na [M + Na]^+^: 283.0552, found 283.0552.

#### 4.3.2 Methyl 3,3,3-Trifluoro-2-(4-methoxybenzoyl)-2-methylpropanoate (**4**)

Prepared according to the *General Procedure* from the corresponding methyl 3-(4-methoxyphenyl)-2-methyl-3-oxopropanoate **1b** (111 mg, 0.50 mmol, 1.0 equiv) and TT-CF_3_^+^OTf^–^ **2** (327 mg, 0.75 mmol, 1.5 equiv). After purification by flash column chromatography (hexane: EtOAc 30:1), the title compound **4** was obtained as a pale green oil (95.7 mg, 0.33 mmol, 67%).^**1**^ **H NMR** (300 MHz, CDCl_3_) δ (ppm) = 7.81 (d, *J* = 9.0 Hz, 2H), 6.91 (d, *J* = 9.1 Hz, 2H), 3.85 (s, 3H), 3.73 (s, 3H), 1.81 (s, 3H). ^**13**^ **C­{** ^**1**^ **H} NMR** (151 MHz, CDCl_3_) δ (ppm) = 187.9, 168.3, 163.8, 131.2, 127.2, 124.0 (q, *J* = 283.7 Hz), 113.9, 62.1 (q, *J* = 24.4 Hz), 55.4 (q, *J* = 27.6 Hz), 53.4 (q, *J* = 21.3 Hz), 17.7 (q, *J* = 21.1 Hz). ^**19**^ **F­{** ^**1**^ **H} NMR** (282 MHz, CDCl_3_) δ (ppm) = −69.0. **FT-IR** (cm^–1^, neat, ATR), ṽ = 1747, 1681, 1598, 1573, 1511, 1457, 1259, 1171, 1120, 1091, 1027, 971, 839, 779, 610, 572, 517. **HRMS (ESI+)** calcd for C_13_H_14_F_3_O_4_ [M + H]^+^: 291.0839, found 291.0844.

#### 4.3.3 Methyl 2-(3,4-Dimethoxybenzoyl)-3,3,3-trifluoro-2-methylpropanoate (**5**)

Prepared according to the *General Procedure* from the corresponding methyl 3-(3,4-dimethoxyphenyl)-2-methyl-3-oxopropanoate **1c** (126 mg, 0.50 mmol, 1.0 equiv) and TT-CF_3_^+^OTf^–^ **2** (327 mg, 0.75 mmol, 1.5 equiv). After purification by flash column chromatography (hexane: EtOAc 20:1), the title compound **5** was obtained as a pale green oil (99.2 mg, 0.31 mmol, 62%).^**1**^ **H NMR** (600 MHz, CDCl_3_) δ (ppm) = 7.44 (s, 1H), 7.38 (dd, *J* = 8.5, 2.2 Hz, 1H), 6.83 (d, *J* = 8.6 Hz, 1H), 3.90 (s, 3H), 3.86 (s, 3H), 3.70 (s, 3H), 1.79 (s, 3H). ^**13**^ **C­{** ^**1**^ **H} NMR** (151 MHz, CDCl_3_) δ (ppm) = 187.9, 168.3, 153.7, 149.1, 127.2, 124.0 (q, *J* = 285.4 Hz), 123.0, 111.5, 110.1, 110.0, 62.1 (q, *J* = 24.9 Hz), 56.3–55.5 (m), 53.4 (q, *J* = 21.8 Hz), 17.8 (q, *J* = 28.1 Hz). ^**19**^ **F­{** ^**1**^ **H} NMR** (377 MHz, CDCl_3_) δ (ppm) = −69.1. **FT-IR** (cm^–1^, neat, ATR), ṽ = 1744, 1680, 1593, 1516, 1457, 1417, 1340, 1263, 1209, 1186, 1119, 1019, 878, 817. **HRMS (ESI+)** calcd for C_14_H_16_F_3_O_5_ [M + H]^+^: 321.0944, found 321.0952.

#### 4.3.4 Methyl 3,3,3-Trifluoro-2-methyl-2-(4-methylbenzoyl)­propanoate (**6**)

Prepared according to the *General Procedure* from the corresponding methyl 2-methyl-3-oxo-3-(p-tolyl)­propanoate **1d** (103 mg, 0.50 mmol, 1.0 equiv) and TT-CF_3_^+^OTf^–^ **2** (327 mg, 0.75 mmol, 1.5 equiv). After purification by flash column chromatography (hexane: EtOAc 30:1), the title compound **6** was obtained as a pale green oil (112.3 mg, 0.41 mmol, 82%). ^**1**^ **H NMR** (300 MHz, CDCl_3_) δ (ppm) = 7.74 (d, *J* = 8.4 Hz, 2H), 7.26 (d, *J* = 8.0 Hz, 2H), 3.76 (s, 3H), 2.43 (s, 3H), 1.85 (s, 3H). ^**13**^ **C­{** ^**1**^ **H} NMR** (151 MHz, CDCl_3_) δ (ppm) = 189.3, 168.1, 144.6, 132.0, 129.5, 128.9, 124.0 (q, *J* = 283.9 Hz), 62.3 (q, *J* = 24.7 Hz), 53.8–53.1 (m), 21.6 (q, *J* = 21.3 Hz), 17.7 (q, *J* = 51.4 Hz). ^**19**^ **F­{** ^**1**^ **H} NMR** (282 MHz, CDCl_3_) δ (ppm) = −69.0. **FT-IR** (cm^–1^, neat, ATR), ṽ = 1750, 1687, 1606, 1455, 1268, 1226, 1188, 1121, 1093, 973, 826, 777, 734, 653, 605, 572, 497.**HRMS (ESI+)** calcd for C_13_H_14_F_3_O_3_ [M + H]^+^: 275.0890, found 275.0886.

#### 4.3.5 Methyl 3,3,3-Trifluoro-2-methyl-2-(4-(trifluoromethoxy)­benzoyl)­propanoate (**7**)

Prepared according to the *General Procedure* from the corresponding methyl 2-methyl-3-oxo-3-(4-(trifluoromethoxy)­phenyl)­propanoate **1e** (138 mg, 0.50 mmol, 1.0 equiv) and TT-CF_3_^+^OTf^–^ **2** (327 mg, 0.75 mmol, 1.5 equiv). After purification by flash column chromatography (hexane: EtOAc 20:1), the title compound **7** was obtained as a pale green oil (116.9 mg, 0.34 mmol, 68%). ^**1**^ **H NMR** (600 MHz, CDCl_3_) δ (ppm) = 7.90 (d, *J* = 8.9 Hz, 2H), 7.29 (d, *J* = 9.1 Hz, 2H), 3.78 (s, 3H), 1.85 (s, 3H). ^**13**^ **C­{** ^**1**^ **H} NMR** (151 MHz, CDCl_3_) δ (ppm) = 188.4, 167.8, 152.8, 132.8, 130.8, 123.7 (q, *J* = 283.9 Hz), 120.2, 119.3 (q, *J* = 259.7 Hz), 62.4 (q, *J* = 25.0 Hz), 53.6, 17.5 (q, *J* = 2.6 Hz). ^**19**^ **F­{** ^**1**^ **H} NMR** (377 MHz, CDCl_3_) δ (ppm) = −57.6, −68.8. **FT-IR** (cm^–1^, neat, ATR), ṽ = 1752, 1696, 1603, 1252, 1210, 1164, 1123, 1094, 975, 845, 632, 572. **HRMS (ESI+)** calcd for C_13_H_11_F_6_O_4_ [M + H]^+^: 345.0556, found 345.0560.

#### 4.3.6 Methyl 3,3,3-Trifluoro-2-(4-iodobenzoyl)-2-methylpropanoate (**8**)

Prepared according to the *General Procedure* from the corresponding methyl 3-(4-iodophenyl)-2-methyl-3-oxopropanoate **1f** (159 mg, 0.50 mmol, 1.0 equiv) and TT-CF_3_^+^OTf^–^ **2** (327 mg, 0.75 mmol, 1.5 equiv). After purification by flash column chromatography (hexane: EtOAc 30:1), the title compound **8** was obtained as a pale green oil (154.4 mg, 0.40 mmol, 81%). ^**1**^ **H NMR** (600 MHz, CDCl_3_) δ (ppm) = 7.83 (d, *J* = 8.6 Hz, 2H), 7.53 (d, *J* = 8.6 Hz, 2H), 3.77 (s, 3H), 1.83 (s, 3H). ^**13**^ **C­{** ^**1**^ **H} NMR** (151 MHz, CDCl_3_) δ (ppm) = 189.2, 167.7, 137.9, 133.9, 130.0, 123.8 (q, *J* = 283.9 Hz), 101.9, 62.3 (q, *J* = 24.8 Hz), 54.5–52.7 (m), 17.6 q, *J* = 53.1 Hz. ^**19**^ **F­{** ^**1**^ **H} NMR** (282 MHz, CDCl_3_) δ (ppm) = −68.8. **FT-IR** (cm^–1^, neat, ATR), ṽ = 2924, 1752, 1696, 1579, 1456, 1391, 1267, 1187, 1095, 1007, 962, 890, 825, 706, 635, 572, 492. **HRMS (ESI+)** calcd for C_12_H_11_F_3_IO_3_ [M + H]^+^: 386.9699, found 386.9702.

#### 4.3.7 Methyl 3,3,3-Trifluoro-2-methyl-2-(4-(trifluoromethyl)­benzoyl)­propanoate (**9**)

Prepared according to the *General Procedure* from the corresponding methyl 2-methyl-3-oxo-3-(4-(trifluoromethyl)­phenyl)­propanoate **1g** (130 mg, 0.50 mmol, 1.0 equiv) and TT-CF_3_^+^OTf^–^ **2** (327 mg, 0.75 mmol, 1.5 equiv). After purification by flash column chromatography (hexane: EtOAc 20:1), the title compound **9** was obtained as a pale green oil (134.5 mg, 0.41 mmol, 83%). ^**1**^ **H NMR** (600 MHz, CDCl_3_) δ (ppm) = 7.93 (d, *J* = 8.3 Hz, 2H), 7.74 (d, *J* = 8.3 Hz, 2H), 3.79 (s, 3H), 1.86 (s, 3H). ^**13**^ **C­{** ^**1**^ **H} NMR** (151 MHz, CDCl_3_) δ (ppm) = 189.2, 167.5, 137.7, 134.7 (q, *J* = 33.0 Hz), 129.0, 125.7 (q, *J* = 3.8 Hz), 123.7 (q, *J* = 283.9 Hz), 123.3 (q, *J* = 273.3 Hz), 62.6 (q, *J* = 25.3 Hz), 53.7, 17.5 (q, *J* = 2.5 Hz). ^**19**^ **F­{** ^**1**^ **H} NMR** (282 MHz, CDCl_3_) δ (ppm) = −63.4, −68.7. **FT-IR** (cm^–1^, neat, ATR), ṽ = 1754, 1700, 1326, 1268, 1223, 1169, 1124, 1096, 976, 856, 771, 695, 638. **HRMS (ESI)** calcd for C_13_H_11_F_6_O_3_ [M + H]^+^: 329.0607, found 329.0601.

#### 4.3.8 Methyl 2-(2-Naphthoyl)-3,3,3-trifluoro-2-methylpropanoate (**10**)

Prepared according to the *General Procedure* from the corresponding methyl 2-methyl-3-(naphthalen-2-yl)-3-oxopropanoate **1h** (121 mg, 0.50 mmol, 1.0 equiv) and TT-CF_3_^+^OTf^–^ **2** (327 mg, 0.75 mmol, 1.5 equiv). After purification by flash column chromatography (hexane: EtOAc 20:1), the title compound **3i** was obtained as a pale green oil (99.2 mg, 0.32 mmol, 65%). ^**1**^ **H NMR** (300 MHz, CDCl_3_) δ (ppm) = 8.38 (bs, 1H), 7.98–7.90 (m, 1H), 7.92–7.85 (m, 3H), 7.69–7.56 (m, 2H), 3.77 (s, 3H), 1.94 (bs, 3H). ^**13**^ **C­{** ^**1**^ **H} NMR** (151 MHz, CDCl_3_) δ (ppm) = 189.7, 168.1, 135.5, 132.2, 131.9, 130.6, 129.9, 129.2, 128.6, 127.7, 127.2, 124.1, 123.9 (q, *J* = 286.9 Hz), 62.6 (q, *J* = 24.5 Hz), 53.6 (q, *J* = 26.9 Hz), 17.8 (q, *J* = 23.6 Hz). ^**19**^ **F­{** ^**1**^ **H} NMR** (282 MHz, CDCl_3_) δ (ppm) = −68.8. **FT-IR** (cm^–1^, neat, ATR), ṽ = 1747, 1686, 1626, 1456, 1267, 1195, 1089, 984, 939, 864, 818, 778, 748, 654, 571, 475. **HRMS (ESI+)** calcd for C_16_H_14_F_3_O_3_ [M + H]^+^: 311.0890, found 311.0892.

#### 4.3.9 Methyl 3,3,3-Trifluoro-2-methyl-2-(2-methylbenzoyl)­propanoate (**11**)

Prepared according to the *General Procedure* from the corresponding methyl 2-methyl-3-oxo-3-(o-tolyl)­propanoate **1i** (103 mg, 0.50 mmol, 1.0 equiv) and TT-CF_3_^+^OTf^–^ **2** (327 mg, 0.75 mmol, 1.5 equiv). After purification by flash column chromatography (hexane: EtOAc 30:1), the title compound **3e** was obtained as a pale green oil (71.2 mg, 0.26 mmol, 53%). ^**1**^ **H NMR** (400 MHz, CDCl_3_) δ (ppm) = 7.30 (td, *J* = 7.4, 1.3 Hz, 1H), 7.25–7.20 (m, 2H), 7.17–7.11 (m, 1H), 3.64 (d, *J* = 0.9 Hz, 3H), 2.31 (s, 3H), 1.75 (q, *J* = 0.9 Hz, 3H). ^**13**^ **C­{** ^**1**^ **H} NMR** (126 MHz, CDCl_3_) δ (ppm) = 195.4, 167.2, 137.3, 136.9, 131.7, 130.9, 125.3, 125.1, 124.0 (q, *J* = 284.7 Hz), 64.4 (q, *J* = 24.8 Hz), 53.2, 20.1, 17.6 (q, *J* = 2.6 Hz). ^**19**^ **F­{** ^**1**^ **H} NMR** (377 MHz, CDCl_3_) δ (ppm) = −68.1. **FT-IR** (cm^–1^, neat, ATR), ṽ = 1750, 1702, 1456, 1262, 1184, 1090, 961, 731, 629, 457. **HRMS (ESI+)** calcd for C_13_H_13_F_3_O_3_Na [M + Na]^+^: 297.0709, found 297.0713.

#### 4.3.10 Ethyl 2-Benzoyl-3,3,3-trifluoro-2-methylpropanoate (**12**)

Prepared according to the *General Procedure* from the corresponding ethyl 2-methyl-3-oxo-3-phenylpropanoate **1j** (412 mg, 2.00 mmol, 1.0 equiv) and TT-CF_3_^+^OTf^–^ **2** (1.3 g, 3.00 mmol, 1.5 equiv). After purification by flash column chromatography (hexane: EtOAc 20:1), the title compound **12** was obtained as a pale green oil (317.8 mg, 1.16 mmol, 58%). ^**1**^ **H NMR** (300 MHz, CDCl_3_) δ (ppm) = 7.88–7.78 (m, 2H), 7.62–7.50 (m, 1H), 7.49–7.37 (m, 2H), 4.21 (qd, *J* = 7.1, 2.0 Hz, 2H), 1.83 (bs, 3H), 1.08 (t, *J* = 7.1 Hz, 3H). ^**13**^ **C­{** ^**1**^ **H} NMR** (151 MHz, CDCl_3_) δ (ppm) = 190.1, 167.2, 134.7, 133.5, 128.7, 128.5, 124.0 (q, *J* = 283.9 Hz), 63.0–62.3 (m), 62.8, 17.5 (q, *J* = 2.6 Hz), 13.4. ^**19**^ **F­{** ^**1**^ **H} NMR** (282 MHz, CDCl_3_) δ (ppm) = −69.0. The spectroscopic data matches those described in the literature.^9^

#### 4.3.11 Benzyl 2-Benzoyl-3,3,3-trifluoro-2-methylpropanoate (**13**)

Prepared according to the *General Procedure* from the corresponding benzyl 2-methyl-3-oxo-3-phenylpropanoate **1k** (134 mg, 0.50 mmol, 1.0 equiv) and TT-CF_3_^+^OTf^–^ **2** (327 mg, 0.75 mmol, 1.5 equiv). After purification by flash column chromatography (hexane: EtOAc 30:1), the title compound **13** was obtained as a pale green oil (94.1 mg, 0.28 mmol, 56%). ^**1**^ **H NMR** (300 MHz, CDCl_3_) δ (ppm) = 7.80–7.70 (m, 2H), 7.59–7.46 (m, 1H), 7.40–7.27 (m, 5H), 7.19–7.10 (m, 2H), 5.25–5.16 (m, 2H), 1.87 (s, 3H). ^**13**^ **C­{** ^**1**^ **H} NMR** (126 MHz, CDCl_3_) δ (ppm) = 189.9, 167.2, 134.5, 133.9, 133.4, 128.8, 128.6 (4C), 123.9 (q, *J* = 284.7 Hz), 68.5, 62.6 (q, *J* = 24.7 Hz), 17.7 (q, *J* = 2.5 Hz). ^**19**^ **F­{** ^**1**^ **H} NMR** (282 MHz, CDCl_3_) δ (ppm) = −68.7. **FT-IR** (cm^–1^, neat, ATR), ṽ = 1748, 1693, 1455, 1264, 1220, 1185, 1089, 971, 791, 747, 691, 629, 467. **HRMS (ESI)** calcd for C_18_H_16_F_3_O_3_ [M + H]^+^: 337.1046, found 337.1051.

#### 4.3.12 Isopropyl 2-(3,4-Dimethoxybenzoyl)-3,3,3-trifluoro-2-methylpropanoate (**14**)

Prepared according to the *General Procedure* from the corresponding isopropyl 3-(3,4-dimethoxyphenyl)-2-methyl-3-oxopropanoate **1l** (140 mg, 0.50 mmol, 1.0 equiv) and TT-CF_3_^+^OTf^–^ **2** (327 mg, 0.75 mmol, 1.5 equiv). After purification by flash column chromatography (hexane: EtOAc 20:1), the title compound **14** was obtained as a pale green oil (83.5 mg, 0.24 mmol, 59%). ^**1**^ **H NMR** (300 MHz, CDCl_3_) δ (ppm) = 7.62–7.44 (m, 2H), 6.86 (d, *J* = 8.5 Hz, 1H), 5.12 (hept, *J* = 6.3 Hz, 1H), 3.95 (s, 3H), 3.94 (s, 3H), 1.84 (d, *J* = 0.9 Hz, 3H), 1.15 (d, *J* = 6.3 Hz, 3H), 1.09 (d, *J* = 6.3 Hz, 3H). ^**13**^ **C­{** ^**1**^ **H} NMR** (151 MHz, CDCl_3_) δ (ppm) = 188.5, 167.2, 153.6, 148.9, 127.4, 124.1 (q, *J* = 283.9 Hz), 123.3, 111.6, 109.8, 70.8, 62.5 (q, *J* = 24.2 Hz), 56.1, 55.9, 29.7, 21.1, 17.8 (q, *J* = 2.7 Hz). ^**19**^ **F­{** ^**1**^ **H} NMR** (282 MHz, CDCl_3_) δ (ppm) = −69.0. **FT-IR** (cm^–1^, neat, ATR), ṽ = 1740, 1681, 1594, 1516, 1455, 1417, 1264, 1210, 1153, 1087, 1020, 878, 762, 633, 589. **HRMS (ESI)** calcd for C_16_H_19_F_3_O_5_Na [M + Na]^+^: 371.1077, found 371.1086.

#### 4.3.13 Adamantan-1-yl 2-(3,4-Dimethoxybenzoyl)-3,3,3-trifluoro-2-methylpropanoate (**15**)

Prepared according to the *General Procedure* from the corresponding adamantan-1-yl 3-(3,4-dimethoxyphenyl)-2-methyl-3-oxopropanoate **1m** (220 mg, 0.50 mmol, 1.0 equiv) and TT-CF_3_^+^OTf^–^ **2** (327 mg, 0.75 mmol, 1.5 equiv). After purification by flash column chromatography (hexane: EtOAc 20:1), the title compound **15** was obtained as a pale green oil (152.4 mg, 0.30 mmol, 61%). ^**1**^ **H NMR** (600 MHz, CDCl_3_) δ (ppm) = 7.56–7.51 (m, 2H), 6.87 (d, *J* = 8.4 Hz, 1H), 3.97 (s, 3H), 3.93 (s, 3H), 2.16–2.11 (m, 3H), 2.00 (s, 6H), 1.79 (s, 3H), 1.61 (t, *J* = 3.2 Hz, 6H). ^**13**^ **C­{** ^**1**^ **H} NMR** (151 MHz, CDCl_3_) δ (ppm) = 188.7, 166.1, 153.5, 148.8, 127.3, 124.2 (q, *J* = 283.9 Hz), 123.6, 111.6, 109.8, 109.7, 84.2, 63.2 (q, *J* = 24.6 Hz), 56.2, 56.1, 56.0, 55.8, 40.7, 40.6, 35.9, 35.8, 35.7, 30.7, 17.9 (q, *J* = 2.7 Hz). ^**19**^ **F­{** ^**1**^ **H} NMR** (282 MHz, CDCl_3_) δ (ppm) = −68.9. **FT-IR** (cm^–1^, neat, ATR), ṽ = 2912, 1737, 1681, 1594, 1515, 1456, 1417, 1263, 1152, 1090, 1044, 1020, 963, 869, 813, 764, 731, 632. **HRMS (ESI)** calcd for C_23_H_28_F_3_O_5_ [M + H]^+^: 441.1883, found 441.1874.

#### 4.3.14 Methyl 2-Benzoyl-2-(trifluoromethyl)­octadecanoate (16)

Prepared according to the *General Procedure* from the corresponding methyl 2-benzoyloctadecanoate **1n** (201 mg, 0.50 mmol, 1.0 equiv) and TT-CF_3_^+^OTf^–^ **2** (327 mg, 0.75 mmol, 1.5 equiv). After purification by flash column chromatography (hexane: EtOAc 30:1), the title compound **16** was obtained as a white solid (117.5 mg, 0.25 mmol, 51%). **Mp**: 32–33 °C. ^**1**^ **H NMR** (400 MHz, CDCl_3_) δ (ppm) = 7.74 (d, *J* = 7.8 Hz, 2H), 7.52 (t, *J* = 7.5 Hz, 1H), 7.39 (t, *J* = 7.8 Hz, 2H), 3.67 (d, *J* = 1.5 Hz, 3H), 2.31–2.23 (m, 2H), 1.24–1.16 (bs, 28H), 0.84 (t, *J* = 6.7 Hz, 3H). ^**13**^ **C­{** ^**1**^ **H} NMR** (151 MHz, CDCl_3_) δ (ppm) = 190.4, 167.6, 135.8, 133.3, 128.7, 128.4, 124.1 (q, *J* = 285.4 Hz), 66.2 (q, *J* = 23.7 Hz), 53.1 (q, *J* = 24.2 Hz), 29.6 (13C), 24.3 (q, *J* = 24.2 Hz), 22.7, 14.1. ^**19**^ **F­{** ^**1**^ **H} NMR** (377 MHz, CDCl_3_) δ (ppm) = −65.5. **FT-IR** (cm^–1^, neat, ATR), ṽ = 2916, 2850, 1747, 1691, 1248, 1213, 1143, 1127, 1101, 693. **HRMS (ESI)** calcd for C_27_H_42_F_3_O_3_ [M + H]^+^: 471.3081, found 471.3081.

#### 4.3.15 Methyl 2-Benzoyl-3-methyl-2-(trifluoromethyl)­butanoate (**17**)

Prepared according to the *General Procedure* from the corresponding methyl 2-benzoyl-3-methylbutanoate **1o** (110 mg, 0.50 mmol, 1.0 equiv) and TT-CF_3_^+^OTf^–^ **2** (327 mg, 0.75 mmol, 1.5 equiv). After purification by flash column chromatography (hexane: EtOAc 30:1), the title compound **17** was obtained as a pale green oil (57.6 mg, 0.20 mmol, 41%). ^**1**^ **H NMR** (600 MHz, CDCl_3_) δ (ppm) = 7.80–7.75 (m, 2H), 7.60–7.54 (m, 1H), 7.47–7.43 (m, 2H), 3.71 (s, 3H), 3.01–2.99 (m, 1H), 1.28 (dq, *J* = 6.8, 1.4 Hz, 3H), 1.14 (dt, *J* = 7.0, 1.3 Hz, 3H). ^**13**^ **C­{** ^**1**^ **H} NMR** (151 MHz, CDCl_3_) δ (ppm) = 191.2, 167.3, 136.7, 133.1, 128.6, 128.2, (q, *J* = 286.9 Hz), 70.2–69.6 (m), 52.8 (q, *J* = 26.1 Hz), 32.6, 32.5, 19.5, 19.3 (q, *J* = 17.1 Hz). ^**19**^ **F­{** ^**1**^ **H} NMR** (282 MHz, CDCl_3_) δ (ppm) = −60.7. **IR** (cm^–1^, neat, ATR), ṽ = 2963, 1735, 1684, 1597, 1447, 1283, 1214, 1154, 1119, 1084, 1001, 943, 833, 788, 741, 689, 657, 616. **HRMS (ESI)** calcd for C_14_H_16_F_3_O_3_ [M + H]^+^: 289.1046, found 289.1040.

#### 4.3.16 Methyl 2-Benzoyl-2-(trifluoromethyl)­pent-4-enoate (**18**)

Prepared according to the *General Procedure* from the corresponding methyl 2-benzoylpent-4-enoate **1p** (109 mg, 0.50 mmol, 1.0 equiv) and TT-CF_3_^+^OTf^–^ **2** (327 mg, 0.75 mmol, 1.5 equiv). After purification by flash column chromatography (hexane: EtOAc 30:1), the title compound **18** was obtained as a pale green oil (60.1 mg, 0.21 mmol, 42%). ^**1**^ **H NMR** (600 MHz, CDCl_3_) δ (ppm) = 7.85–7.80 (m, 2H), 7.63–7.56 (m, 1H), 7.46 (dd, *J* = 8.4, 7.5 Hz, 2H), 5.82 (dddd, *J* = 16.9, 7.4, 2.7, 1.3 Hz, 1H), 5.13–5.05 (m, 2H), 3.73 (s, 3H), 3.16–3.08 (m, 2H). ^**13**^ **C­{** ^**1**^ **H} NMR** (151 MHz, CDCl_3_) δ (ppm) = 189.6, 167.0, 135.6, 133.5, 131.0, 128.7, 128.6, 123.7 (q, *J* = 285.4 Hz), 120.3, 66.3 (m), 53.2 (m), 36.5 (m). ^**19**^ **F­{** ^**1**^ **H} NMR** (377 MHz, CDCl_3_) δ (ppm) = −65.4. **FT-IR** (cm^–1^, neat, ATR), ṽ = 1750, 1692, 1446, 1299, 1259, 1211, 1171, 1121, 1030, 927, 803, 752, 691, 658, 621. **HRMS (ESI)** calcd for C_14_H_14_F_3_O_3_ [M + H]^+^: 287.0890, found 287.0893.

#### 4.3.17 Methyl 2-Benzoyl-2-benzyl-3,3,3-trifluoropropanoate (**19**)

Prepared according to the *General Procedure* from the corresponding thianthrenium salt **1q** (134 mg, 0.50 mmol, 1.0 equiv) and TT-CF_3_^+^OTf^–^ **2** (327 mg, 0.75 mmol, 1.5 equiv). After purification by flash column chromatography (hexane: EtOAc 30:1), the title compound **19** was obtained as a pale green oil (100.8 mg, 0.30 mmol, 60%). ^**1**^ **H NMR** (300 MHz, CDCl_3_) δ (ppm) = 7.82–7.71 (m, 2H), 7.65–7.48 (m, 1H), 7.48–7.36 (m, 2H), 7.29–7.17 (m, 5H), 3.82–3.68 (m, 2H), 3.60 (d, *J* = 0.5 Hz, 3H). ^**13**^ **C­{** ^**1**^ **H} NMR** (151 MHz, CDCl_3_) δ (ppm) = 190.0, 167.1, 136.1, 134.1, 133.3, 130.7, 128.8, 128.4, 127.5, 127.4, 123.5 (q, *J* = 285.4 Hz), 67.6 (q, *J* = 23.1 Hz), 52.8 (q, *J* = 9.8 Hz), 37.4 (q, *J* = 25.7 Hz). ^**19**^ **F­{** ^**1**^ **H} NMR** (282 MHz, CDCl_3_) δ (ppm) = −64.3. **FT-IR** (cm^–1^, neat, ATR), ṽ = 1749, 1686, 1447, 1254, 1163, 1081, 1032, 797, 756, 738, 692, 600, 529. **HRMS (ESI)** calcd for C_18_H_15_F_3_O_3_Na [M + Na]^+^: 359.0866, found 359.0870.

#### 4.3.18 2-Methyl-1-(naphthalen-2-yl)-2-(trifluoromethyl)­butane-1,3-dione (**20**)

Prepared according to the *General Procedure* from the corresponding 2-methyl-1-(naphthalen-2-yl)­butane-1,3-dione **1r** (113 mg, 0.50 mmol, 1.0 equiv) and TT-CF_3_^+^OTf^–^ **2a** (327 mg, 0.75 mmol, 1.5 equiv). After purification by flash column chromatography (hexane: EtOAc 20:1), the title compound **20** was obtained as a pale green solid (102.9 mg, 0.35 mmol, 71%). **Mp**: 63–64 °C. ^**1**^ **H NMR** (400 MHz, CDCl_3_) δ (ppm) = 8.24 (d, *J* = 1.8 Hz, 1H), 7.96–7.78 (m, 4H), 7.61–7.51 (m, 2H), 2.27 (d, *J* = 1.0 Hz, 3H), 1.81 (d, *J* = 1.1 Hz, 3H). ^**13**^ **C­{** ^**1**^ **H} NMR** (151 MHz, CDCl_3_) δ (ppm) = 200.2, 191.6, 135.6, 132.2, 132.0, 131.1, 129.9, 129.3, 128.8, 127.7, 127.3, 125.2, 124.3 (q, *J* = 286.9 Hz), 69.2, 28.8, 16.8. ^**19**^ **F­{** ^**1**^ **H} NMR** (377 MHz, CDCl_3_) δ (ppm) = −67.1. **FT-IR** (cm^–1^, neat, ATR), ṽ = 1714, 1686, 1267, 1223, 1158, 1134, 1090, 997, 920, 821, 754, 691, 581, 525, 480. **HRMS (ESI)** calcd for C_16_H_13_F_3_O_2_Na [M + Na]^+^: 317.0760, found 317.0766.

#### 4.3.19 2-Methyl-1,3-diphenyl-2-(trifluoromethyl)­propane-1,3-dione (**21**)

Prepared according to the *General Procedure* from the corresponding 2-methyl-1,3-diphenylpropane-1,3-dione **1s** (119 mg, 0.50 mmol, 1.0 equiv) and TT-CF_3_^+^OTf^–^ **2** (327 mg, 0.75 mmol, 1.5 equiv). After purification by flash column chromatography (hexane: EtOAc 20:1), the title compound **21** was obtained as a pale green oil (70.4 mg, 0.23 mmol, 45%). ^**1**^ **H NMR** (600 MHz, CDCl_3_) δ (ppm) = 7.90–7.81 (m, 4H), 7.49 (td, *J* = 7.3, 1.4 Hz, 2H), 7.40–7.32 (m, 4H), 1.97 (d, *J* = 1.3 Hz, 3H). ^**13**^ **C­{** ^**1**^ **H} NMR** (151 MHz, CDCl_3_) δ (ppm) = 192.4, 135.1, 133.8, 129.6, 128.7, 124.4­(q, *J* = 285.4 Hz), 67.9 (q, *J* = 22.8 Hz), 18.9 (q, *J* = 2.6 Hz). ^**19**^ **F­{** ^**1**^ **H} NMR** (377 MHz, CDCl_3_) δ (ppm) = −66.8. **FT-IR** (cm^–1^, neat, ATR), ṽ = 1671, 1596, 1447, 1257, 1214, 1174, 1101, 1075, 976, 949, 837, 776, 685, 666, 621, 570, 495. **HRMS (ESI)** calcd for C_17_H_14_F_3_O_2_ [M + H]^+^: 307.0940, found 307.0943.

#### 4.3.20 Ethyl 3,3,3-Trifluoro-2-isonicotinoyl-2-methylpropanoate (**22**)

Prepared according to the *General Procedure*. After purification by flash column chromatography (hexane: EtOAc 20:1), the title compound **22** was obtained as an oil (48.1 mg, 0.18 mmol, 35%). ^**1**^ **H NMR** (300 MHz, CDCl_3_) δ (ppm) 8.80 (d, *J* = 6.2 Hz, 2H), 7.59 (d, *J* = 6.2 Hz, 2H), 4.26 (qd, *J* = 7.1, 2.5 Hz, 2H), 1.84 (s, 3H), 1.16 (t, *J* = 7.1 Hz, 3H). ^**13**^ **C­{** ^**1**^ **H} NMR** (75 MHz, CDCl_3_) δ (ppm) = 190.1, 166.5, 150.8, 141.3, 123.6 (q, *J* = 283.8 Hz), 121.3, 63.2, 62.8 (q, *J* = 25.1 Hz), 17.2 (q, *J* = 2.4 Hz), 13.5. ^**19**^ **F­{** ^**1**^ **H} NMR** (282 MHz, CDCl_3_) δ (ppm) = −68.7. **HRMS (ESI)** calcd for C_12_H_13_F_3_NO_3_ [M + H]^+^: 276.0842, found 276.0842.

#### 4.3.21 Ethyl 3,3,3-Trifluoro-2-methyl-2-(thiophene-2-carbonyl)­propanoate (**23**)

Prepared according to the *General Procedure*. After purification by flash column chromatography (hexane: EtOAc 20:1), the title compound **23** was obtained as an oil (51.8 mg, 0.19 mmol, 37%). ^**1**^ **H NMR** (400 MHz, CDCl_3_) δ (ppm) 7.72 (dd, *J* = 5.0, 1.0 Hz, 1H), 7.64 (d, *J* = 4.0 Hz, 1H), 7.13 (dd, *J* = 5.0, 4.0 Hz, 1H), 4.26 (dd, *J* = 7.1, 3.2 Hz, 2H), 1.85 (s, 3H), 1.17 (t, *J* = 7.1 Hz, 3H). ^**13**^ **C­{** ^**1**^ **H} NMR** (75 MHz, CDCl_3_) δ (ppm) = 182.5, 167.2, 141.3, 135.3, 132.7, 128.4, 123.7 (q, *J* = 283.6 Hz), 62.9, 62.7 (q, *J* = 25.0 Hz), 17.6 (q, *J* = 2.2 Hz), 13.6. ^**19**^ **F­{** ^**1**^ **H} NMR** (282 MHz, CDCl_3_) δ (ppm) = −69.0. **HRMS (ESI)** calcd for C_11_H_12_F_3_SO_3_ [M + H]^+^: 281.0454, found 281.0455.

#### 4.3.22 Ethyl 3,3,3-Trifluoro-2-(furan-2-carbonyl)-2-methylpropanoate (**24**)

Prepared according to the *General Procedure*. After purification by flash column chromatography (hexane: EtOAc 20:1), the title compound **24** was obtained as an oil (52.8 mg, 0.20 mmol, 40%). ^**1**^ **H NMR** (300 MHz, CDCl_3_) δ (ppm) ^1^H NMR (300 MHz, Chloroform-*d*) δ 7.59–7.57 (m, 1H), 7.35–7.34 (m, 1H), 6.59 (bs, 1H), 4.26 (qd, *J* = 7.1, 1.4 Hz, 2H), 1.80 (s, 3H), 1.16 (t, *J* = 7.1 Hz, 3H). ^**13**^ **C­{** ^**1**^ **H} NMR** (75 MHz, CDCl_3_) δ (ppm) = 178.7, 166.2, 150.5, 146.7, 123.8 (q, *J* = 283.7 Hz), 119.7, 112.8, 62.6, 61.7 (q, *J* = 25.0 Hz), 16.2 (d, *J* = 2.5 Hz), 13.7. ^**19**^ **F­{** ^**1**^ **H} NMR** (282 MHz, CDCl_3_) δ (ppm) = −69.4. **HRMS (ESI)** calcd for C_11_H_12_F_3_O_4_ [M + H]^+^: 265.0682, found 265.0682.

#### 4.3.23 Ethyl 2-Methyl-3-oxo-5-phenyl-2-(trifluoromethyl)­pent-4-ynoate (**25**)

Prepared according to the *General Procedure*. After purification by flash column chromatography (hexane: EtOAc 20:1), the title compound **24** was obtained as an yellow oil (67 mg, 0.23 mmol, 45%). ^**1**^ **H NMR** (300 MHz, CDCl_3_) δ (ppm) ^1^H NMR (300 MHz, Chloroform-*d*) δ 7.66–7.37 (m, 5H), 4.35 (m, 2H), 1.80 (d, *J* = 0.9 Hz, 3H), 1.32 (t, *J* = 7.1 Hz, 3H). ^**13**^ **C­{** ^**1**^ **H} NMR** (151 MHz, CDCl_3_) δ (ppm) = 176.6, 165.4, 133.4, 131.6, 128.8, 123.5 (q, *J* = 283.9 Hz), 118.9, 95.6, 85.3, 65.1 (q, *J* = 24.1 Hz), 62.9, 15.9, 13.8. ^**19**^ **F­{** ^**1**^ **H} NMR** (282 MHz, CDCl_3_) δ (ppm) = −68.5. **HRMS (ESI)** calcd for C_15_H_13_F_3_O_3_Na [M + Na]^+^: 321.0709, found 321.0710.

## Supplementary Material



## Data Availability

The data underlying this study are available in the published article and its Supporting Information.

## Notes

Preparation of starting materials, copies of nuclear magnetic resonance (NMR) spectra of new compounds, and mechanistic studies and monocrystal X-ray diffraction data (PDF)
