# Supplementary material for: Synthesis of Acyclic Quaternary α‑CF3‑β-Oxo Carbonyls via Nucleophilic Substitution Induced by Single-Electron Transfer
Source: J Org Chem. 2025 Nov 6;90(45):15956–64. doi: 10.1021/acs.joc.5c01559 (PMC12624823; doi:10.1021/acs.joc.5c01559)
Supplement: Supplementary file 1 [file jo5c01559_si_001.pdf]

Supporting Information

Synthesis of Acyclic Quaternary α-CF_3-β-Oxo Carbonyls via Nucleophilic Substitution Induced by Single-Electron Transfer

Xiangyu Tan,[a] Pau Sarró,[a] Elies Molins,[b] Roser Pleixats,[a] Carolina Gimbert-Suriñach,[a] Adelina Vallribera[a,]* and Albert Granados[a,]*

[a]*Department of Chemistry and Centro de Innovación en Química Avanzada (ORFEO-CINQA), Universitat Autònoma de Barcelona, Cerdanyola del Vallès, 08193 Barcelona, Spain*

[b]*Institut de Ciència de Materials de Barcelona (ICMAB-CSIC), Campus UAB, 08193, Bellaterra, Spain*

*To whom correspondence should be addressed.

E-mail: adelina.vallribera@uab.es albert.granados@uab.es

TABLE OF CONTENT

1. Synthesis of Starting Materials 2

2. Trifluoromethylation of α-Substituted β-Keto Esters and β-Diketones. Optimization Process and Compound Characterization 7

3. Data of X-Ray Structure of Compound 20 9

4. Mechanistic Investigation 11

5. Unsuccessful Substrates 14

6. NMR Spectra 15

6. References 50

1. Synthesis of Starting Materials

Synthesis of α-alkyl-β-keto esters and α-methyl-β-diketones 1 and reagent 2a

Compounds **1a**,[1] **1b**,[1] **1c**,[2] **1d**,[1] **1f**,[4] **1g**,[3] **1h**,[4] **1i**,[1] **1n**,[5] **1o**,[6] **1p**,[7] **1q**[1], **1r**,[10] **1s**,[5] **1u**,[16] **1v**,[16] **1w**[17] were prepared according to a reported method.[2] Reaction of the corresponding commercial methyl ketone derivative with sodium hydride and dimethyl carbonate gave rise to the β-keto carbonyls. Then, α-alkylation was accomplished using the desired alkyl iodide. Compounds **1j**,[4] **1k**,[1] **1l**,[8] and **1m**[2] were prepared from by transesterification reaction.[9]

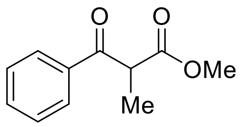

1a

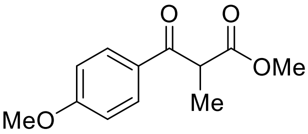

1b

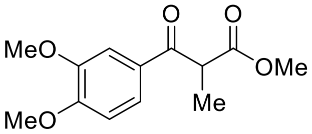

1c

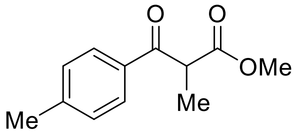

1d

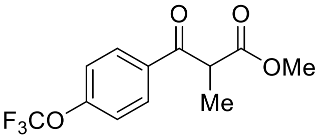

1e

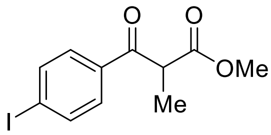

1f

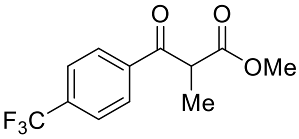

1g

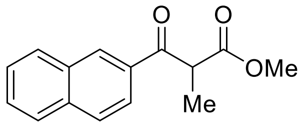

1h

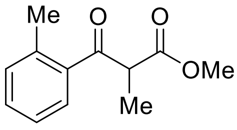

1i

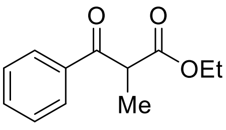

1j

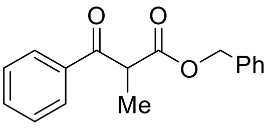

1k

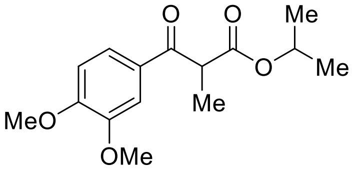

1l

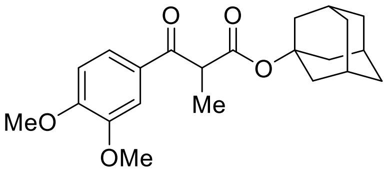

1m

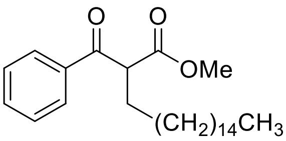

1n

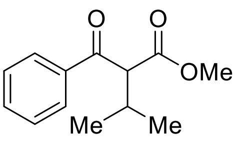

1o

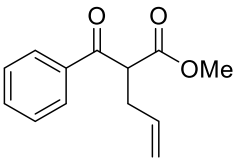

1p

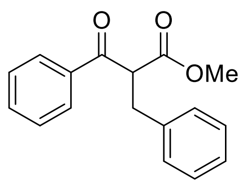

1q

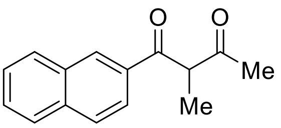

1r

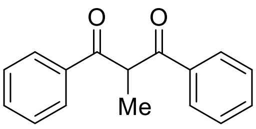

1s

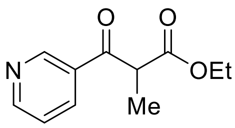

1t

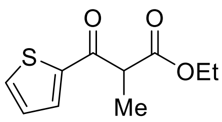

1u

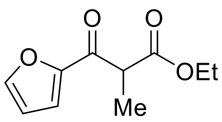

1v

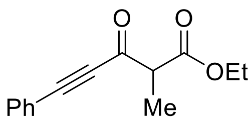

1w

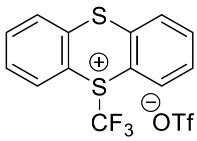

2

Methyl 2-Methyl-3-oxo-3-phenylpropanoate (**1a**): According to the general procedure, 1.61 g of **1a** were synthesized from 10 mmol of starting material (84% yield with two steps) as a pale green oil after purification on silica gel (hexane: ethyl acetate = 9:1). **^{1}H NMR** (400 MHz, Chloroform-*d*) δ 7.97 – 7.90 (m, 2H), 7.59 – 7.50 (m, 1H), 7.48 – 7.39 (m, 2H), 4.37 (q, *J* = 7.1 Hz, 1H), 3.64 (s, 3H), 1.44 (s, 3H). The characterization data is consistent with reported literature.[6]

Methyl 3-(4-Methoxyphenyl)-2-methyl-3-oxopropanoate (**1b**): According to the general procedure, 1.82 g of **1b** were synthesized from 10 mmol of starting material (82% yield with two steps) as a pale green oil after purification on silica gel (hexane: ethyl acetate = 9:1). **^{1}H NMR** (300 MHz, Chloroform-*d*) δ 7.98 – 7.87 (m, 2H), 6.96 – 6.85 (m, 2H), 4.35 (q, *J* = 7.1 Hz, 1H), 3.82 (s, 3H), 3.63 (s, 3H), 1.43 (d, *J* = 7.1 Hz, 3H). The characterization data is consistent with reported literature.[6]

Methyl 3-(3,4-Dimethoxyphenyl)-2-methyl-3-oxopropanoate (**1c**): According to the general procedure, 1.99 g of **1c** were synthesized from 10 mmol of starting material (79% yield with two steps) as a pale green solid after purification on silica gel (hexane: ethyl acetate = 9:1). **^{1}H NMR** (300 MHz, Chloroform-*d*) δ 7.59 (dd, *J* = 8.4, 2.1 Hz, 1H), 7.53 (d, *J* = 2.1 Hz, 1H), 6.88 (d, *J* = 8.4 Hz, 1H), 4.37 (q, *J* = 7.1 Hz, 1H), 3.92 (d, *J* = 6.2 Hz, 6H), 3.66 (d, *J* = 1.3 Hz, 3H), 1.46 (d, *J* = 7.1 Hz, 3H). The characterization data is consistent with reported literature.[1]

Methyl 2-Methyl-3-oxo-3-(p-tolyl)propanoate (**1d**): According to the general procedure, 1.71 g of **1d** were synthesized from 10 mmol of starting material (83% yield with two steps) as a brown oil after purification on silica gel (hexane: ethyl acetate = 9:1). **^{1}H NMR** (300 MHz, Chloroform-*d*) δ 7.92 – 7.82 (m, 2H), 7.31 – 7.21 (m, 2H), 4.39 (q, *J* = 7.1 Hz, 1H), 3.66 (s, 3H), 2.40 (s, 3H), 1.46 (s, 3H). The characterization data is consistent with reported literature.[6]

Methyl 2-Methyl-3-oxo-3-(4-(trifluoromethoxy)phenyl)propanoate (**1e**): **^{1}H NMR** (400 MHz, $CDCl_3$) δ 7.98 (d, *J* = 8.8 Hz, 2H), 7.24 (d, *J* = 9.1 Hz, 2H), 4.32 (q, *J* = 7.1 Hz, 1H), 3.63 (s, 3H), 1.43 (d, *J* = 7.1 Hz, 3H). **^{13}C NMR** (151 MHz, $CDCl_3$) δ 194.2, 170.9, 152.8, 152.8, 133.9, 130.6, 120.4, 120.2 (q, *J* = 258.2 Hz), 52.5, 48.1, 13.6. **^{19}F NMR** (377 MHz, $CDCl_3$) δ -57.7. **FT-IR** (cm^{-1}, neat, ATR), ṽ =1741, 1689, 1602, 1455, 1252, 1205, 1158,1083, 1036, 976, 946, 924, 850, 810, 507. **HRMS (ESI)** calcd for $C_{12}H_{12}F_3O_4$ $[M+H]^+$: 277.0682, found 277.0687.

Methyl 3-(4-Iodophenyl)-2-methyl-3-oxopropanoate (**1f**). **^{1}H NMR** (300 MHz, $CDCl_3$) δ (ppm)= 7.78 (d, *J* = 8.7 Hz, 1H), 7.63 (d, *J* = 8.7 Hz, 1H), 4.32 (q, *J* = 7.1 Hz, 1H), 3.63 (s, 3H), 1.41 (s, 3H). **^{13}C {^{1}H} NMR** (75 MHz, $CDCl_3$) δ 195.0, 170.9, 138.0, 135.0, 129.9, 101.7, 52.5, 47.9, 13.7. **FT-IR** (cm^{-1}, neat, ATR), ṽ =1736, 1682, 1579, 1559, 1452, 1434, 1391, 1375, 1272, 1196, 1080, 1058, 1005, 973, 942, 835, 749, 725, 586, 457. **HRMS (ESI+)** calcd for $C_{11}H_{12}IO_3$ $[M+H]^+$: 318.9826, found 318.9830.

Methyl 2-Methyl-3-oxo-3-(4-(trifluoromethyl)phenyl)propanoate (**1g**): According to the general procedure, 1.97 g of **1g** were synthesized from 10 mmol of starting material (76% yield with two steps) as a

dark brown oil after purification on silica gel (hexane: ethyl acetate = 9:1). **^{1}H NMR** (600 MHz, Chloroform-*d*) δ 8.07 (d, *J* = 8.4 Hz, 2H), 7.73 (d, *J* = 8.4 Hz, 2H), 4.41 (q, *J* = 7.0 Hz, 1H), 3.68 (d, *J* = 1.8 Hz, 3H), 1.49 (s, 3H). **^{19}F NMR** (282 MHz, Chloroform-*d*) δ -63.3. The characterization data is consistent with reported literature.[8]

Methyl 2-Methyl-3-(naphthalen-2-yl)-3-oxopropanoate (**1h**): According to the general procedure, 1.81 g of **1h** were synthesized from 10 mmol of starting material (75% yield with two steps) as a pale brown oil after purification on silica gel (hexane: ethyl acetate = 9:1). **^{1}H NMR** (300 MHz, Chloroform-*d*) δ 8.53 (d, *J* = 1.8 Hz, 1H), 8.11 – 7.87 (m, 5H), 7.68 – 7.57 (m, 2H), 4.60 (q, *J* = 7.1 Hz, 1H), 3.72 (s, 3H), 1.59 (d, *J* = 7.1 Hz, 3H). The characterization data is consistent with reported literature.[9]

Methyl 2-Methyl-3-oxo-3-(o-tolyl)propanoate (**1i**): According to the general procedure, 1.36 g of **1i** were synthesized from 10 mmol of starting material (66% yield with two steps) as a pale green oil after purification on silica gel (hexane: ethyl acetate = 9:1). **^{1}H NMR** (500 MHz, Chloroform-*d*) δ 7.65 (dd, *J* = 8.0, 1.5 Hz, 1H), 7.40 (td, *J* = 7.5, 1.4 Hz, 1H), 7.31 – 7.25 (m, 2H), 4.31 (q, *J* = 7.1 Hz, 1H), 3.69 (s, 3H), 2.50 (s, 3H), 1.47 (d, *J* = 7.2 Hz, 3H). The characterization data is consistent with reported literature.[6]

Ethyl 2-Methyl-3-oxo-3-phenylpropanoate (**1j**): According to the general procedure, 1.81 g of **1j** were synthesized from 10 mmol of starting material (88% yield) as a colorless solid after purification on silica gel (hexane: ethyl acetate = 10:1). **^{1}H NMR** (300 MHz, Chloroform-*d*) δ 8.02 – 7.93 (m, 2H), 7.62 – 7.54 (m, 1H), 7.51 – 7.43 (m, 2H), 4.37 (q, *J* = 7.1 Hz, 1H), 4.14 (q, *J* = 7.1 Hz, 2H), 1.49 (d, *J* = 7.1 Hz, 3H), 1.16 (t, *J* = 7.1 Hz, 3H). The characterization data is consistent with reported literature.[5]

Benzyl 2-Methyl-3-oxo-3-phenylpropanoate (**1k**): According to the general procedure, 1.87 g of **1k** were synthesized from 10 mmol of starting material (70% yield with two steps) as a colorless oil after purification on silica gel (hexane: ethyl acetate = 9:1).**^{1}H NMR** (300 MHz, Chloroform-*d*) δ 8.02 – 7.93 (m, 2H), 7.65 – 7.54 (m, 1H), 7.52 – 7.43 (m, 2H), 7.35 – 7.27 (m, 3H), 7.26 – 7.18 (m, 2H), 5.16 (d, *J* = 1.1 Hz, 2H), 4.45 (q, *J* = 7.1 Hz, 1H), 1.54 (s, 3H). The characterization data is consistent with reported literature.[6]

Isopropyl 3-(3,4-Dimethoxyphenyl)- 2-methyl-3-oxopropanoate (**1l**): According to the general procedure, 1.82 g of **1l** were synthesized from 10 mmol of starting material (65% yield with two steps) as a pale yellow after purification on silica gel (hexane: ethyl acetate = 9:1). **^{1}H NMR** (400 MHz, Chloroform-*d*) δ 7.52 (dd, *J* = 8.5, 2.1 Hz, 1H), 7.45 (d, *J* = 2.1 Hz, 1H), 6.80 (d, *J* = 8.4 Hz, 1H), 4.91 (p, *J* = 6.3 Hz, 1H), 4.21 (q, *J* = 7.0 Hz, 1H), 3.84 (d, *J* = 6.8 Hz, 6H), 1.37 (d, *J* = 7.1 Hz, 3H), 1.06 (dd, *J* = 9.9, 6.3 Hz, 6H). The characterization data is consistent with reported literature.[3]

2-Adamantyl 3-(3,4-Dimethoxyphenyl)-2-methyl-3-oxopropanoate (**1m**): According to the general procedure, 2.81 g of **1m** were synthesized from 10 mmol of starting material (64% yield with two steps) as a colorless solid after purification on silica gel (hexane: ethyl acetate = 9:1). **^{1}H NMR** (300 MHz, Chloroform-

d) δ 7.64 (dd, J = 8.4, 2.1 Hz, 1H), 7.57 (d, J = 2.0 Hz, 1H), 6.91 (d, J = 8.4 Hz, 1H), 4.23 (q, J = 7.0 Hz, 1H), 3.96 (d, J = 5.1 Hz, 6H), 2.13 (d, J = 4.0 Hz, 3H), 2.02 (d, J = 3.2 Hz, 6H), 1.62 (d, J = 3.0 Hz, 6H), 1.45 (d, J = 7.0 Hz, 3H). The characterization data is consistent with reported literature.[1]

Methyl 2-Benzoyl-2-(trifluoromethyl)octadecenoate (**1n**): According to the general procedure, 2.73 g of **1n** were synthesized from 10 mmol of starting material (68% yield with two steps) as a pale-yellow solid after purification on silica gel (hexane: ethyl acetate = 9:1). **^{1}H NMR** (400 MHz, Chloroform-*d*) δ 7.96 – 7.89 (m, 2H), 7.50 (t, J = 7.4 Hz, 1H), 7.40 (t, J = 7.7 Hz, 2H), 4.26 (t, J = 7.2 Hz, 1H), 3.60 (s, 3H), 1.94 (hept, J = 6.8 Hz, 2H), 1.19 (d, J = 6.4 Hz, 28H), 0.81 (t, J = 6.8 Hz, 3H). **^{13}C NMR** (151 MHz, Chloroform-*d*) δ 195.2, 170.5, 136.2, 133.5, 133.4, 128.8, 128.6, 128.5, 54.1, 53.9, 52.4, 52.3, 31.9, 29.7, 29.6, 29.5, 29.4, 29.3, 29.3, 29.1, 28.9, 27.6, 22.7, 14.1. The characterization data is consistent with reported literature.[11]

methyl 2-Benzoyl-3-methylbutanoate (**1o**): According to the general procedure, 1.25 g of **1o** were synthesized from 10 mmol of starting material (57% yield with two steps) as a pale green oil after purification on silica gel (hexane: ethyl acetate = 9:1). **^{1}H NMR** (600 MHz, Chloroform-*d*) δ 8.05 – 8.00 (m, 2H), 7.61 – 7.56 (m, 1H), 7.50 – 7.45 (m, 2H), 4.14 (d, J = 9.4 Hz, 1H), 3.67 (s, 3H), 2.66 (dp, J = 9.4, 6.7 Hz, 1H), 1.05 (d, J = 6.7 Hz, 3H), 0.94 (d, J = 6.7 Hz, 3H). The characterization data is consistent with reported literature.[4]

Methyl 2-Benzoylpent-4-enoate (**1p**): According to the general procedure, 1.26 g of **1p** were synthesized from 10 mmol of starting material (58% yield with two steps) as a pale brown oil after purification on silica gel (hexane: ethyl acetate = 9:1). **^{1}H NMR** (600 MHz, Chloroform-*d*) δ 8.04 – 7.99 (m, 2H), 7.64 – 7.58 (m, 1H), 7.53 – 7.47 (m, 2H), 5.83 (ddt, J = 17.0, 10.2, 6.9 Hz, 1H), 5.13 (dq, J = 17.0, 1.5 Hz, 1H), 5.06 (dq, J = 10.2, 1.3 Hz, 1H), 4.45 (t, J = 7.2 Hz, 1H), 3.70 (s, 3H), 2.78 (dtt, J = 9.1, 7.8, 6.2 Hz, 2H). The characterization data is consistent with reported literature.[7]

Methyl 2-Benzyl-3-oxo-3-phenylpropanoate (**1q**): According to the general procedure, 1.63g of **1q** were synthesized from 10 mmol of starting material (61% yield with two steps) as a pale brown oil after purification on silica gel (hexane: ethyl acetate = 9:1). **^{1}H NMR** (300 MHz, Chloroform-*d*) δ 8.05 – 7.95 (m, 2H), 7.65 – 7.54 (m, 1H), 7.54 – 7.41 (m, 2H), 7.33 – 7.17 (m, 5H), 4.72 (t, J = 7.3 Hz, 1H), 3.66 (s, 3H), 3.38 (dd, J = 7.3, 3.5 Hz, 2H). The characterization data is consistent with reported literature.[6]

2-Methyl-1-(naphthalen-2-yl)butane-1,3-dione (**1r**): According to the general procedure, 1.74 g of **1r** were synthesized from 10 mmol of starting material (77% yield with two steps) as a pale yellow solid after purification on silica gel (hexane: ethyl acetate = 9:1). **^{1}H NMR** (400 MHz, Chloroform-*d*) δ 8.49 – 8.44 (m, 1H), 8.03 – 7.81 (m, 4H), 7.56 (dddd, J = 21.9, 8.1, 6.9, 1.3 Hz, 2H), 4.61 (q, J = 7.0 Hz, 1H), 2.15 (s, 3H), 1.48 (d, J = 7.0 Hz, 3H). The characterization data is consistent with reported literature.[10]

2-Methyl-1,3-diphenyl-1,3-propanedione (**1s**): According to the general procedure, 1.26 g of **1s** were synthesized from 6.25 mmol of starting material (85% yield) as a colorless solid after purification on silica gel (hexane: ethyl acetate = 9:1). **^{1}H NMR** (400 MHz, Chloroform-*d*) δ 7.91 (dd, J = 8.4, 1.5 Hz, 4H), 7.52 – 7.44

(m, 2H), 7.37 (t, *J* = 7.7 Hz, 4H), 5.27 (q, *J* = 7.0 Hz, 1H), 1.53 (d, *J* = 7.1 Hz, 3H). The characterization data is consistent with reported literature.[5]

Ethyl 2-Methyl-3-oxo-3-(pyridin-4-yl)propanoate (**1t**): According to the general procedure, 1.34 g of **1t** were synthesized from 10 mmol of starting material (65% yield with one step) as a brown oil after purification on silica gel (hexane: ethyl acetate = 9:1). ^{1}H NMR (300 MHz, Chloroform-*d*) δ 8.79 – 8.71 (m, 2H), 7.72 – 7.64 (m, 2H), 4.27 (q, *J* = 7.1 Hz, 1H), 4.07 (q, *J* = 7.1 Hz, 2H), 1.42 (s, 3H), 1.09 (t, *J* = 7.1 Hz, 3H). ^{13}C NMR (75 MHz, Chloroform-*d*) δ 195.3, 170.0, 150.9, 141.9, 121.3, 61.6, 48.6, 13.8, 13.2. **HRMS (ESI)** calcd for $C_{11}H_{13}NO_3Na$ [M+Na]$^+$: 230.0788, found 230.0791.

Ethyl 3-(Furan-2-yl)-2-methyl-3-oxopropanoate (**1v**): According to the general procedure, 1.58 g of **1v** were synthesized from 10 mmol of starting material (81% yield with one step) as a pale green oil after purification on silica gel (hexane: ethyl acetate = 9:1). ^{1}H NMR (600 MHz, Chloroform-*d*) δ 7.81 (dd, *J* = 3.8, 1.1 Hz, 1H), 7.71 (dd, *J* = 4.9, 1.1 Hz, 1H), 7.17 (dd, *J* = 4.9, 3.8 Hz, 1H), 4.25 (q, *J* = 7.1 Hz, 1H), 4.19 (q, *J* = 7.1 Hz, 2H), 1.52 (s, 3H), 1.22 (t, *J* = 7.1 Hz, 3H). The characterization data is consistent with reported literature.[16]

Ethyl 2-Methyl-3-oxo-3-(thiophen-2-yl)propanoate (**1u**): According to the general procedure, 1.55 g of **1u** were synthesized from 10 mmol of starting material (73% yield with one step) as a pale-yellow oil after purification on silica gel (hexane: ethyl acetate = 9:1). ^{1}H NMR (600 MHz, Chloroform-*d*) δ 7.81 (dd, *J* = 3.8, 1.1 Hz, 1H), 7.71 (dd, *J* = 4.9, 1.1 Hz, 1H), 7.17 (dd, *J* = 4.9, 3.8 Hz, 1H), 4.25 (q, *J* = 7.1 Hz, 1H), 4.19 (q, *J* = 7.1 Hz, 2H), 1.52 (s, 1H), 1.22 (t, *J* = 7.1 Hz, 3H). The characterization data is consistent with reported literature.[16]

Ethyl 2-Methyl-3-oxo-5-phenylpent-4-ynoate (**1w**): According to the general procedure, 1.66 g of **1w** were synthesized from 10 mmol of starting material (72% yield with one step) as a yellow solid after purification on silica gel (hexane: ethyl acetate = 9:1). ^{1}H NMR (600 MHz, Chloroform-*d*) δ 12.22 (s, 1H), 7.64 – 7.55 (m, 2H), 7.49 – 7.37 (m, 3H), 4.30 (q, *J* = 7.1 Hz, 2H), 2.03 (s, 3H), 1.37 (t, *J* = 7.1 Hz, 3H).[17]

***S*-(Trifluoromethyl)thianthrenium triflate** (**2**): **^{1}H NMR** (300 MHz, Chloroform-*d*) δ 8.61 (dd, *J* = 8.0, 1.4 Hz, 2H), 7.93 (ddd, *J* = 8.5, 7.3, 1.4 Hz, 2H), 7.87 – 7.73 (m, 4H). **^{19}F NMR** (282 MHz, Chloroform-*d*) δ -51.1, -78.4. The characterization data is consistent with reported literature.[12]

2. Trifluoromethylation of α-Substituted β-Keto Esters and β-Diketones. Optimization Process and Compound Characterization

2.1. Reaction Optimization

3-Oxo-3-phenylpropanoate (**1a**) and TT-$CF_3^+OTf^-$ (**2**) were selected as model substrates (Table S1). After preliminary experimentation, compound **3a** was obtained in a 15% yield using DCM as the solvent and NaH as the base at room temperature (Table S1, entries 1–10). The presence of a base was essential for facilitating the transformation (Table 1, entry 11). Next, we explored various organic and inorganic bases, including K_2CO_3, NEt_3, DMAP, DBU, KO^{t}Bu, and quinine, observing the highest yield with KO^{t}Bu (Table 1, entries 12–17). Increasing the reaction temperature to 35 °C further improved the yield. Finally, an 80% yield of **3a** was achieved by increasing the equivalents of both substrate **2** and KO^{t}Bu to 1.5. Thus, the optimized reaction conditions involve using DCM as the solvent, 1.5 equivalents of **2**, and 1.5 equivalents of KO^{t}Bu at 35 °C for 10 minutes (Table S1, entry 20).

Table S1. Optimization of reaction conditions[a,b]

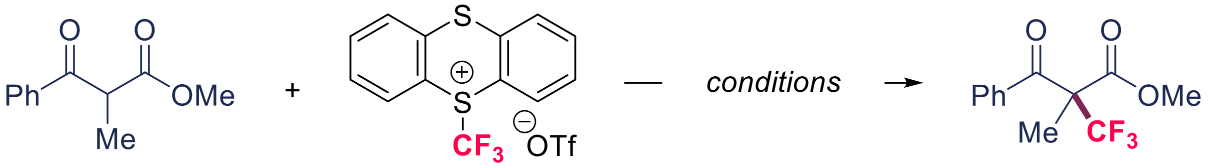

1a **2** **3**

Entry	Equiv **1a**	Equiv **2**	Solvent	Base (equiv)	Temperature (°C)	Isolated Yield of **3**
1	1	1.2	DMF	NaH (1.2)	rt	0
2	1	1.2	Acetone	NaH (1.2)	rt	0
3	1	1.2	THF	NaH (1.2)	rt	10%
4	1	1.2	MeCN	NaH (1.2)	rt	0
5	1	1.2	Mesitylene	NaH (1.2)	rt	0
6	1	1.2	DMSO	NaH (1.2)	rt	8%
7	1	1.2	DCM	NaH (1.2)	rt	15%
8	1	1.2	Dioxane	NaH (1.2)	rt	5%
9	1	1.2	DCE	NaH (1.2)	rt	0

10	1	1.2	Chloroform	NaH (1.2)	rt	0
11	1	1.2	DCM	none	rt	0
12	1	1.2	DCM	K_2CO_3 (1.2)	rt	0
13	1	1.2	DCM	TEA (1.2)	rt	0
14	1	1.2	DCM	DMAP (1.2)	rt	0
15	1	1.2	DCM	DBU (1.2)	rt	40%
16	1	1.2	DCM	KO^{t}Bu (1.2)	rt	50%
17	1	1.2	DCM	Quinine (1.2)	rt	0
18	1	1.2	DCM	KO^{t}Bu (1.2)	-55℃	35%
19	1	1.2	DCM	KO^{t}Bu (1.2)	35℃	60%
20	1	1.5	DCM	KO^{t}Bu (1.5)	35℃	80%
21[b]	1	1.5	DCM	KO^{t}Bu (1.5)	35℃	0%
22	1	1.5	MeCN	KO^{t}Bu (1.5)	35℃	42%

[a]Reaction conditions: **1a** (0.2 mmol, 1.0 equiv.), TT-CF_3^+OTf$^-$ **2** (1.2 equiv.), solvent (2 mL). [b]Isolated yield based on **1a**.[b]Using Umemoto's reagent.

3. Data of X-Ray Structure of Compound 20

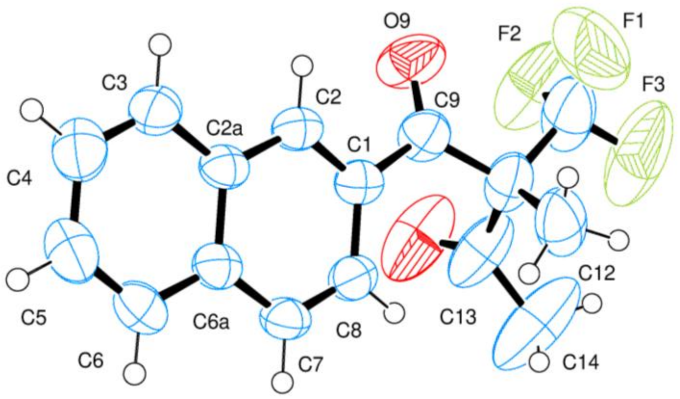

Figure S1. X-Ray crystal structure of **20**. Ortep view of **20** showing the atom labelling and the thermal vibration ellipsoids at 50% probability.

Adequate crystals were obtained by crystallization from DCM for compound **20**. Data was collected using Mo Kα radiation in a SMART-APEX. An empirical absorption correction was applied (SADABS). The structure was solved by direct methods (SHELXS-86) and refined by full-matrix least-squares methods on F2 for all S35 S36 reflections (SHELXL- 2016). Non-hydrogen atoms were refined anisotropically. Hydrogen atoms bonded to carbon atoms were placed in calculated positions with isotropic displacement parameters fixed at 1.2 times the Ueq of the corresponding carbon atoms. Crystal data and further refinement details are presented in Table S2.

Table S2. Crystal data and structure refinement for compound **20**.

Empirical formula	$C_{16}H_{13}F_3O_2$
Formula weight	294.26
Temperature	297(2) K
Wavelength	0.71073 Å
Crystal system	Orthorhombic
Space group	P 21 21 21
Unit cell dimensions	a = 6.3550(8) Å α= 90°.
	b = 7.2015(8) Å β= 90°.

	c = 30.770(4) Å γ = 90°.
Volume	1408.2(3) $Å^3$
Z	4
Density (calculated)	1.388 Mg/m^3
Absorption coefficient	0.117 mm^{-1}
F(000)	608
Crystal size	0.38 x 0.28 x 0.09 mm^3
Theta range for data collection	1.324 to 28.493°.
Index ranges	-8<=h<=8, -9<=k<=9, -41<=l<=41
Reflections collected	41084
Independent reflections	3541 [R(int) = 0.0434]
Completeness to theta = 25.242°	100.0 %
Refinement method	Full-matrix least-squares on F^2
Data / restraints / parameters	3541 / 0 / 192
Goodness-of-fit on F^2	1.050
Final R indices [I>2 sigma(I)]	R1 = 0.0632, wR2 = 0.1732
R indices (all data)	R1 = 0.0879, wR2 = 0.1949
Absolute structure parameter	-0.1(3)
Largest diff. peak and hole	0.286 and -0.202 e.$Å^{-3}$

4. Mechanistic Investigation

4.1 UV-Vis studies

UV/vis absorption spectra were measured in a 1 cm quartz cuvette using a Genesys 150 UV/vis spectrophotometer from Thermo Scientific. Absorption spectra of individual reaction components and mixtures were recorded.

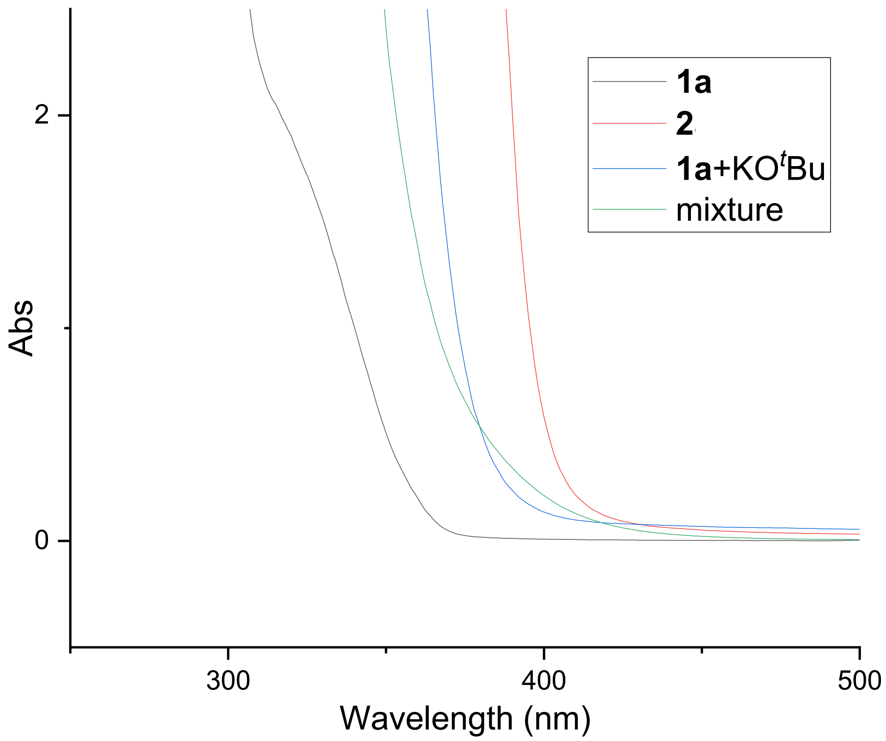

Figure S2. UV/vis absorption spectra of individual reaction components and a combination thereof. All spectra were measured in DCM and with a concentration of 0.02 M methyl 2-methyl-3-oxo-3-phenylpropanoate (**1a**), 0.03M of *S*-(trifluoromethyl)thianthrenium triflate (**2**, $TTCF_3^+OTf^-$), and 0.03 M KO^{t}Bu. The stoichiometry reflects the used reaction conditions.

4.2 Reaction of β-keto ester **1a** and **2** with radical scavenger Galvinoxyl.

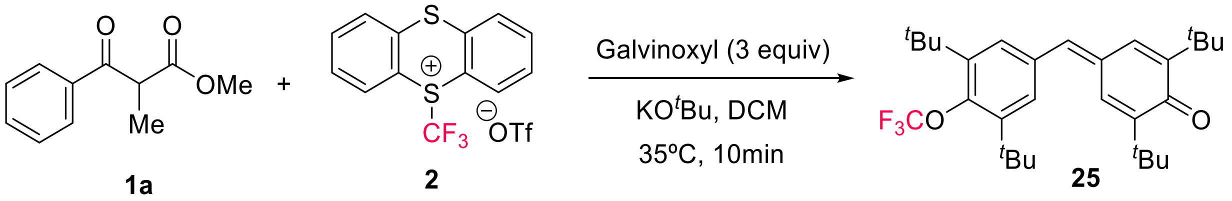

To a flame-dried 10 mL vial equipped with a magnetic stirring bar, methyl 2-methyl-3-oxo-3-phenylpropanoate **1a** (0.5 mmol, 1 equiv), the corresponding KO^{t}Bu (0.75 mmol, 1.5 equiv), 5 mL of anhydrous DCM were added. The vial was closed with a screwcap, and stirred for 5 minutes. Then, the Galvinoxyl and TT-$CF_3^+OTf^-$ **2** (0.75mmol, 1.5 equiv) was added separately. The reaction mixture was stirred vigorously at 35 °C. After10 min, the mixture was diluted with water and extracted with EtOAc (30 mL×3). The organics were combined, washed with water (15 mL) and brine (15 mL×2), and finally dried over anhydrous Na_2SO_4. After solvent removal under high vacuum, the product was purified by flash column chromatography through silica gel. The experiment adding Galvinoxyl free radical gave exclusively the corresponding CF_3 adduct **25** (18 %). The total amount of unreacted **1a** could be recovered.

2,6-Di-tert-butyl-4-((3,5-di-tert-butyl-4-oxocyclohexa-2,5-dien-1-ylidene)methyl)-4-(trifluoromethyl) cyclohexa-2,5-dien-1-one (**25**): **^{1}H NMR** (300 MHz, $CDCl_3$) δ 6.83 (s, 2H), 6.55 (s, 2H), 6.51 (s, 1H), 1.28 (s, 36H). **^{13}C{^{1}H} NMR** (151 MHz, CDCl3) δ 186.2, 184.8, 151.0, 150.4, 148.6, 135.8, 134.3, 133.7, 125.1(q, J = 285.4 Hz), 124.7, 35.5, 34.9, 29.3. **^{19}F NMR** (282 MHz, $CDCl_3$) δ -73.6. The data is in agreement with those previously reported.[15]

4.3 Evaluation of lanthanide catalysis.

To assess the feasibility of lanthanide-catalyzed conditions, we attempted the reaction under lanthanide catalysis. However, no product formation was observed, and the starting 3-oxoester remained unreacted in all cases.

This negative outcome further supports our hypothesis that the reaction proceeds via the addition of the enolate anion to the electrophilic sulfur (S) atom of reagent **2**, leading to the formation of a sulfurane intermediate. Under lanthanide-catalyzed conditions, the enolate preferentially coordinates to the metal center, thereby preventing nucleophilic attack on the sulfur atom and ultimately suppressing reactivity.

Table S3. Reaction conditions.

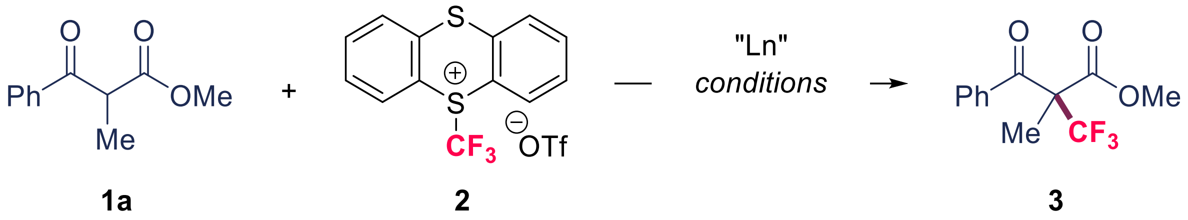

Entry	Equiv **1a**	Equiv **2**	Solvent	Ln(OTf)₃	Isolated Yield of **3**
1	1	1.2	DCM	La	0
2	1	1.2	DCM	Eu	0
3	1	1.2	DCM	Yb	0
4	1	1.2	MeCN	La	0
5	1	1.2	DMF	La	0

[a]Reaction conditions: **1a** (0.2 mmol, 1.0 equiv.), TT-$CF_3^+OTf^-$ **2** (1.2 equiv.), solvent (2 mL), $Ln(OTf)_3$ (1 equiv), 2,6-bis[(4*R*)-4-phenyl-2-oxazolinyl]pyridine (2 equiv). [b]Isolated yield based on **1a**.

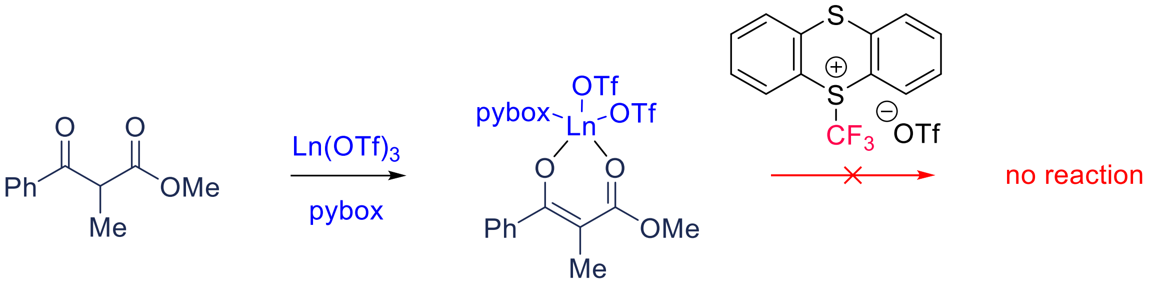

pybox = pyridine bis(oxazoline)

4.4 Detection of intermediates using HR-MS.

To a flame-dried 5 mL vial equipped with a magnetic stirring bar, 1,3-dicarbonyl compound **1a** (0.1 mmol, 1 equiv), KO*t*Bu (1.5 equiv) and 1 mL of anhydrous DCM were added. The vial was closed with a screwcap and stirred for 5 minutes. Then, the reaction mixture was cooled down to -35°C and TT-$CF_3^+OTf^-$ **2** (1.5 equiv) was added. The reaction mixture was stirred for 1 minute and dilutions in DCM were made prior to the injection in the mass spectrometer. The following species were detected in high resolution MS.

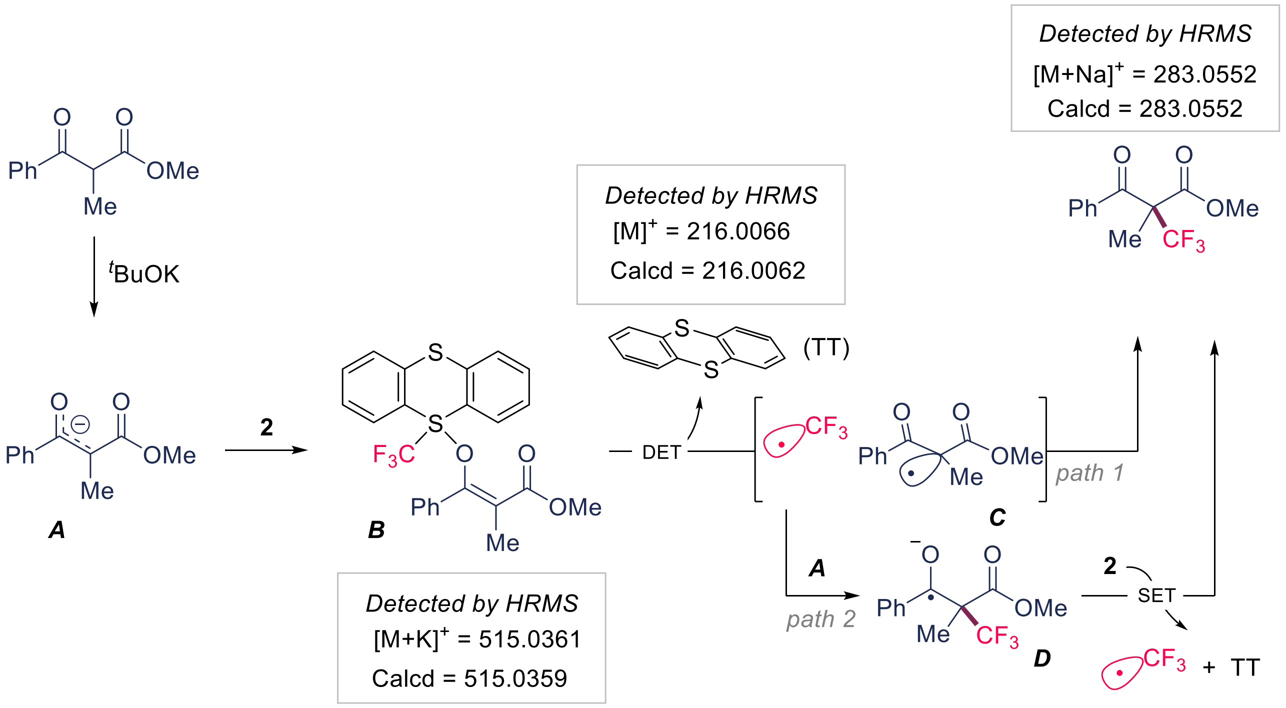

Scheme S1. Detected intermediates from the reaction mixture.

5. Unsuccessful Substrates

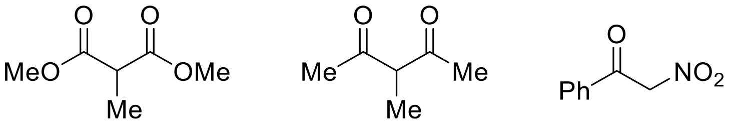

6. NMR Spectra

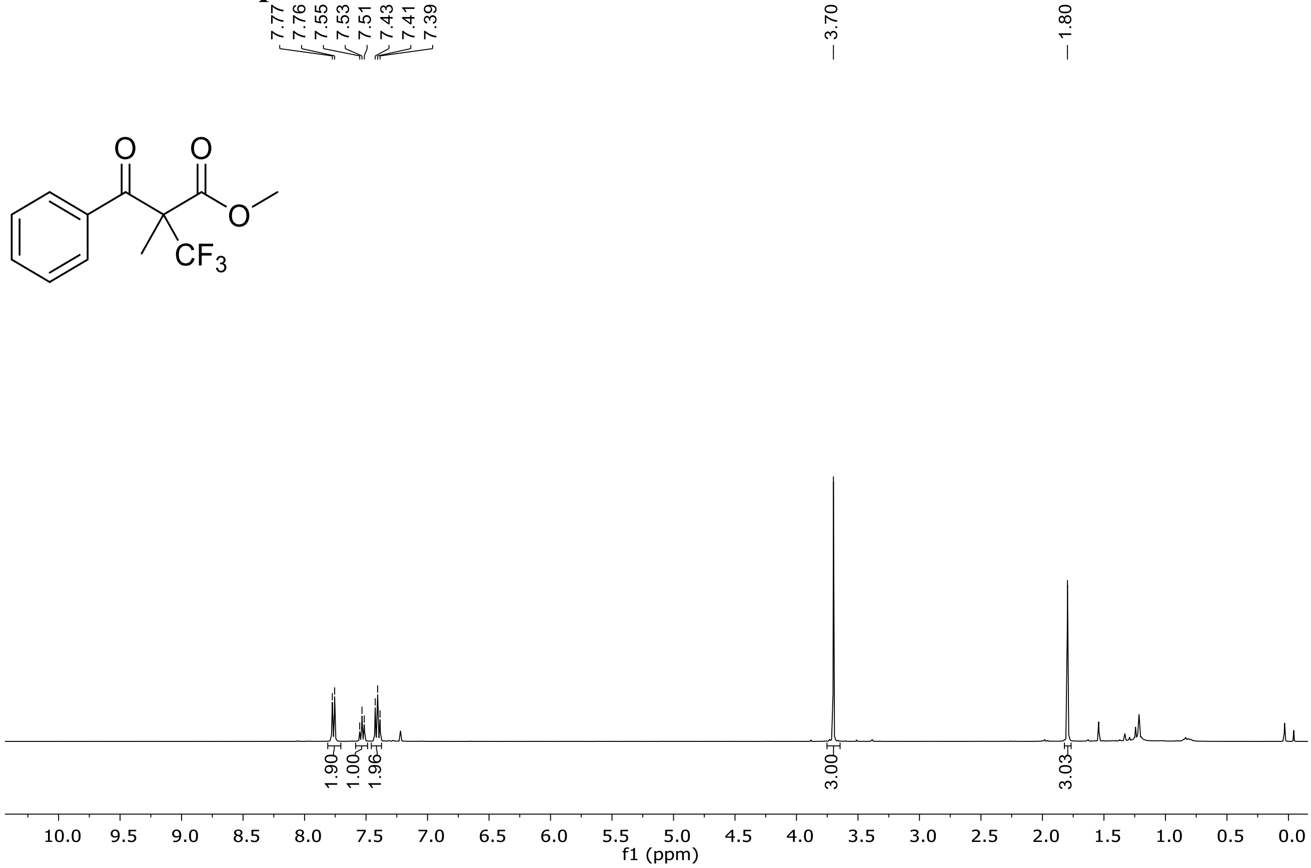

^{1}H NMR (400 MHz, $CDCl_3$) of compound **3**.

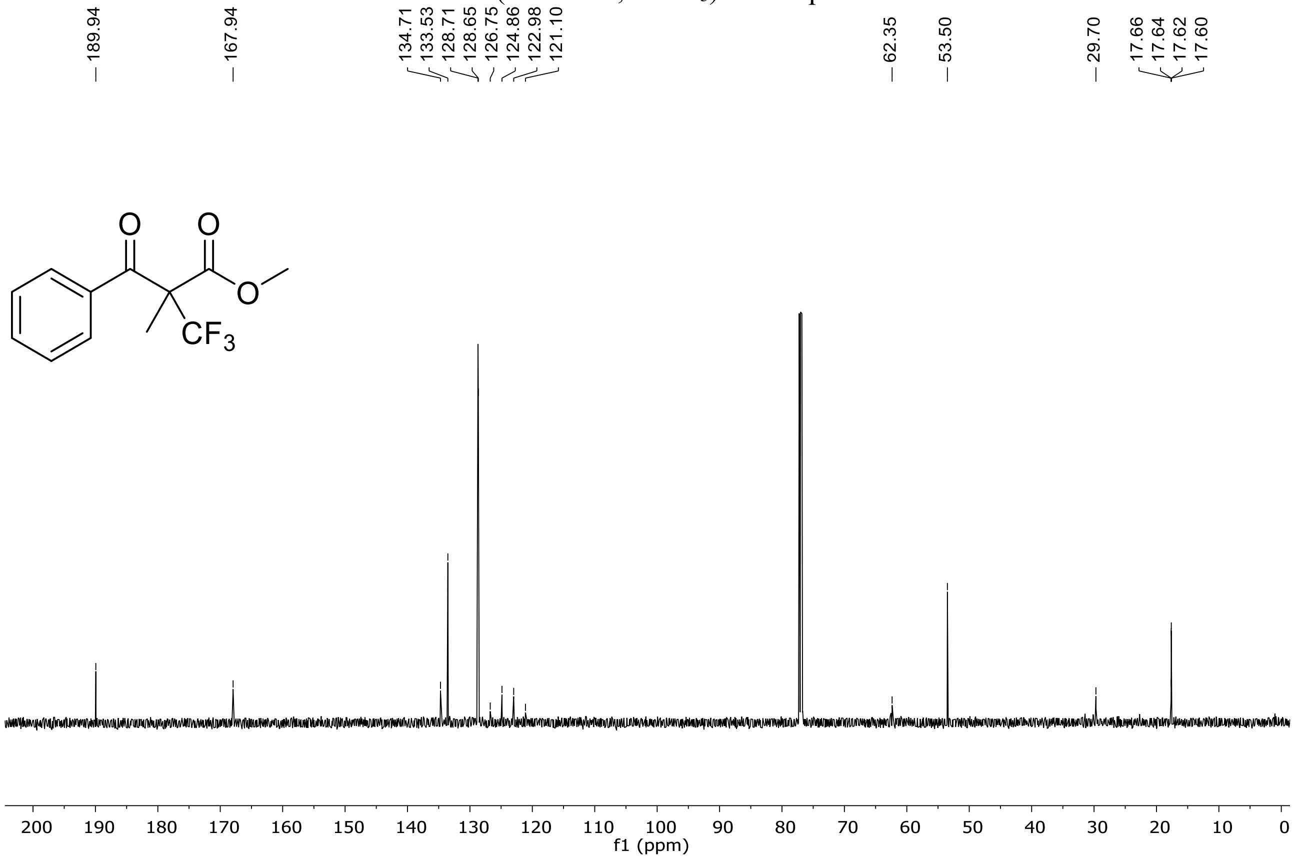

$^{13}C\{^{1}H\}$ NMR (151 MHz, $CDCl_3$) of compound **3**.

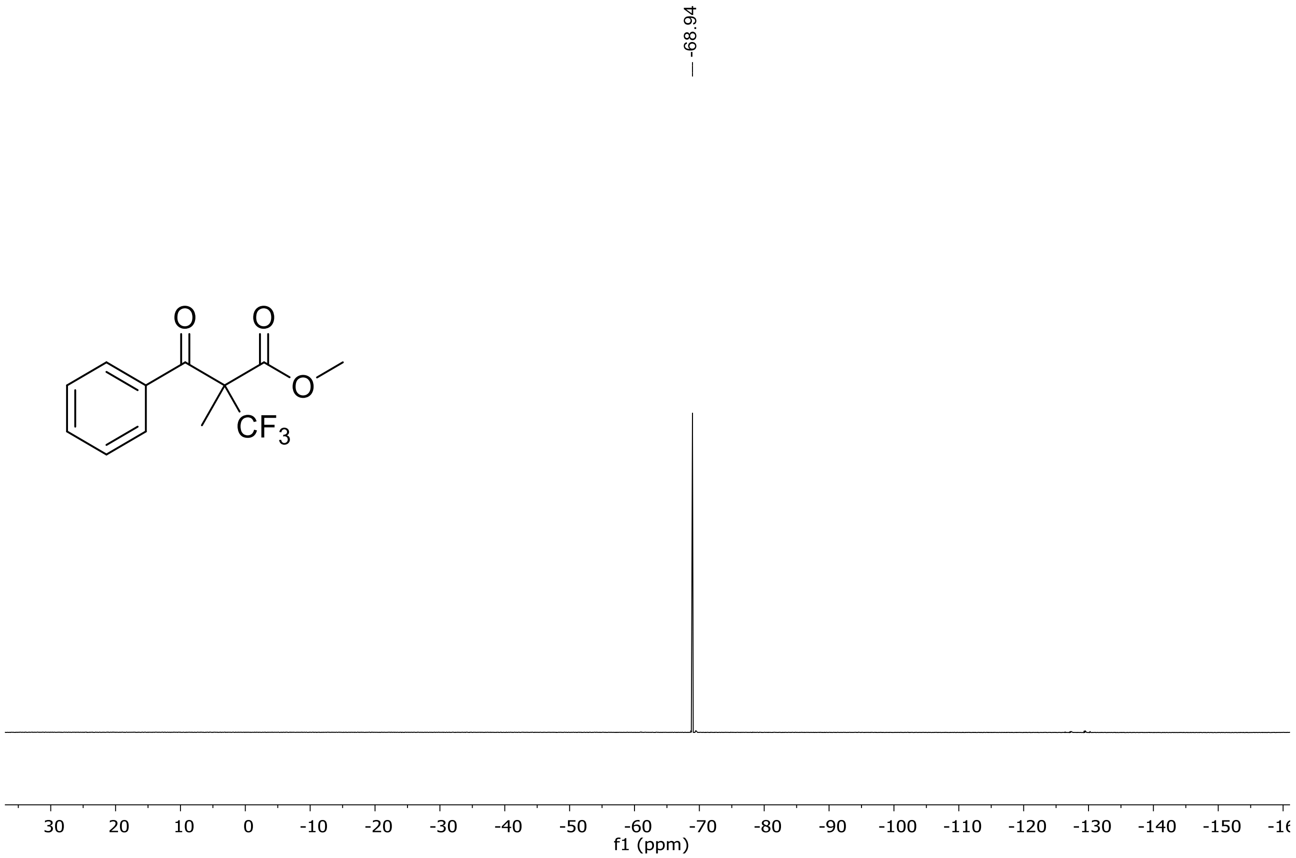

^{19}F NMR (377 MHz, $CDCl_3$) of compound **3**.

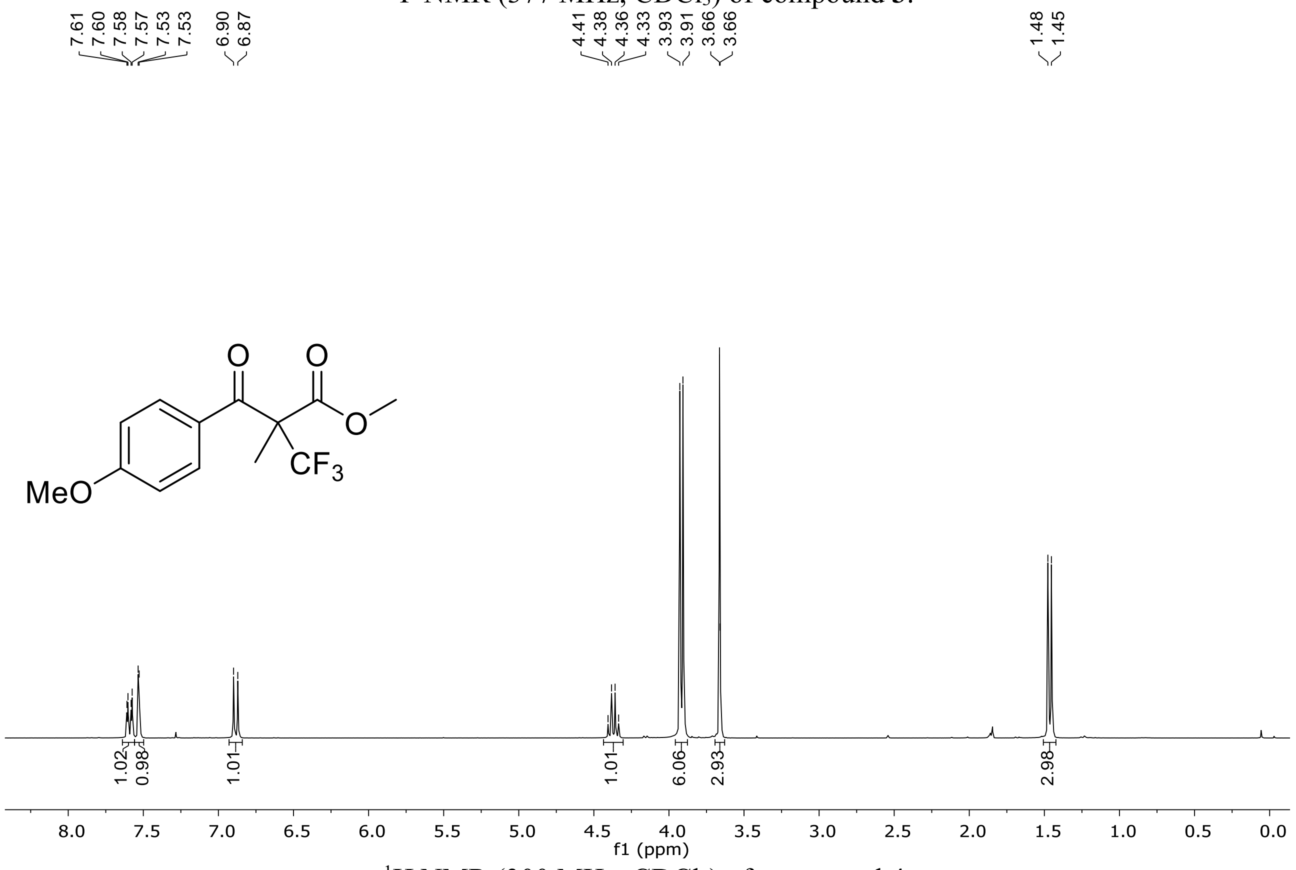

^{1}H NMR (300 MHz, $CDCl_3$) of compound **4**.

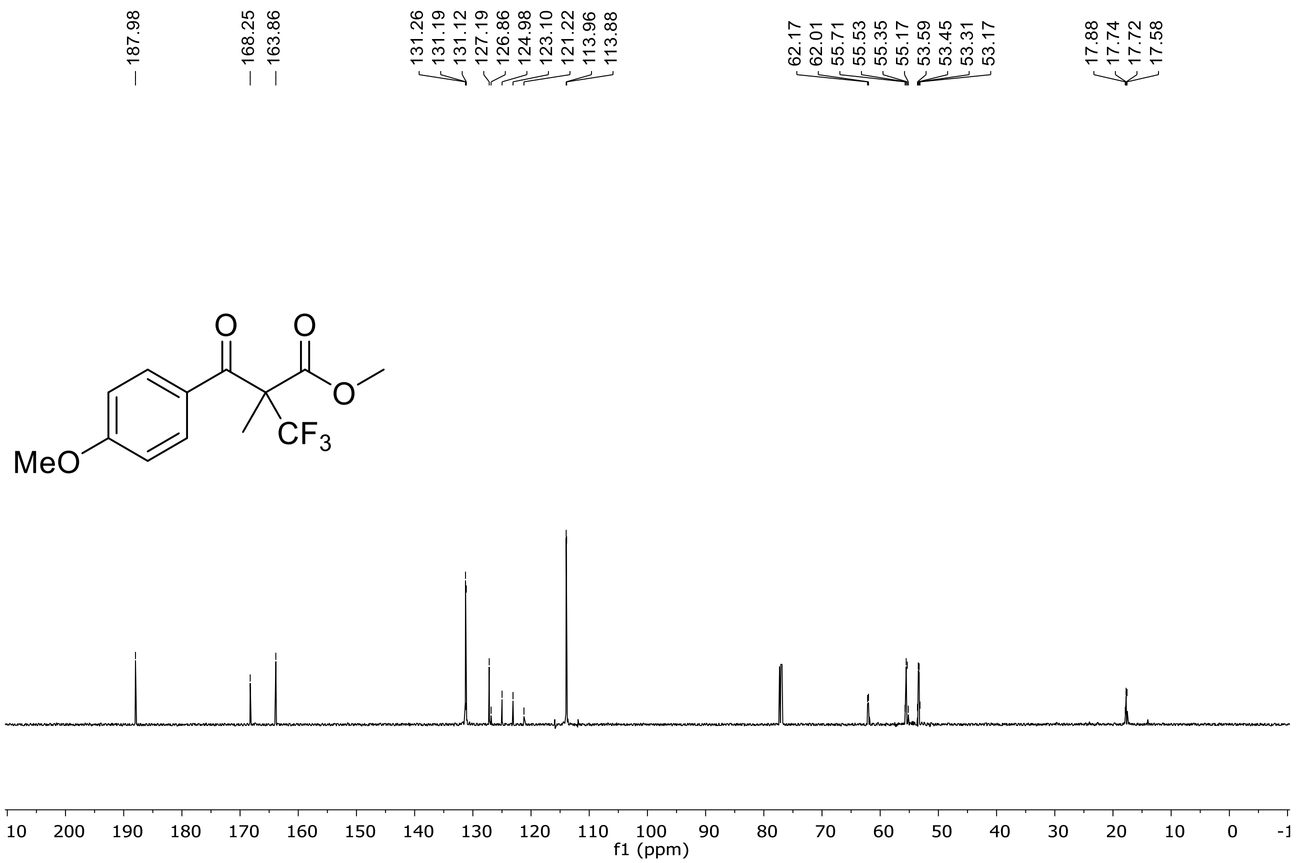

$^{13}C\{^1H\}$ NMR (151 MHz, $CDCl_3$) of compound **4**.

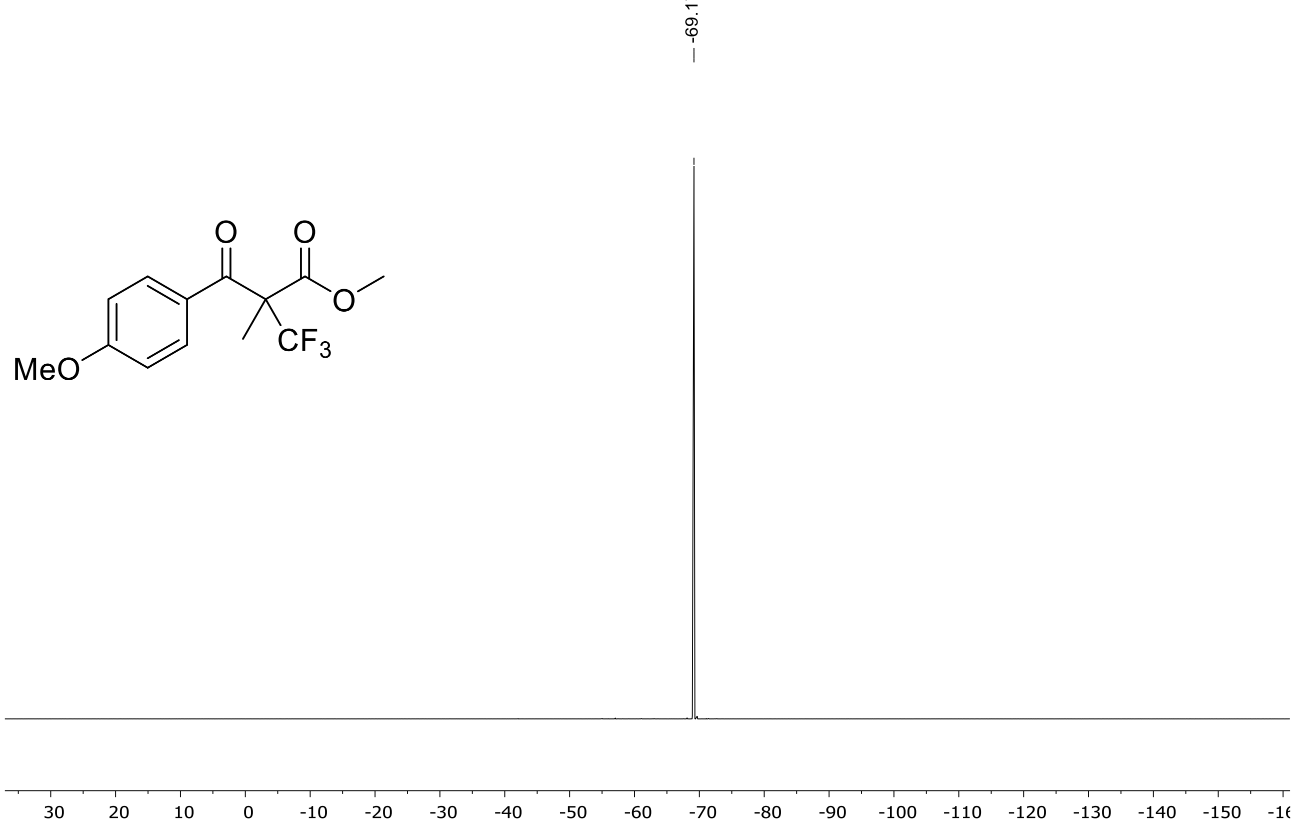

^{19}F NMR (282 MHz, $CDCl_3$) of compound **4**.

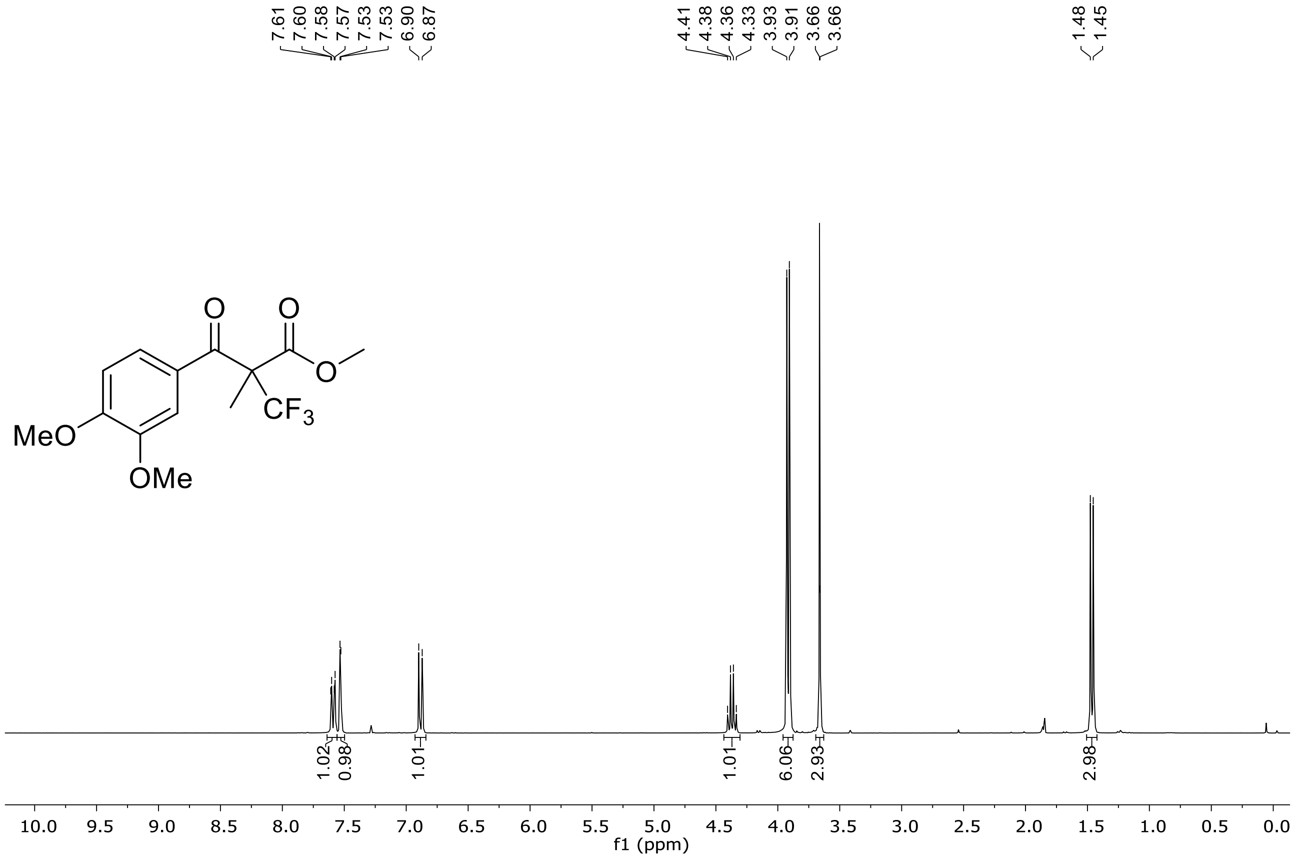

^{1}H NMR (600 MHz, $CDCl_3$) of compound **5**.

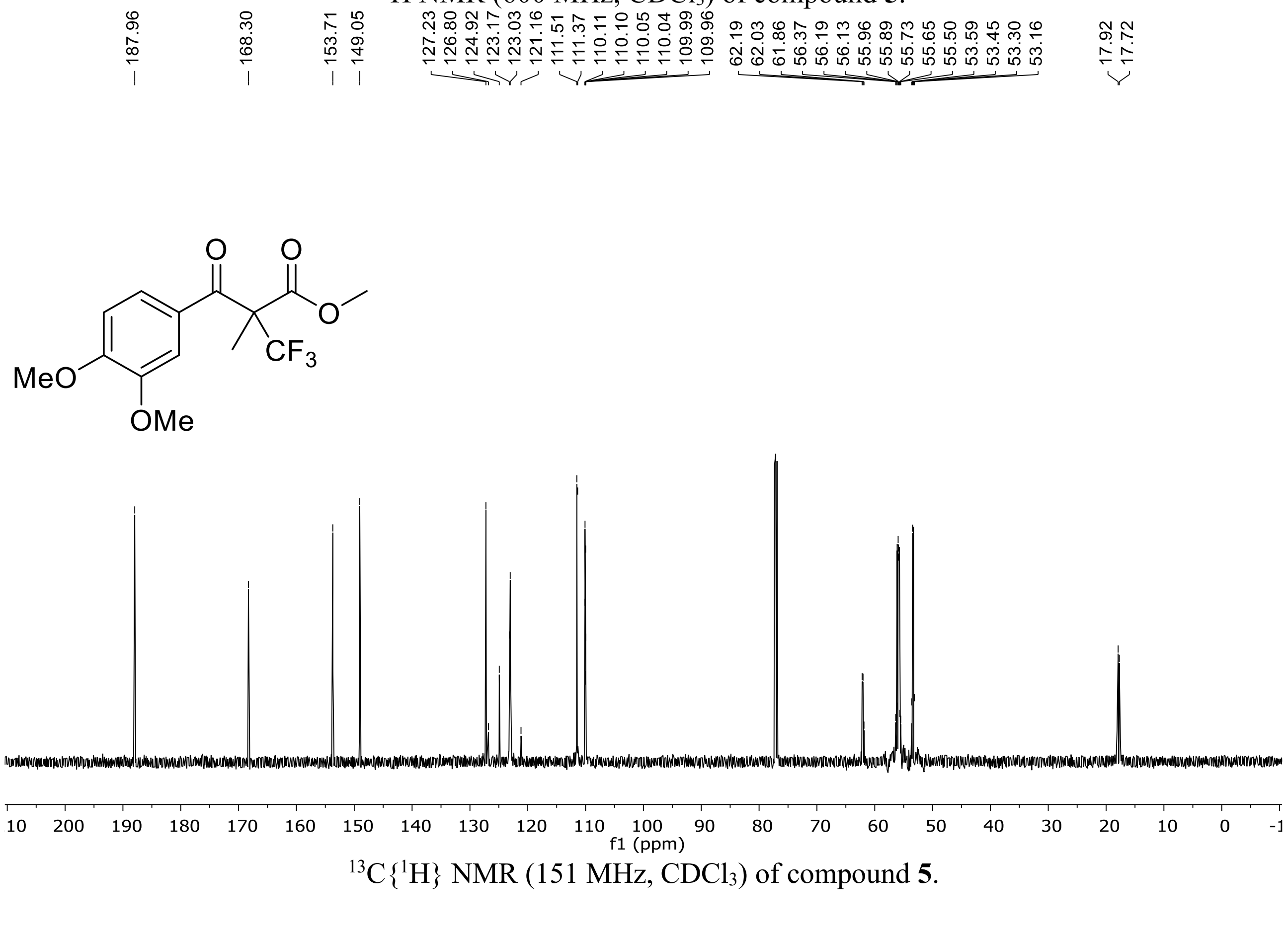

^{13}C{^{1}H} NMR (151 MHz, $CDCl_3$) of compound **5**.

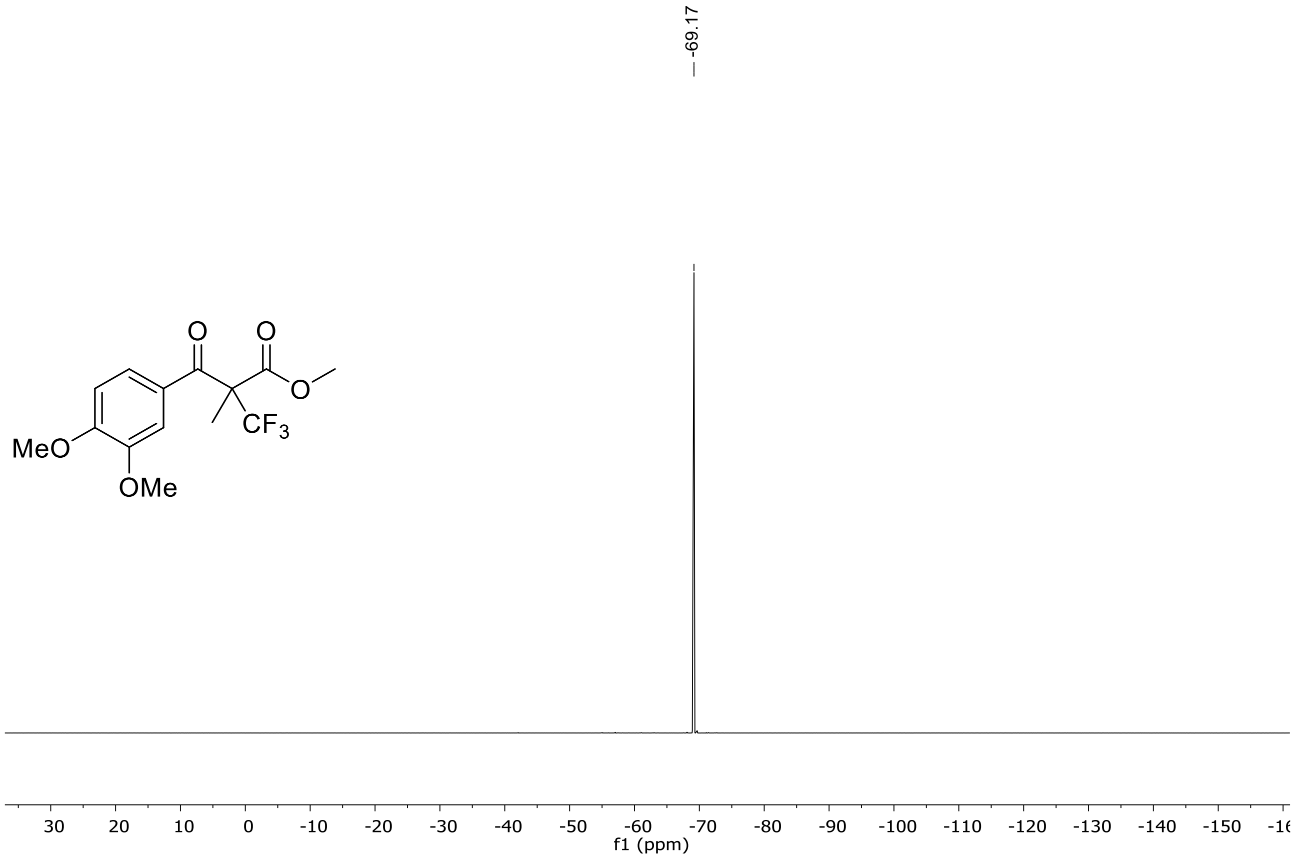

^{19}F NMR (377 MHz, $CDCl_3$) of compound **5**.

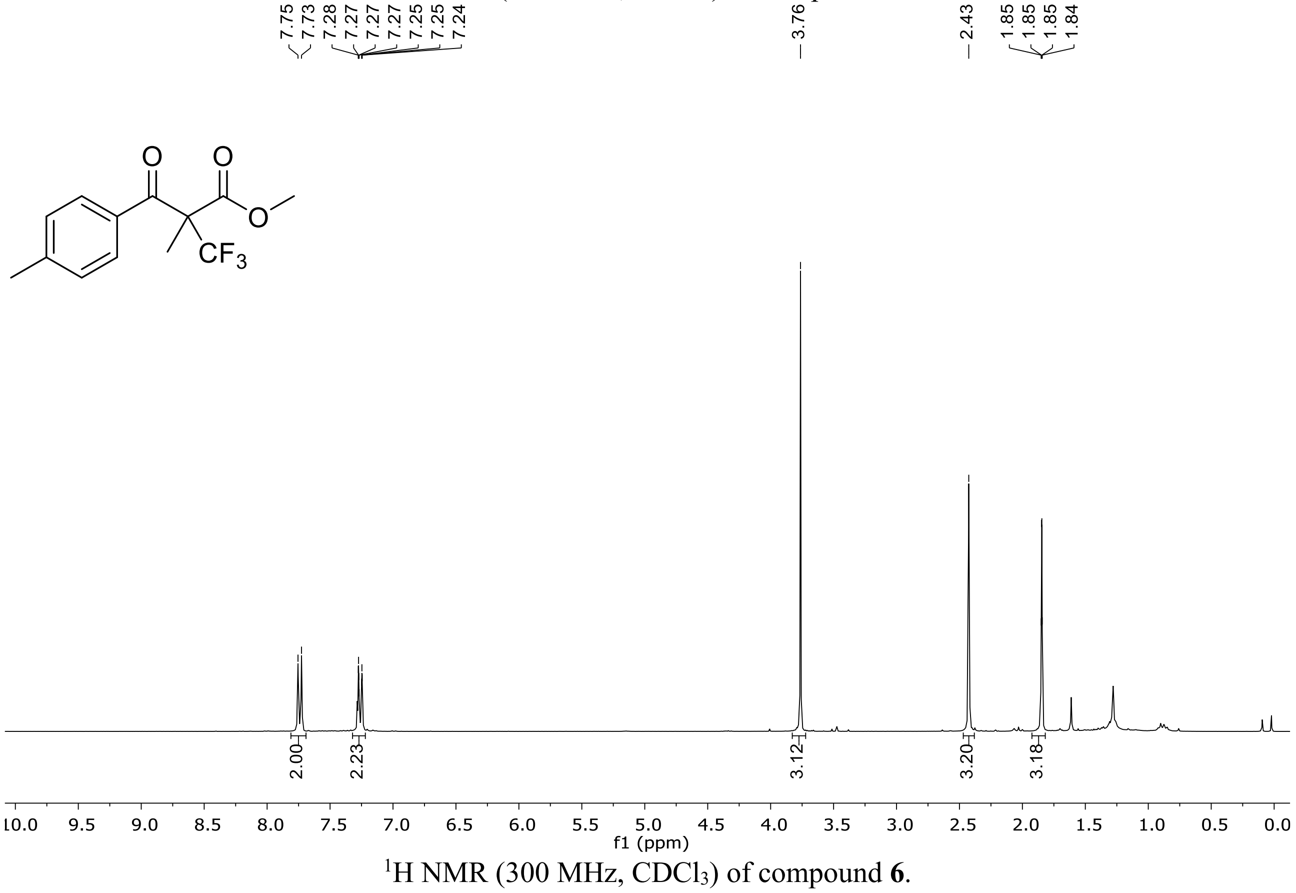

^{1}H NMR (300 MHz, $CDCl_3$) of compound **6**.

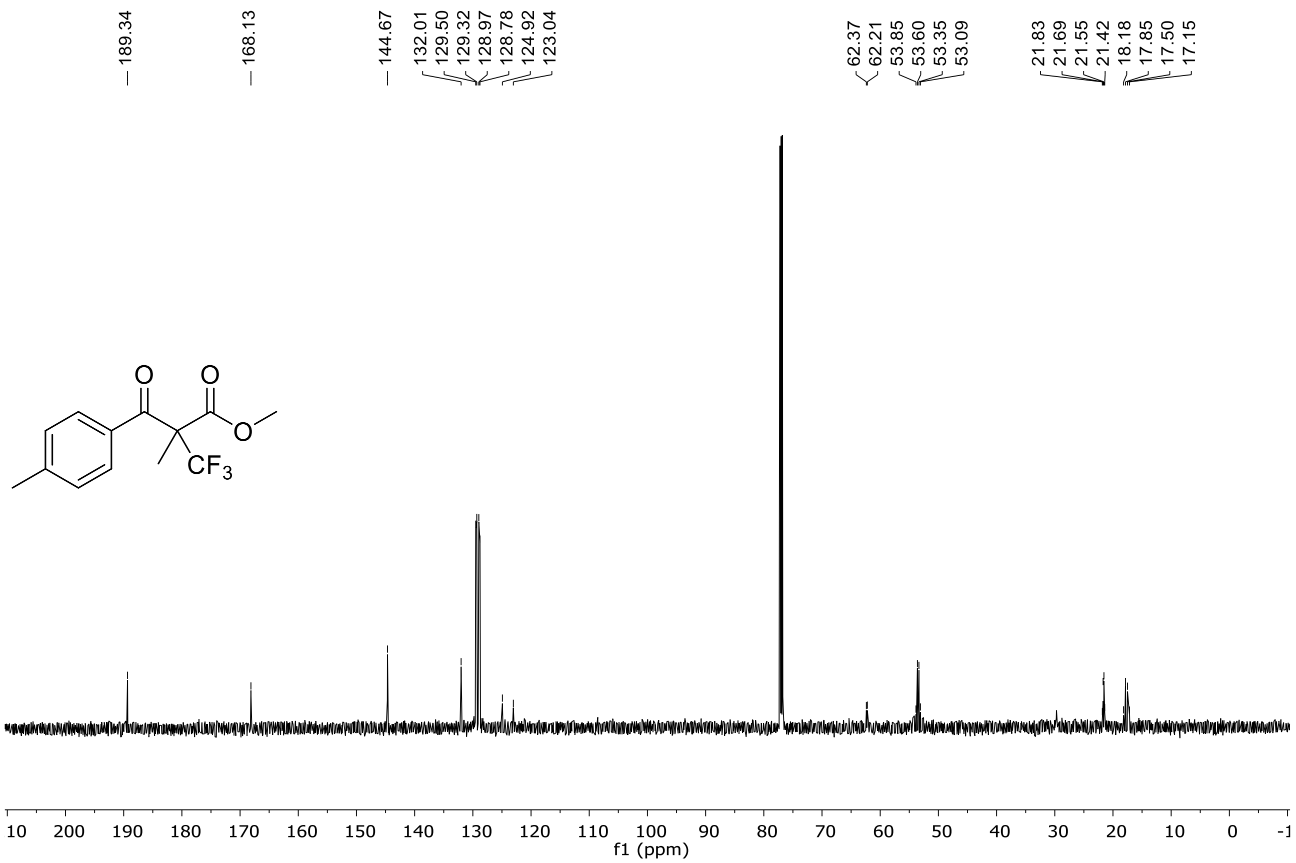

$^{13}C\{^{1}H\}$ NMR (151 MHz, $CDCl_3$) of compound **6**.

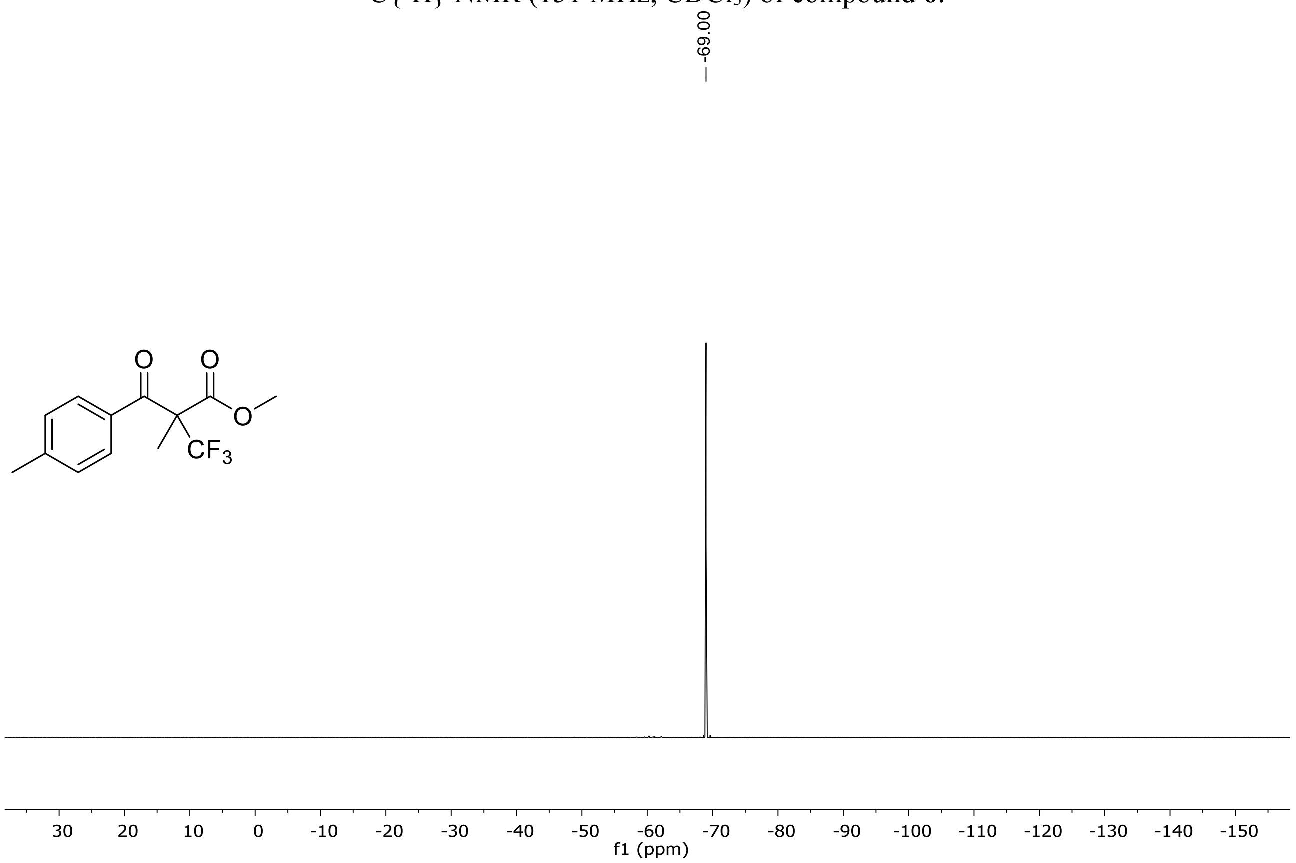

^{19}F NMR (282 MHz, $CDCl_3$) of compound **6**.

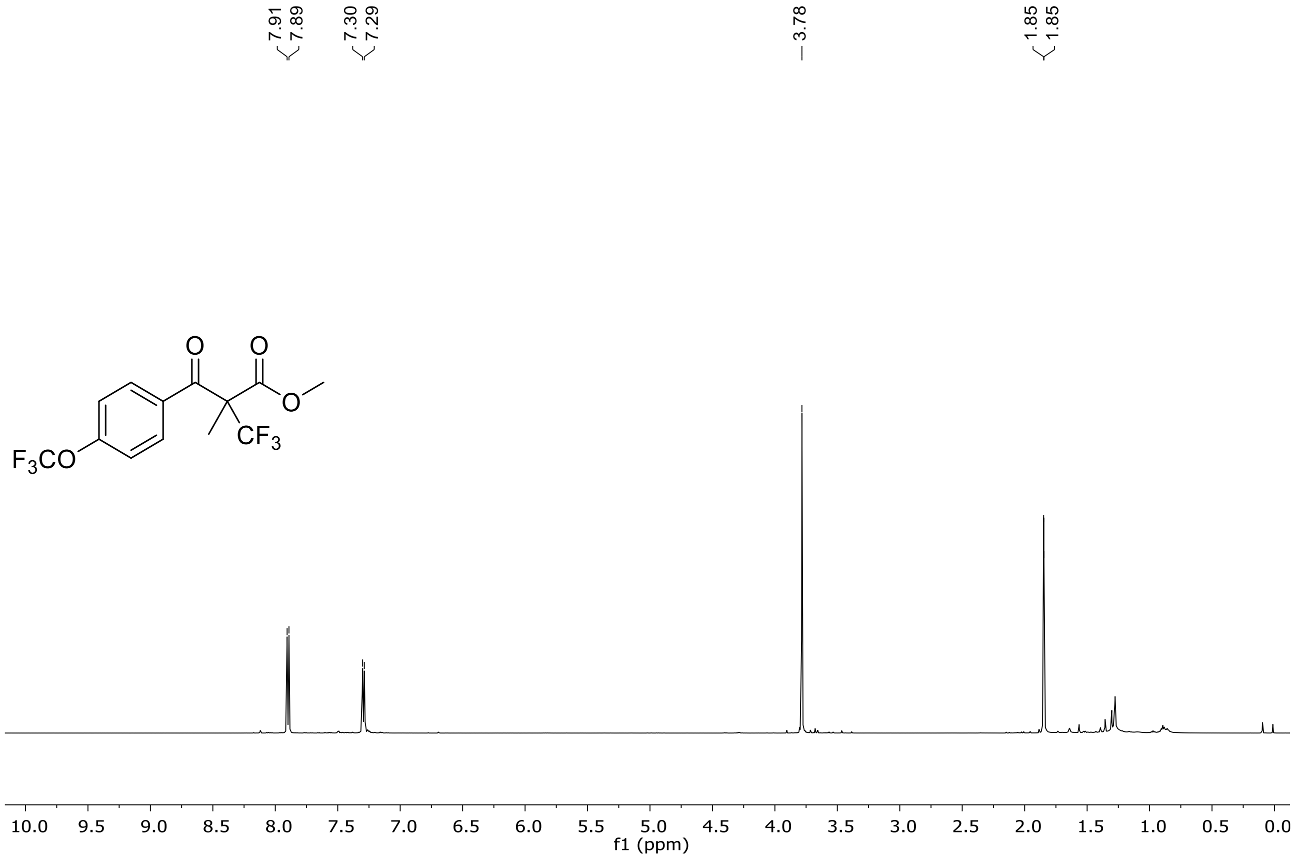

1H NMR (600 MHz, $CDCl_3$) of compound **7**.

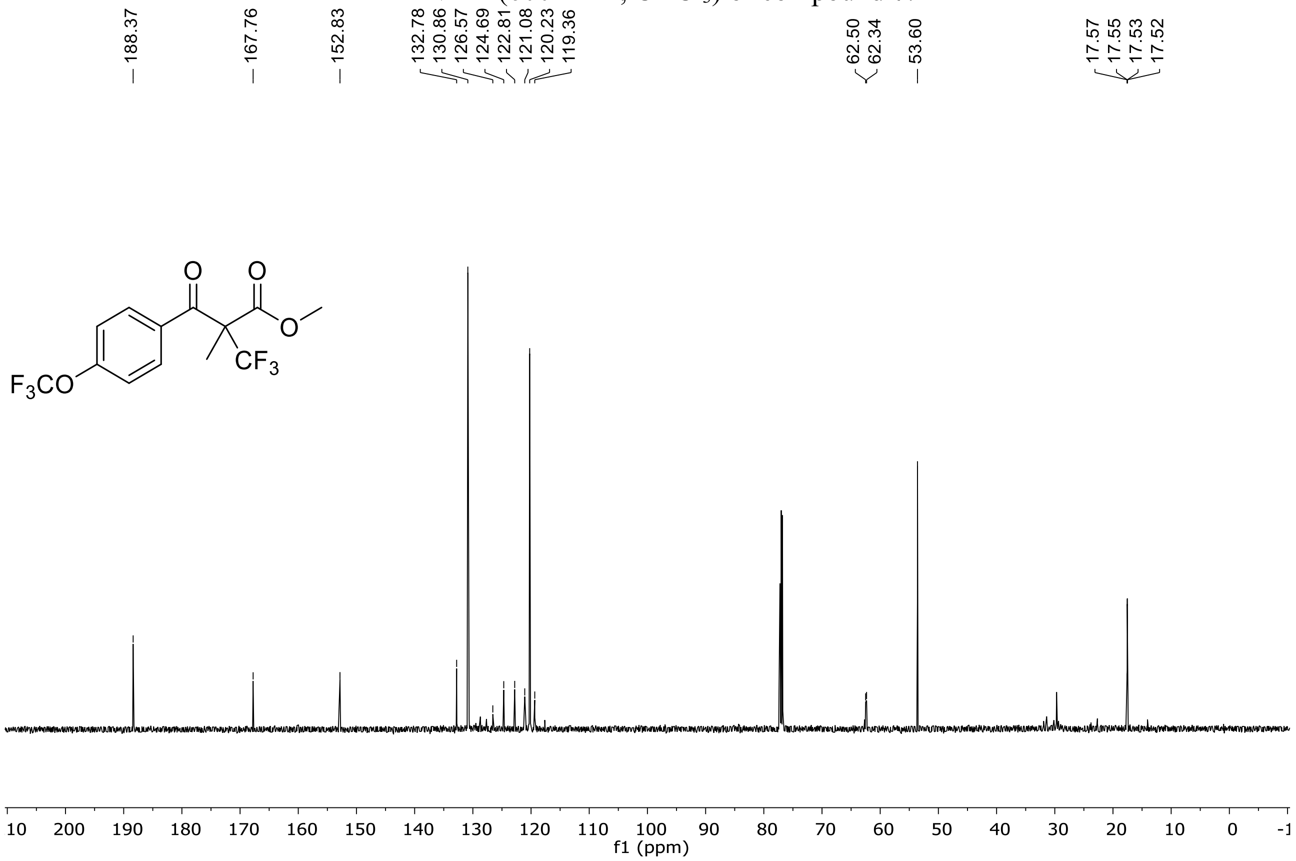

$^{13}C\{^{1}H\}$ NMR (151 MHz, $CDCl_3$) of compound **7**.

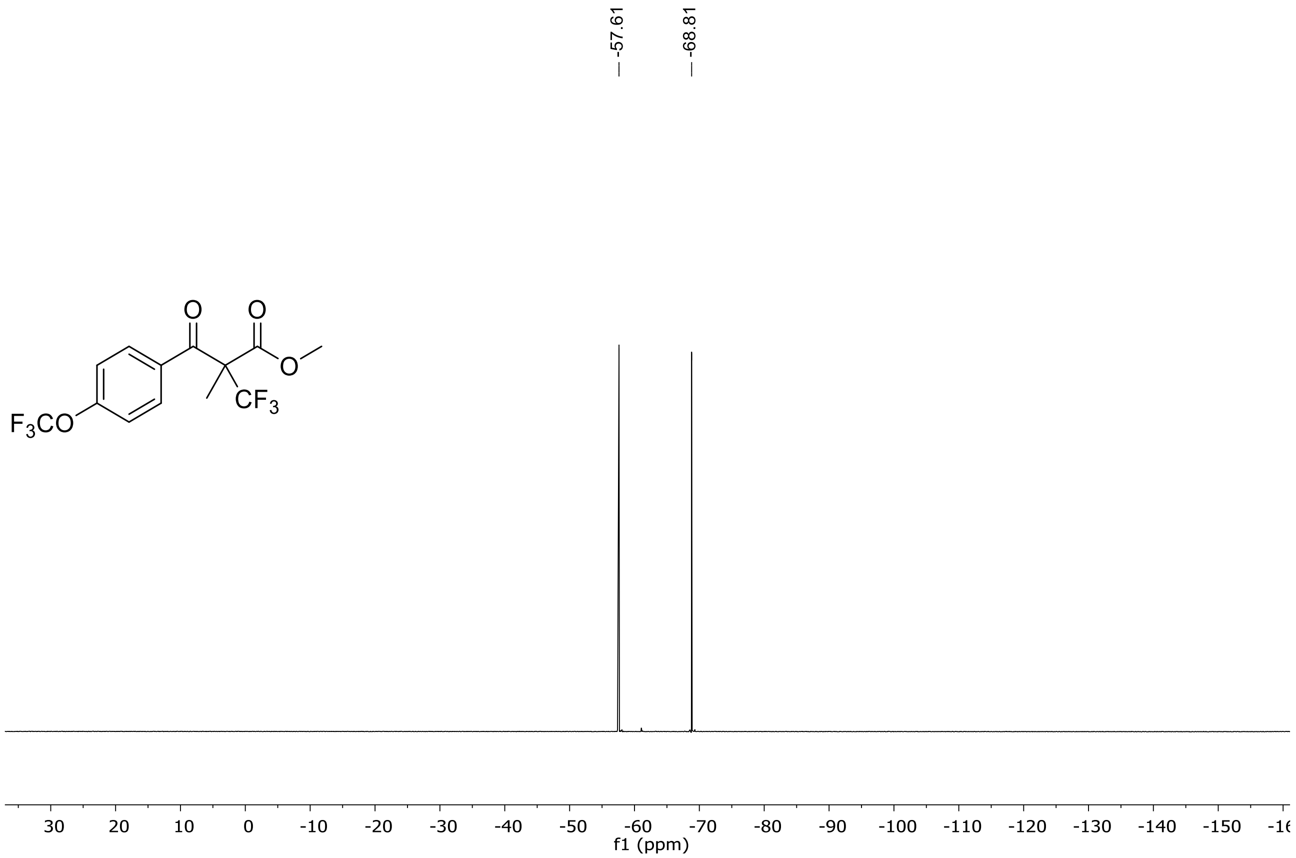

19F NMR (377 MHz, $CDCl_3$) of compound **7**.

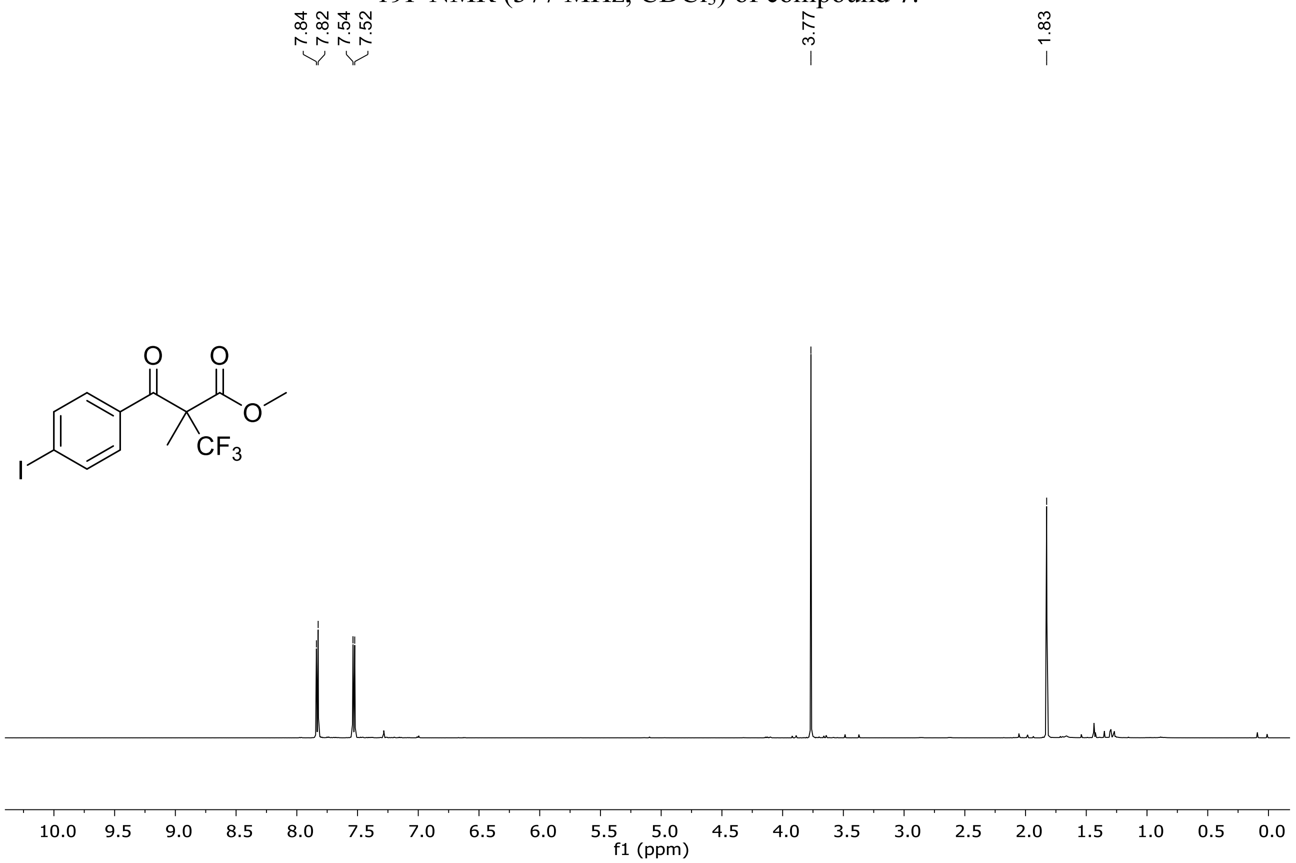

1H NMR (600 MHz, $CDCl_3$) of compound **8**.

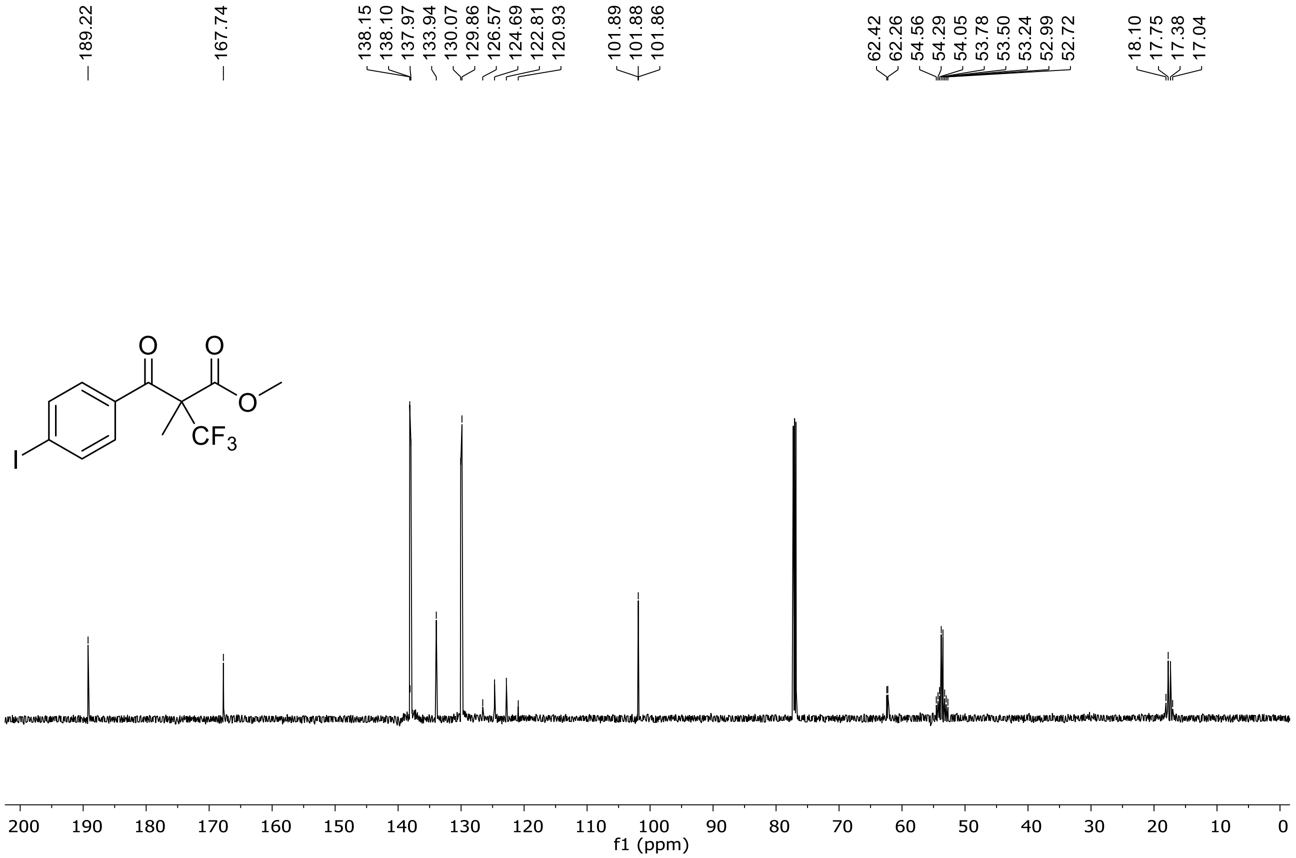

$^{13}C\{^{1}H\}$ NMR (151 MHz, $CDCl_3$) of compound **8**.

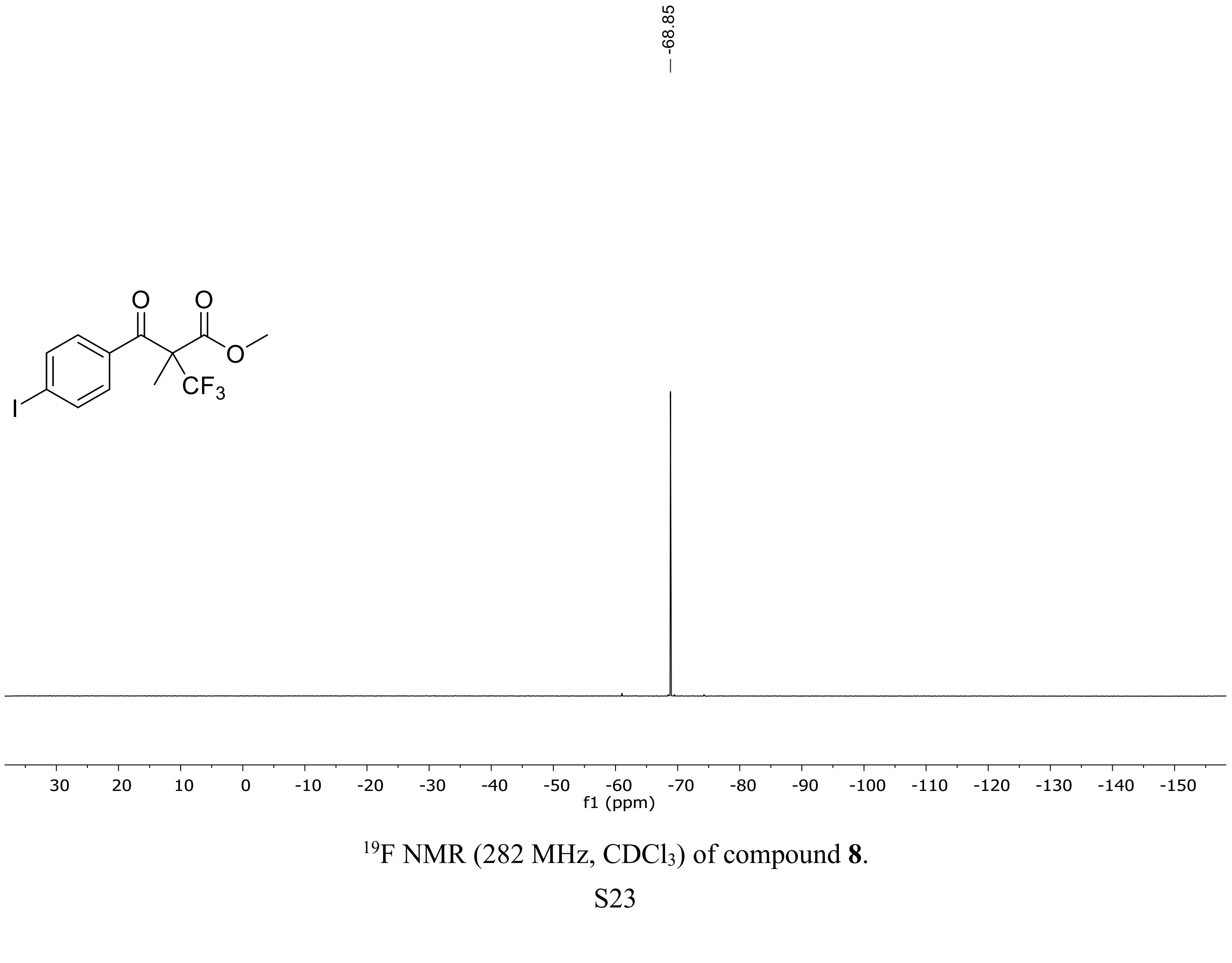

^{19}F NMR (282 MHz, $CDCl_3$) of compound **8**.

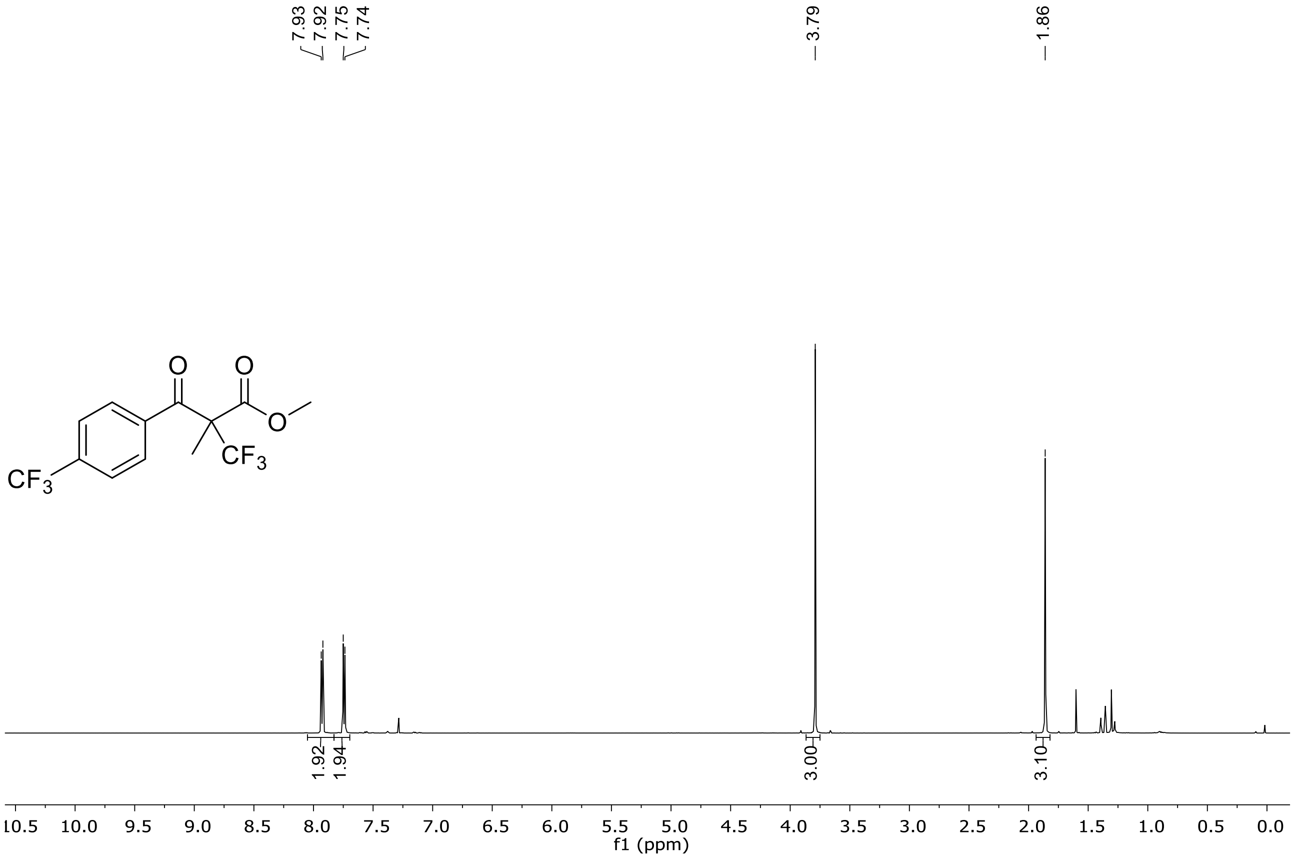

^{1}H NMR (600 MHz, $CDCl_3$) of compound **9**.

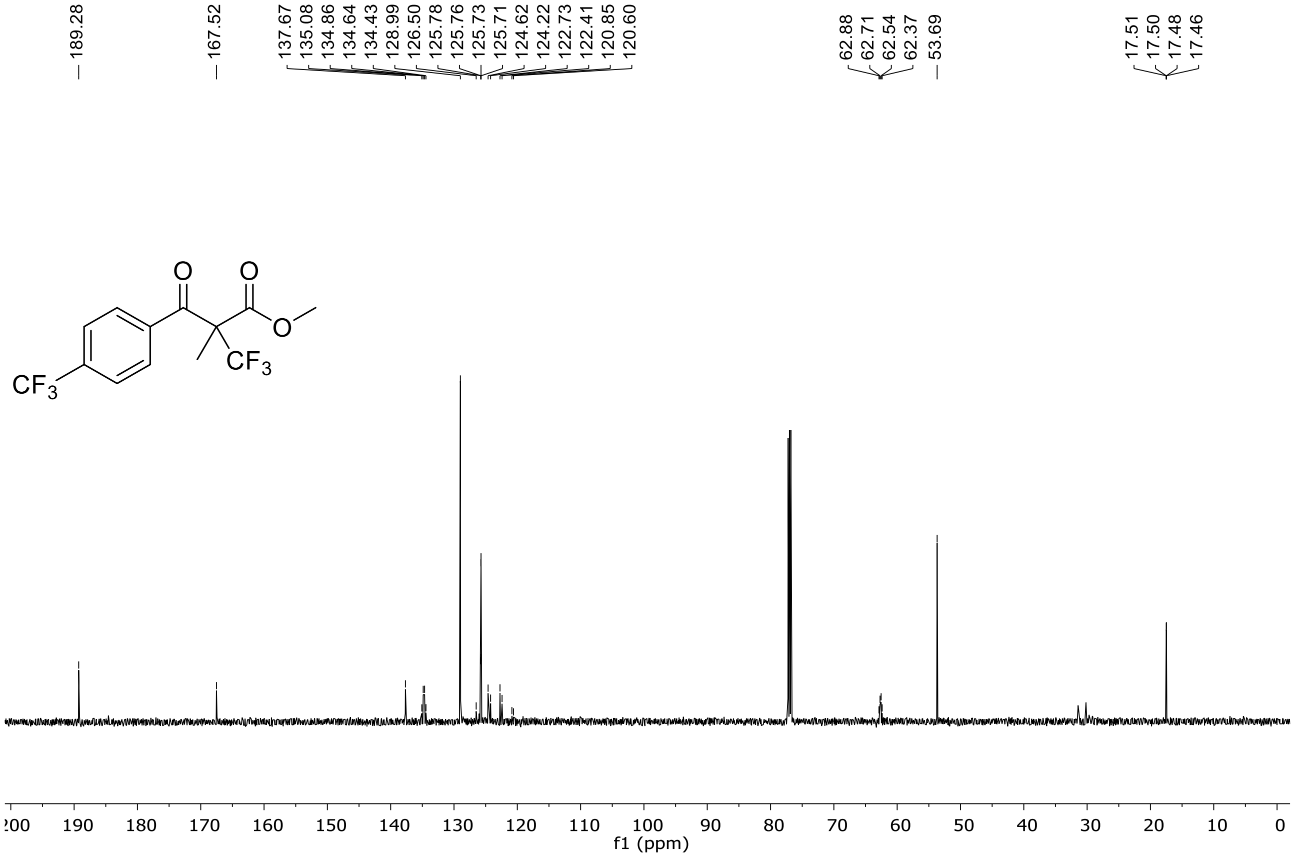

$^{13}C\{^{1}H\}$ NMR (151 MHz, $CDCl_3$) of compound **9**.

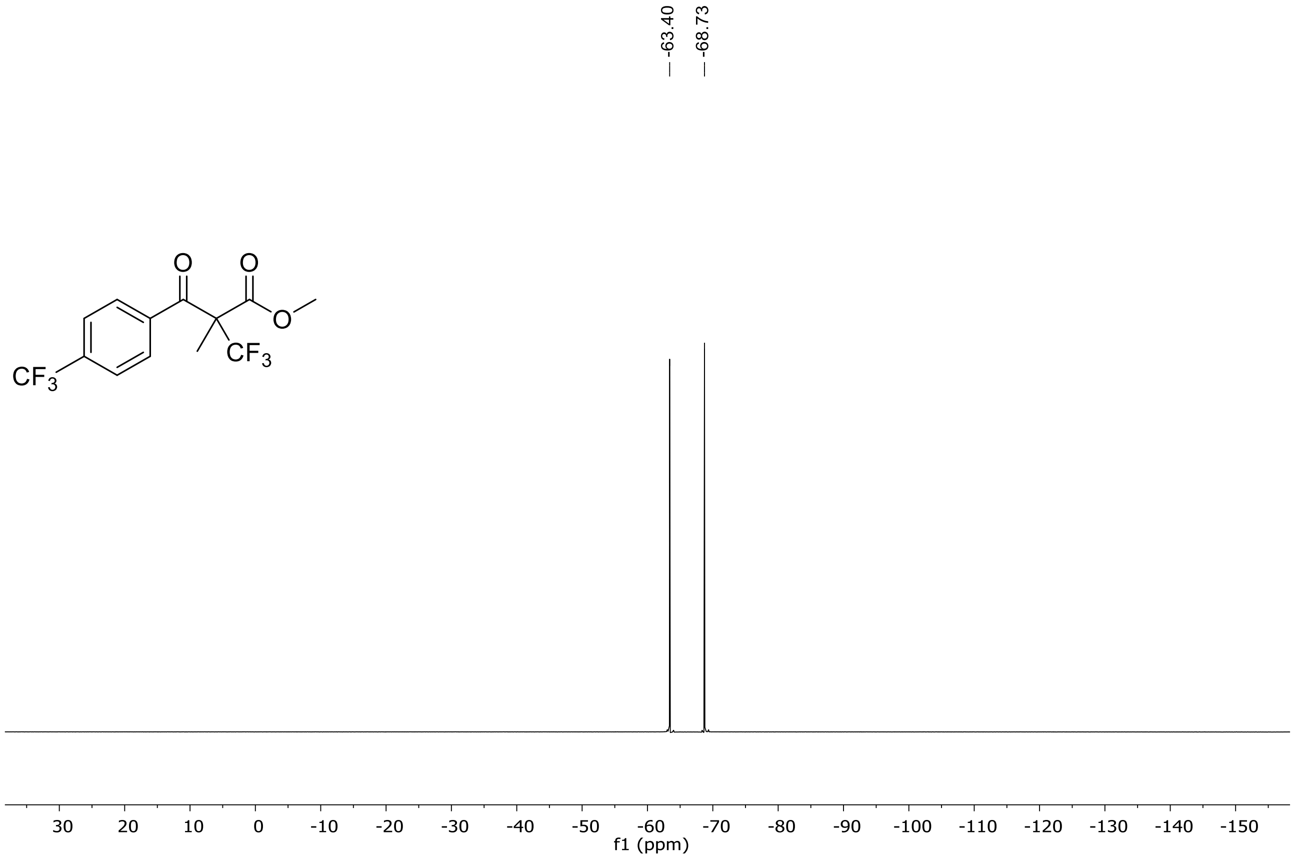

^{19}F NMR (282 MHz, $CDCl_3$) of compound **9**.

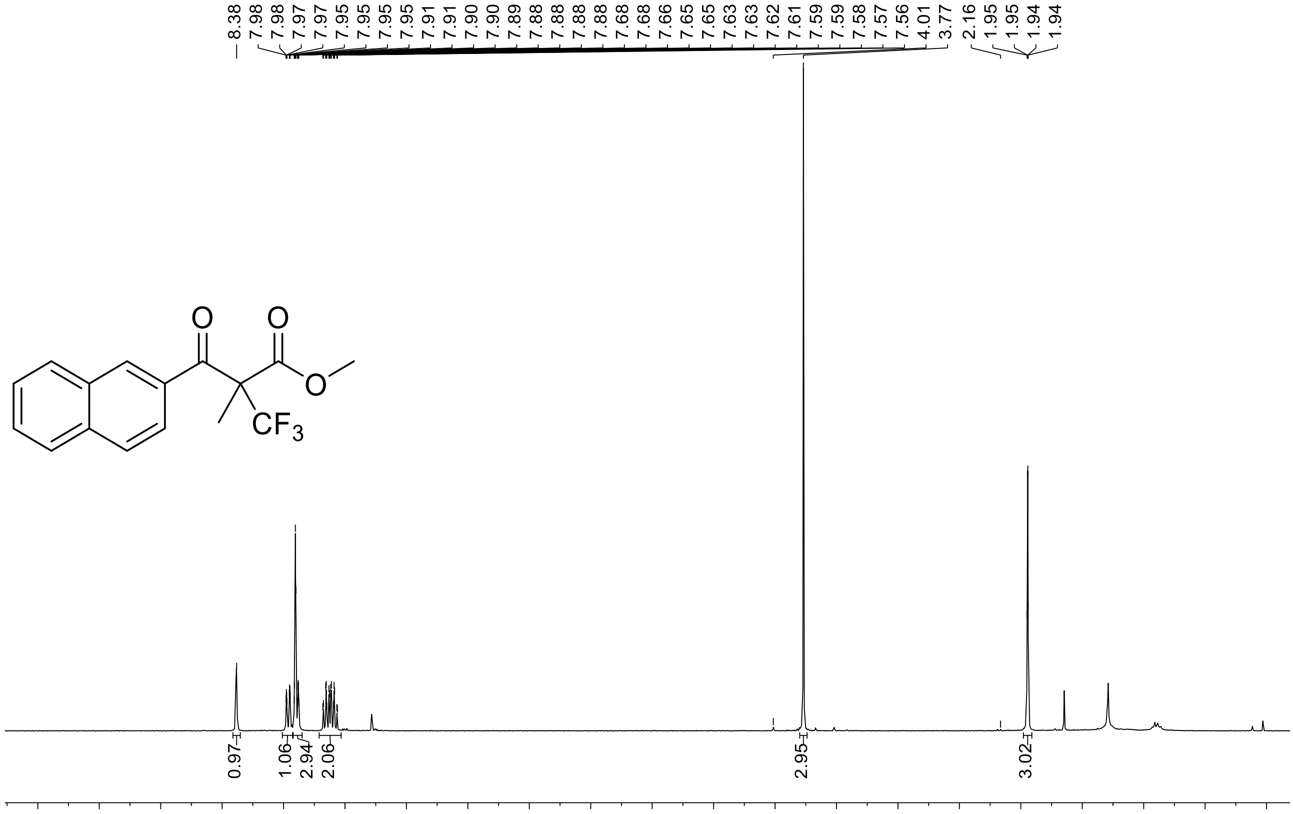

^{1}H NMR (300 MHz, $CDCl_3$) of compound **10**.

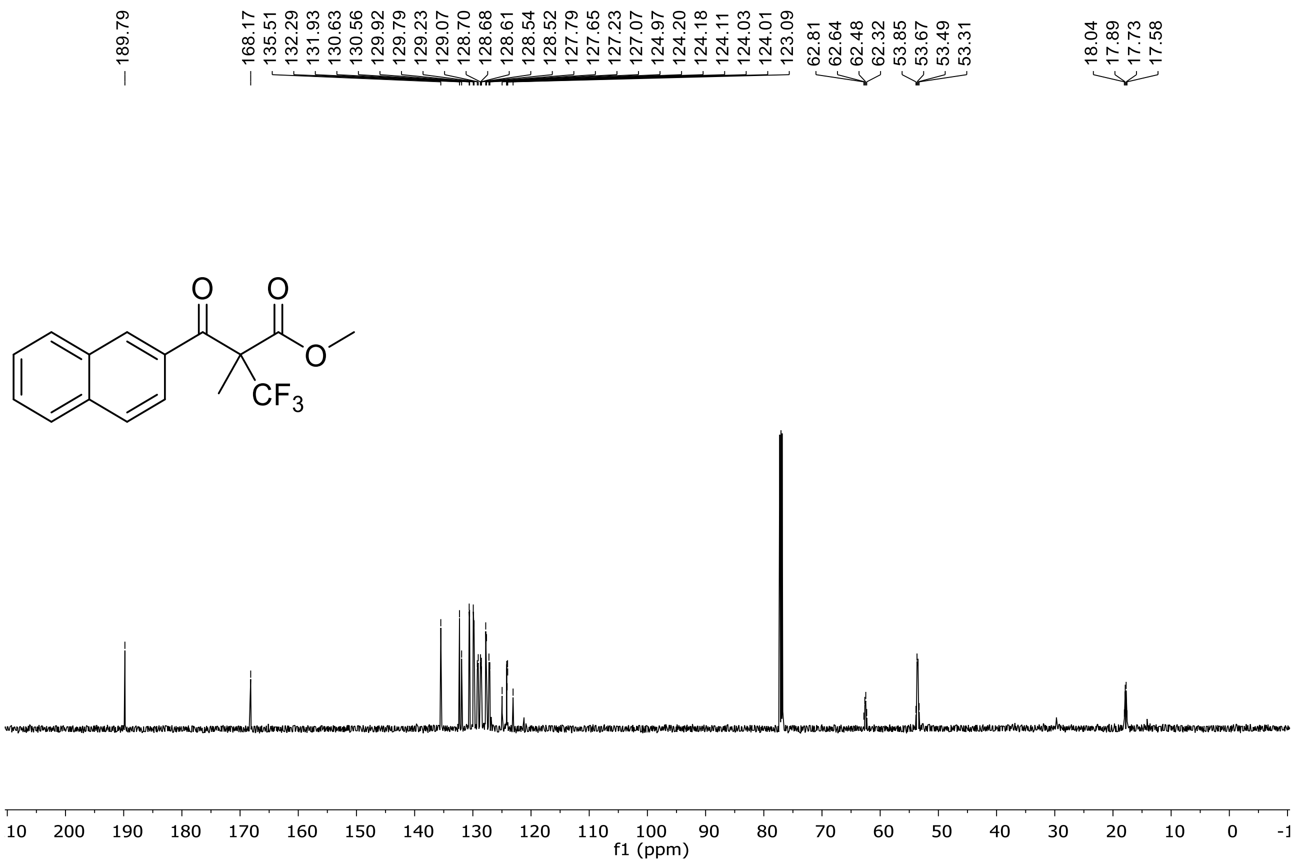

$^{13}C\{^{1}H\}$ NMR (151 MHz, $CDCl_3$) of compound **10**.

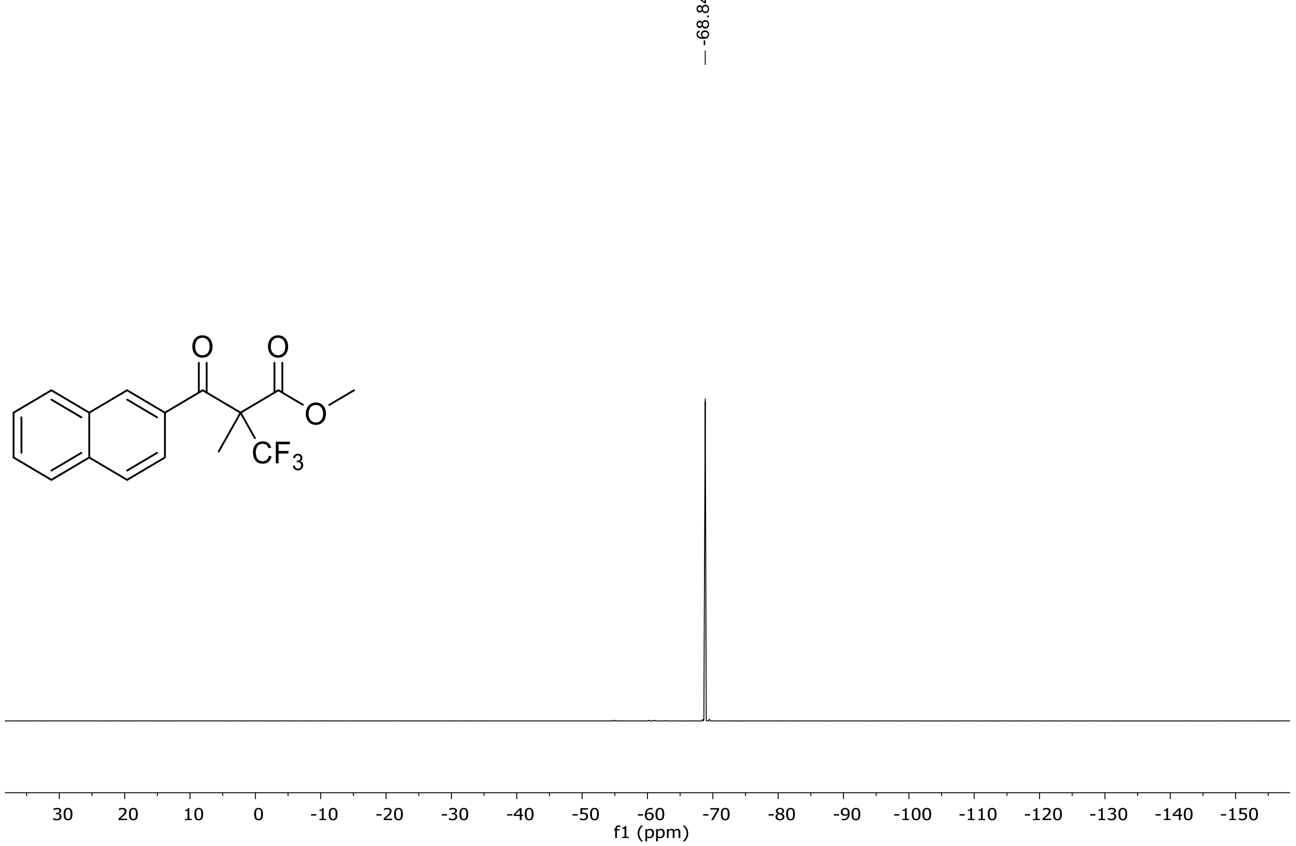

^{19}F NMR (282 MHz, $CDCl_3$) of compound **10**.

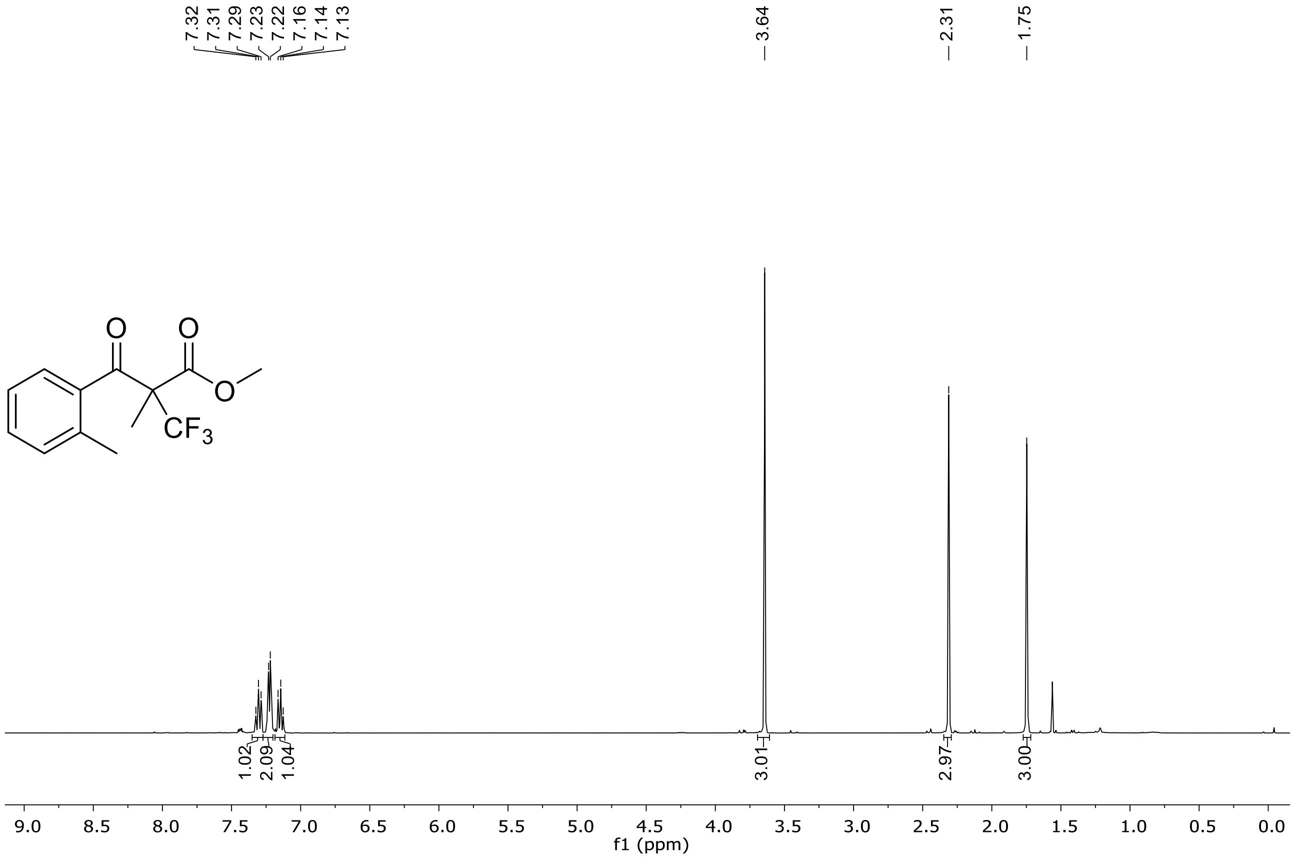

^{1}H NMR (400 MHz, $CDCl_3$) of compound **11**.

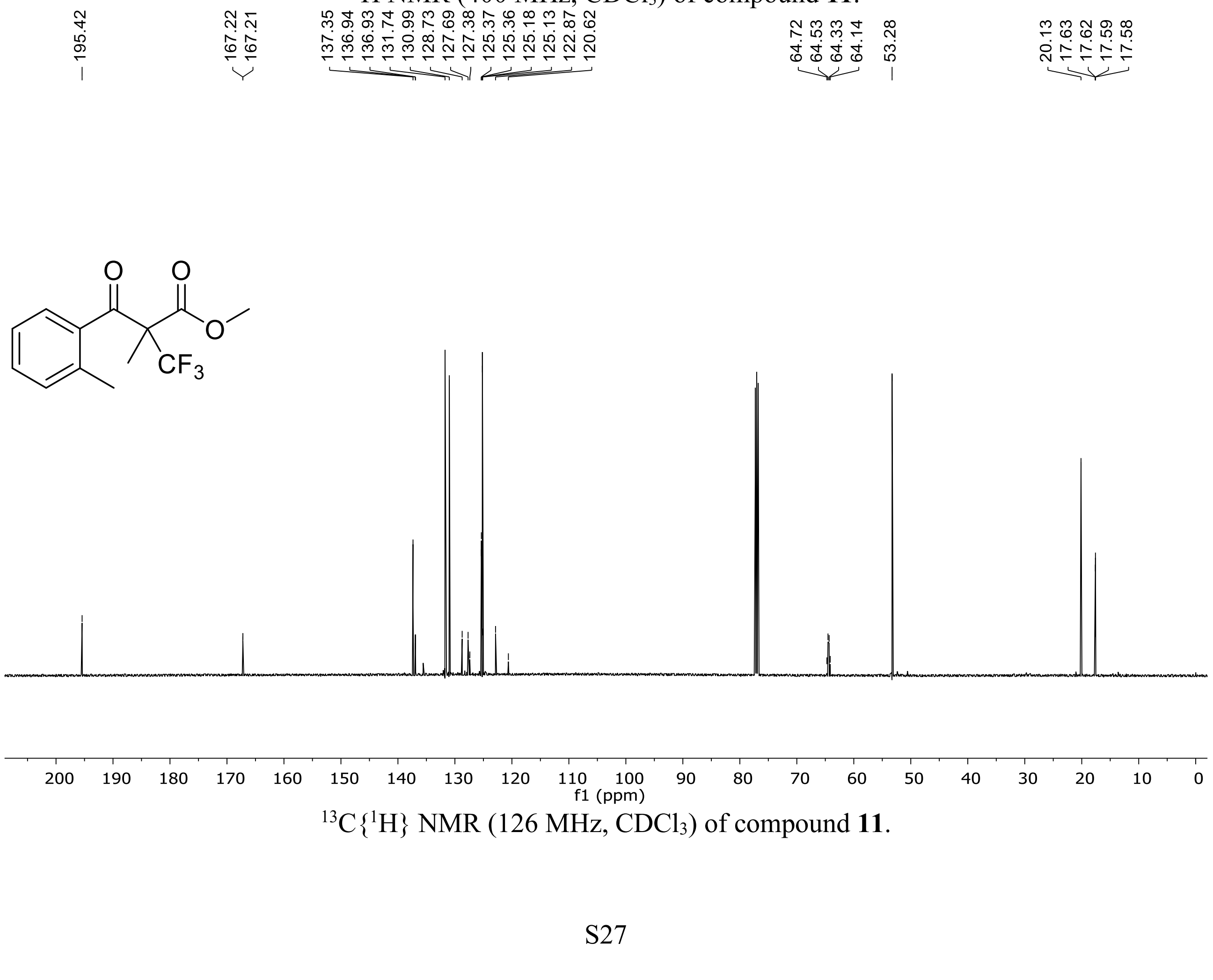

^{13}C{^{1}H} NMR (126 MHz, $CDCl_3$) of compound **11**.

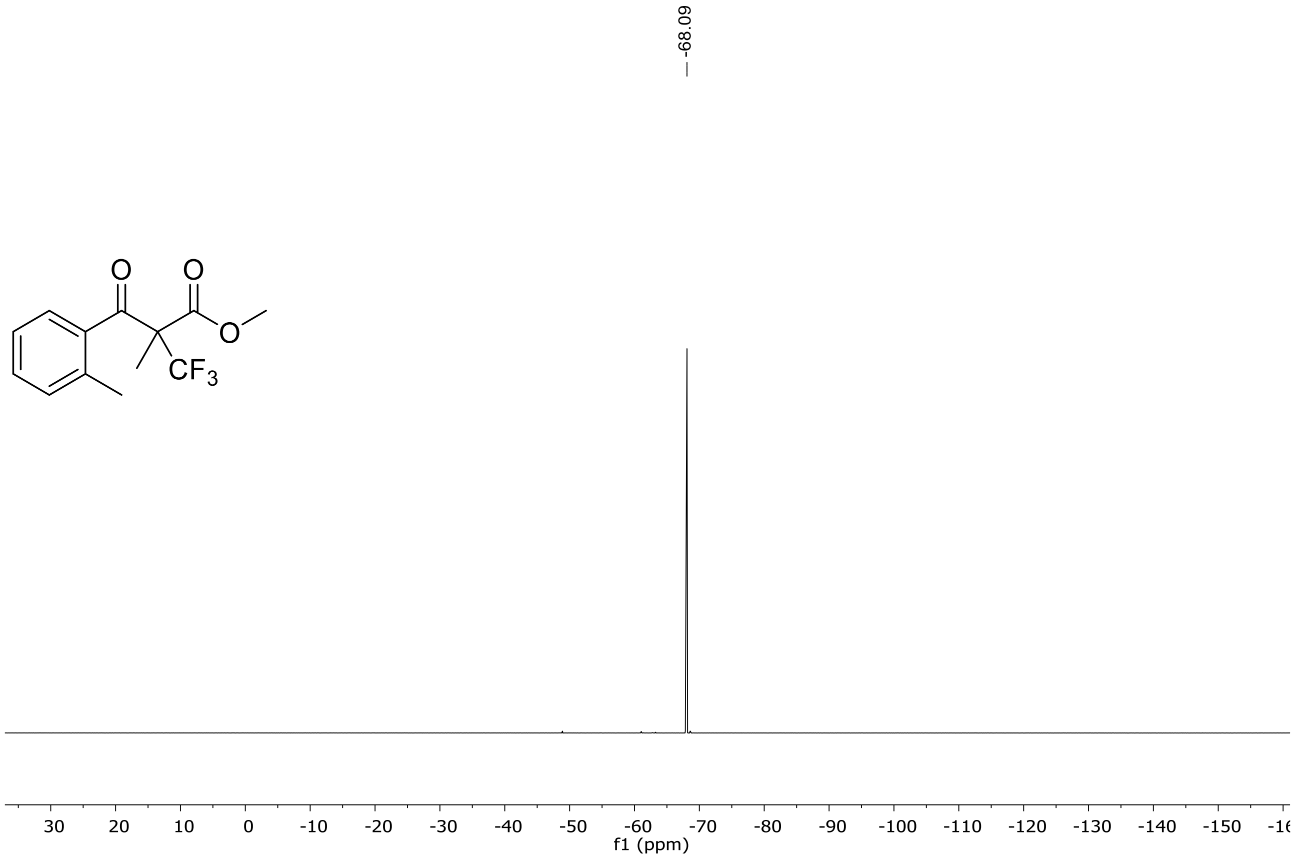

^{19}F NMR (377 MHz, $CDCl_3$) of compound **11**.

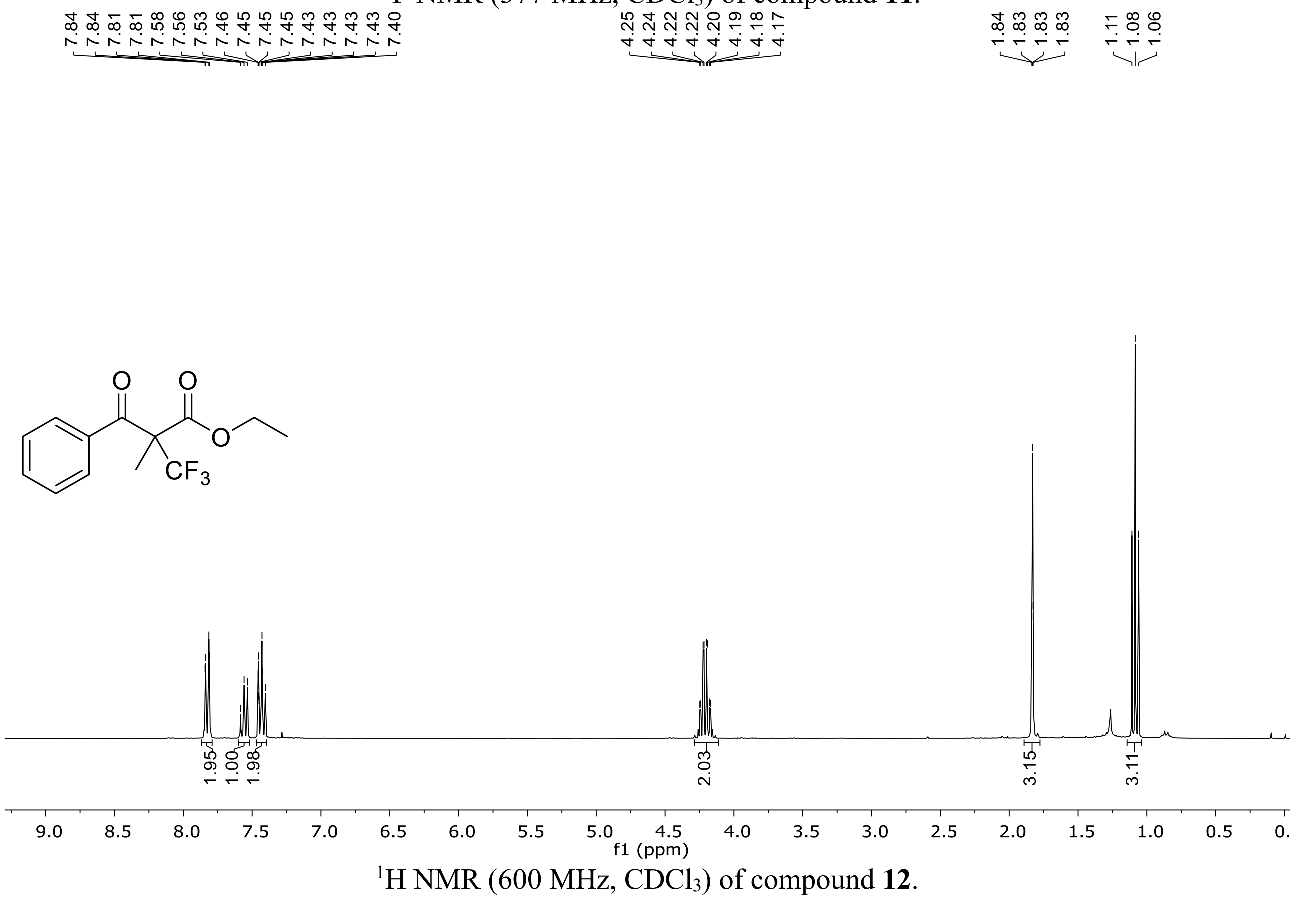

^{1}H NMR (600 MHz, $CDCl_3$) of compound **12**.

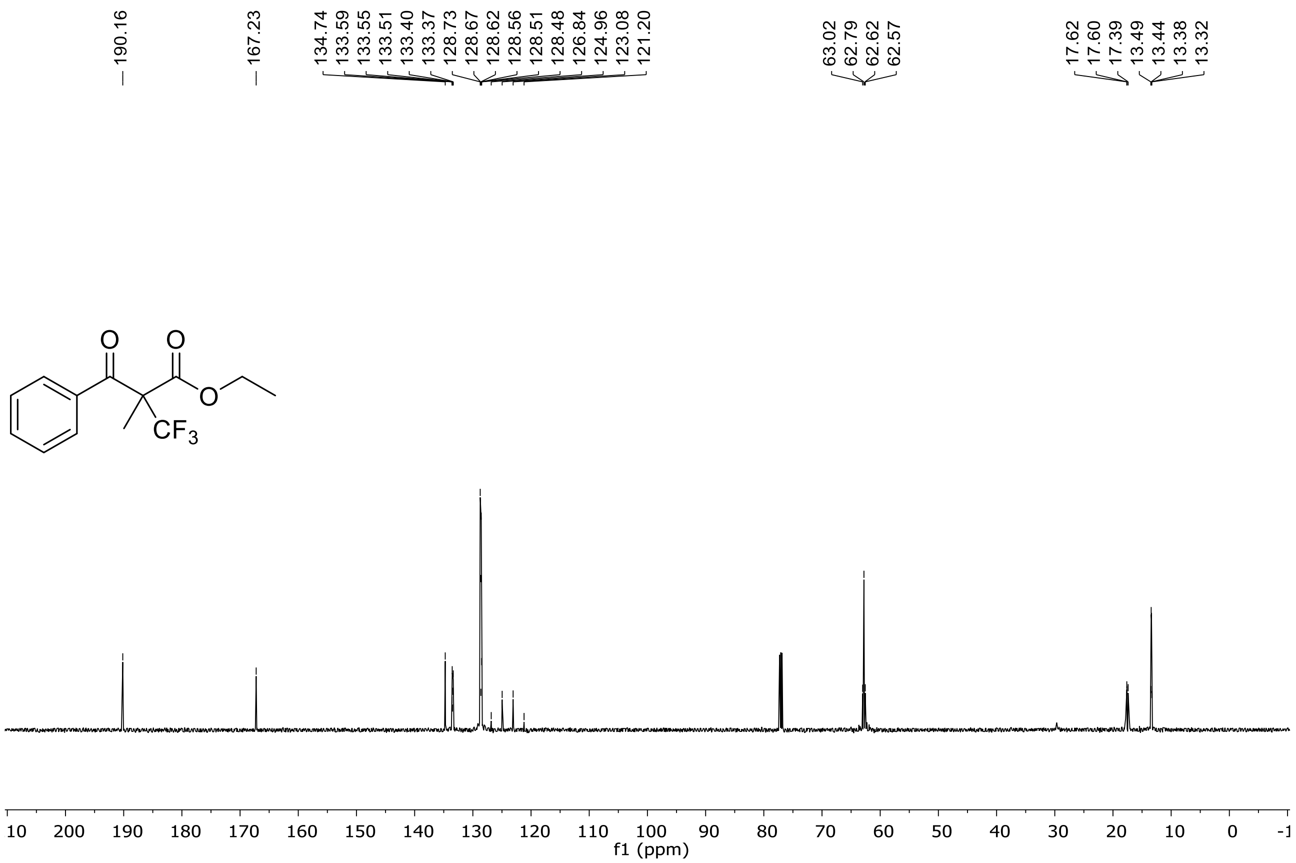

$^{13}C\{^{1}H\}$ NMR (151 MHz, $CDCl_3$) of compound **12**.

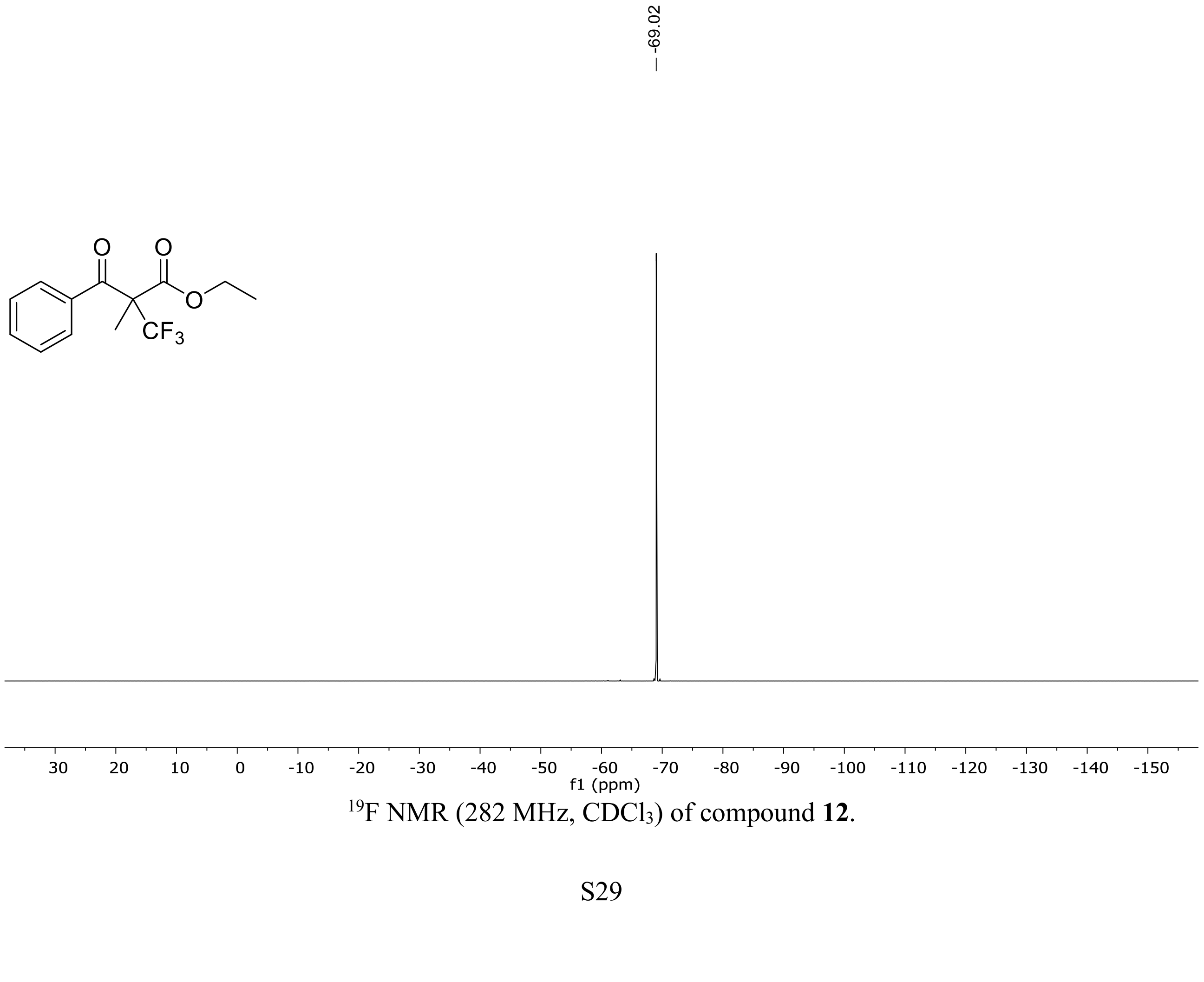

^{19}F NMR (282 MHz, $CDCl_3$) of compound **12**.

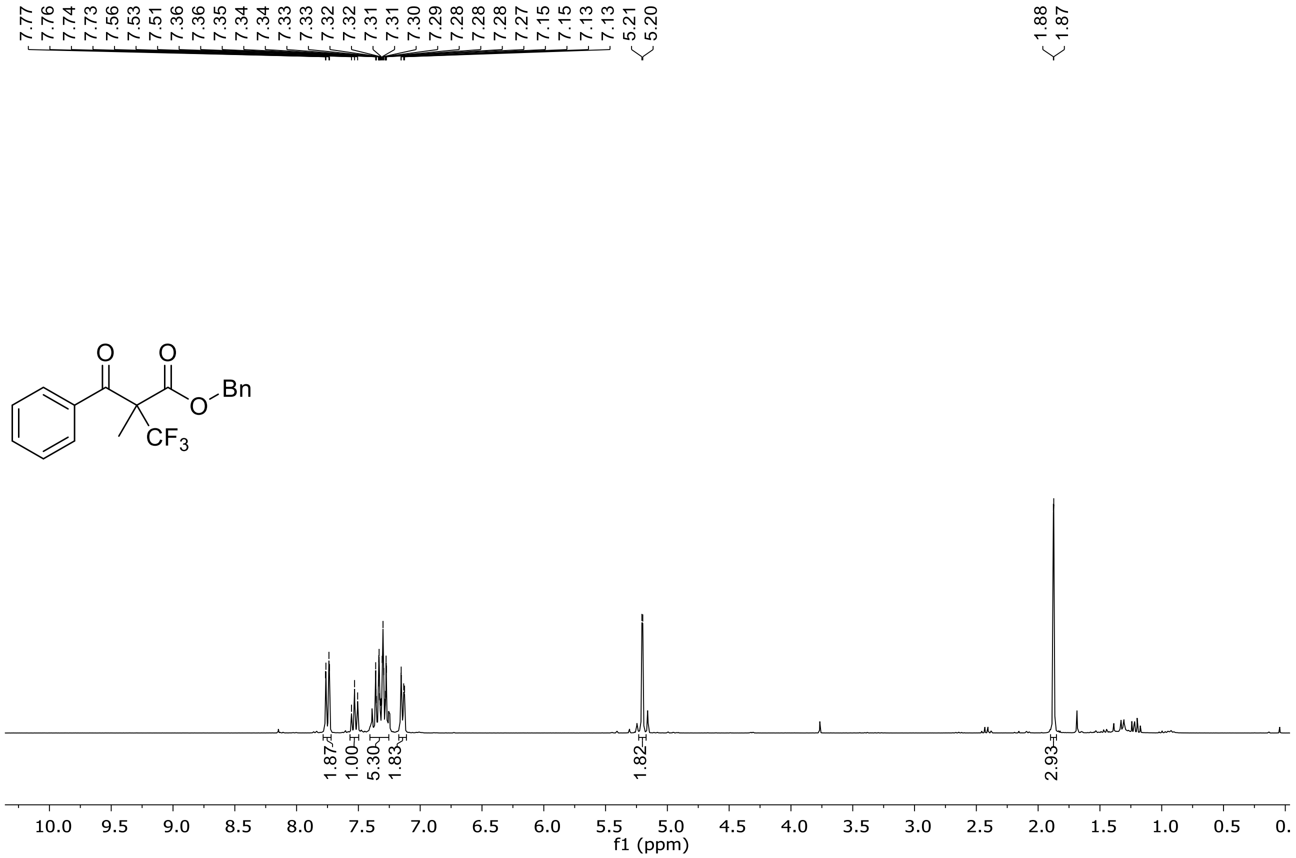

^{1}H NMR (300 MHz, $CDCl_3$) of compound **13**.

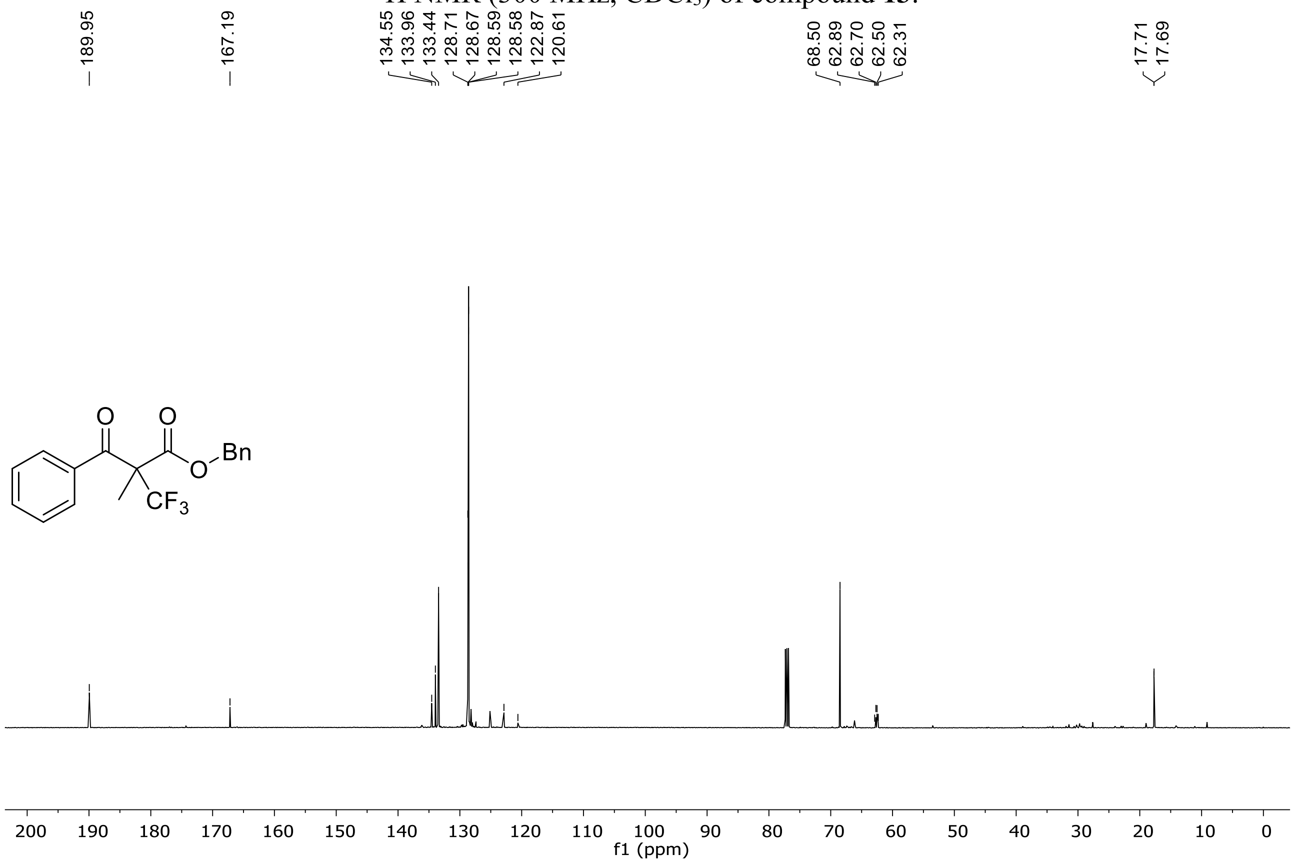

^{13}C{^{1}H} NMR (126 MHz, $CDCl_3$) of compound **13**.

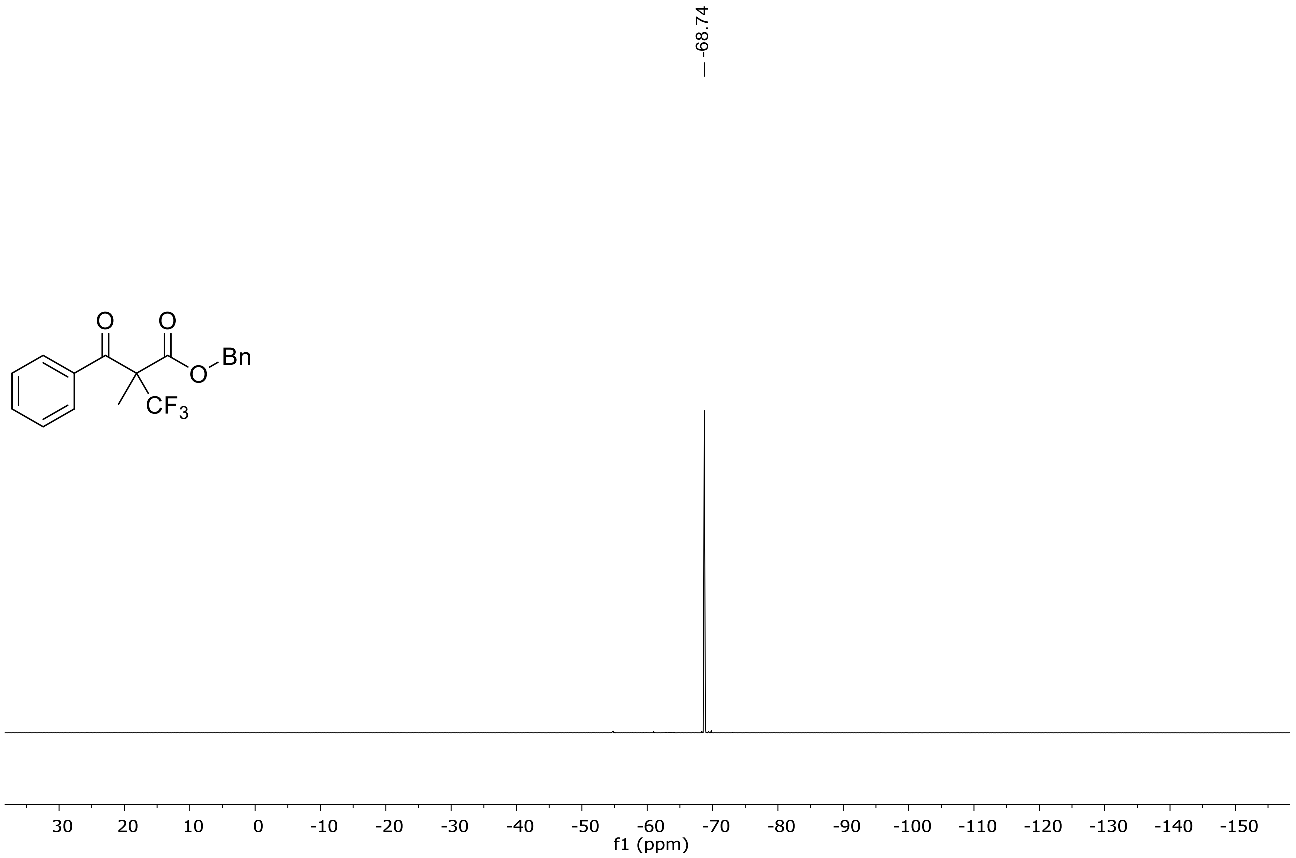

^{19}F NMR (282 MHz, $CDCl_3$) of compound **13**.

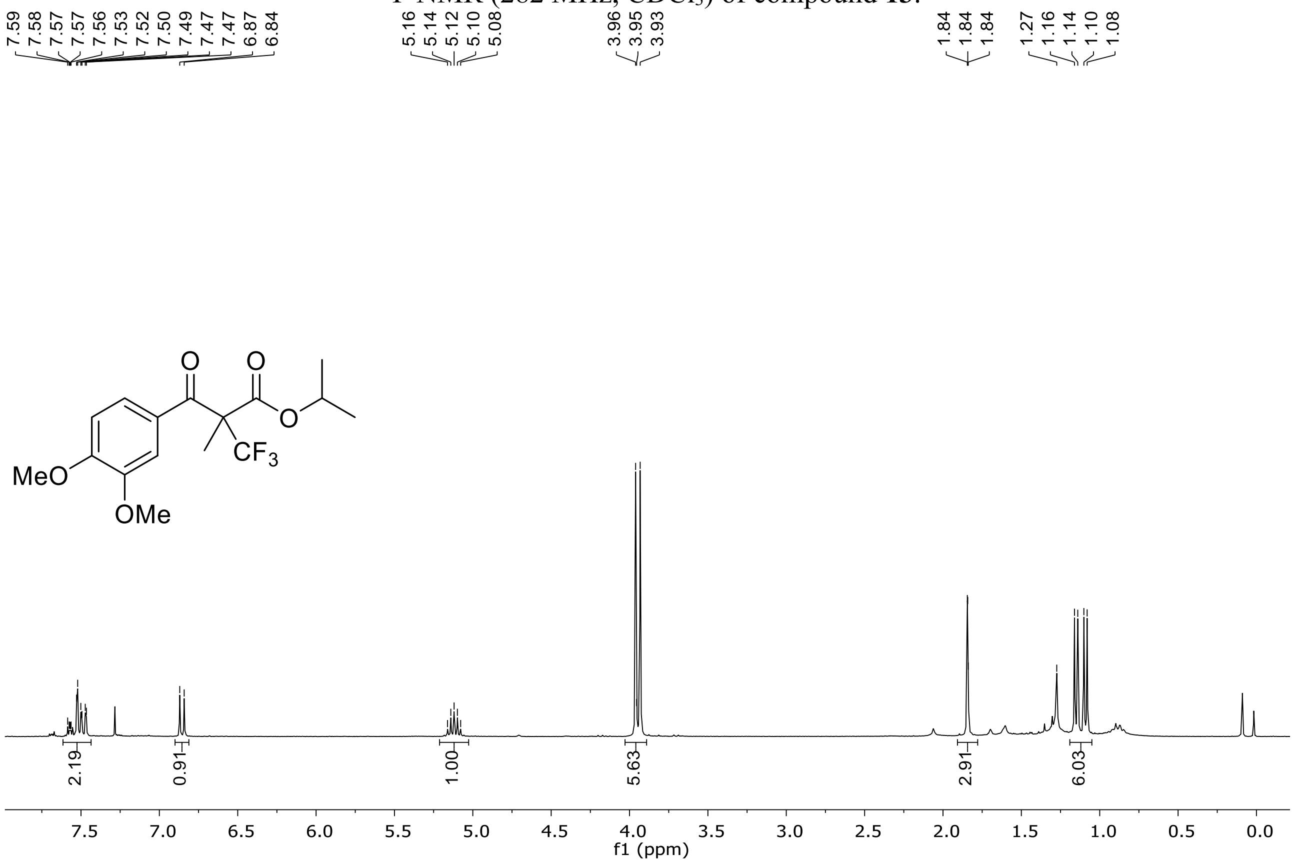

^{1}H NMR (300 MHz, $CDCl_3$) of compound **14**.

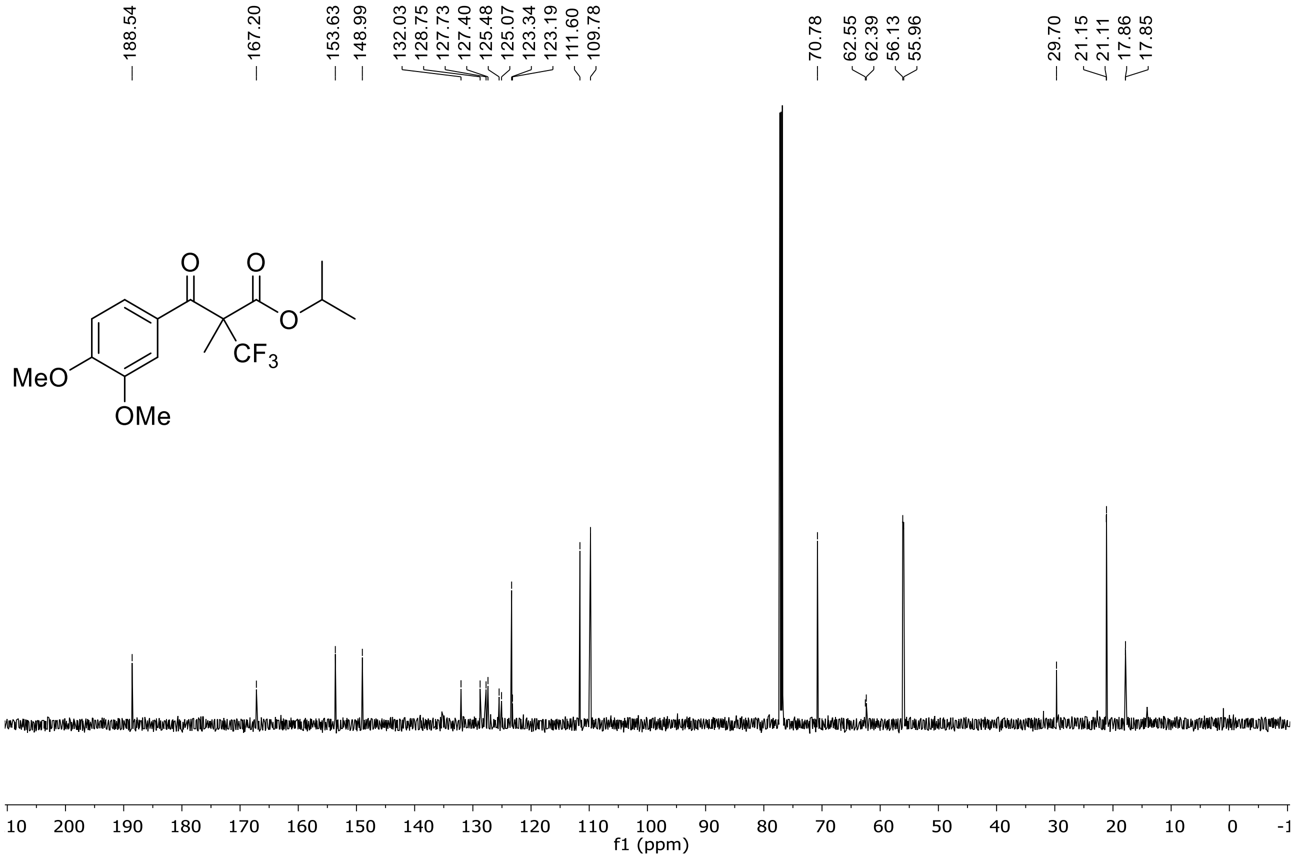

$^{13}C\{^1H\}$ NMR (151 MHz, $CDCl_3$) of compound **14**.

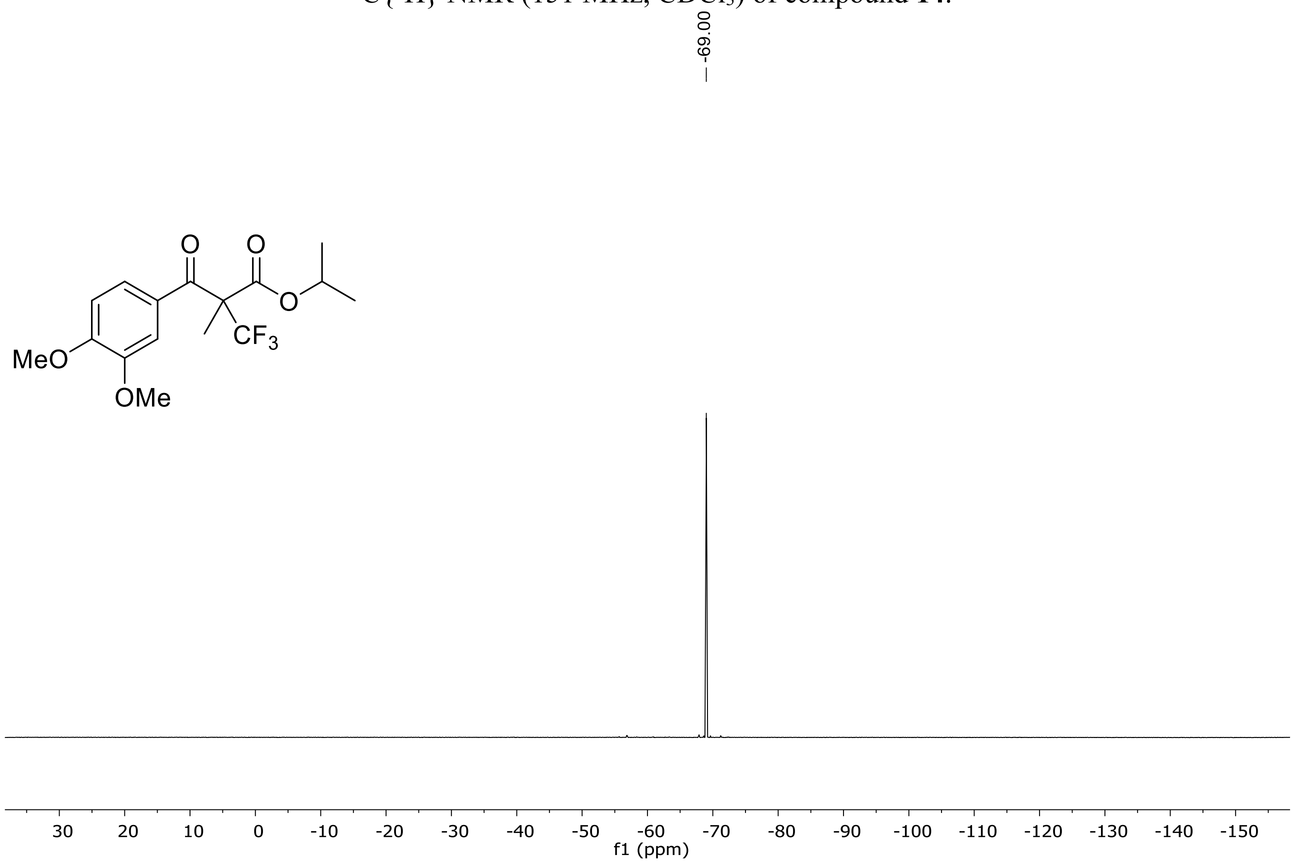

^{19}F NMR (282 MHz, $CDCl_3$) of compound **14**.

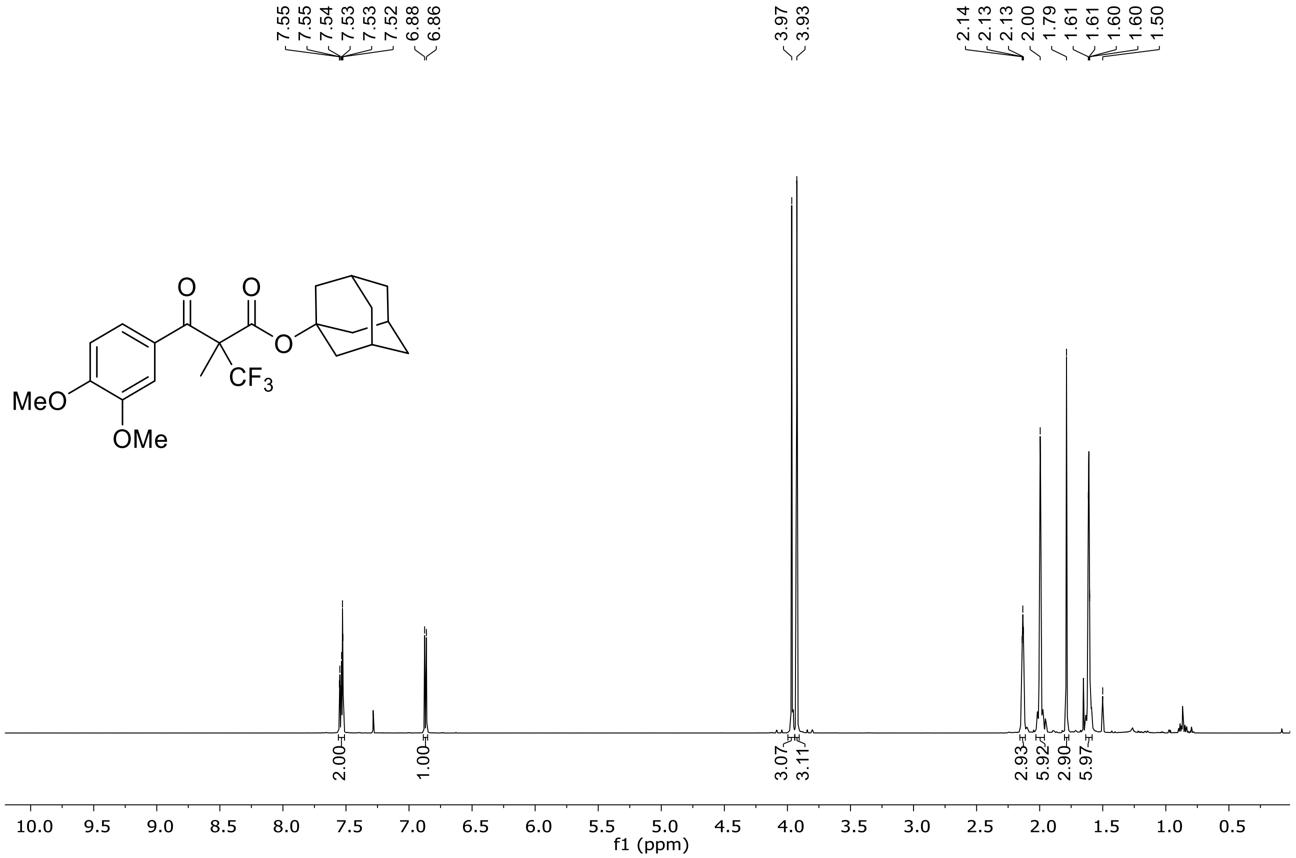

^{1}H NMR (600 MHz, CDCl$_3$) of compound **15**.

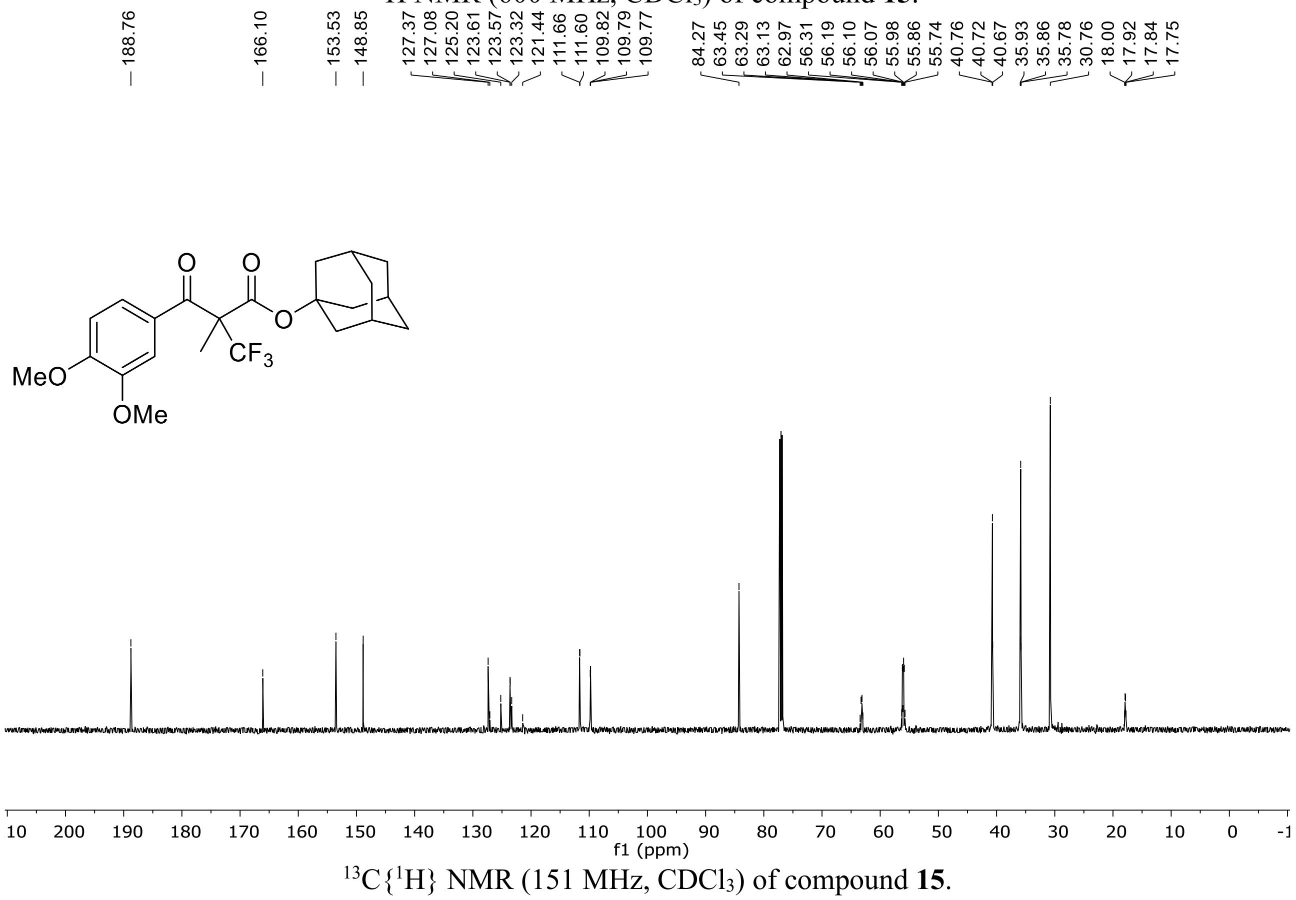

^{13}C{^{1}H} NMR (151 MHz, CDCl$_3$) of compound **15**.

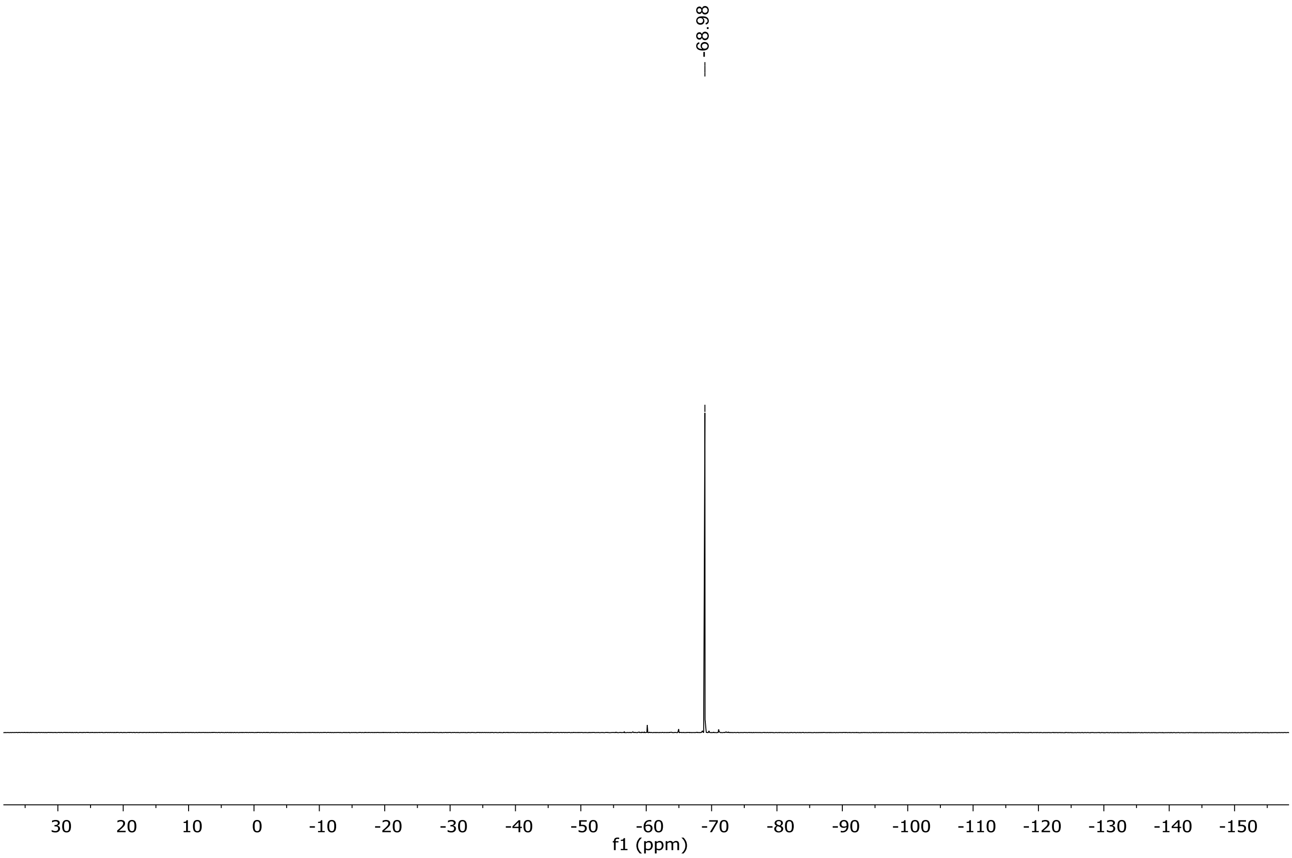

^{19}F NMR (282 MHz, $CDCl_3$) of compound **15**.

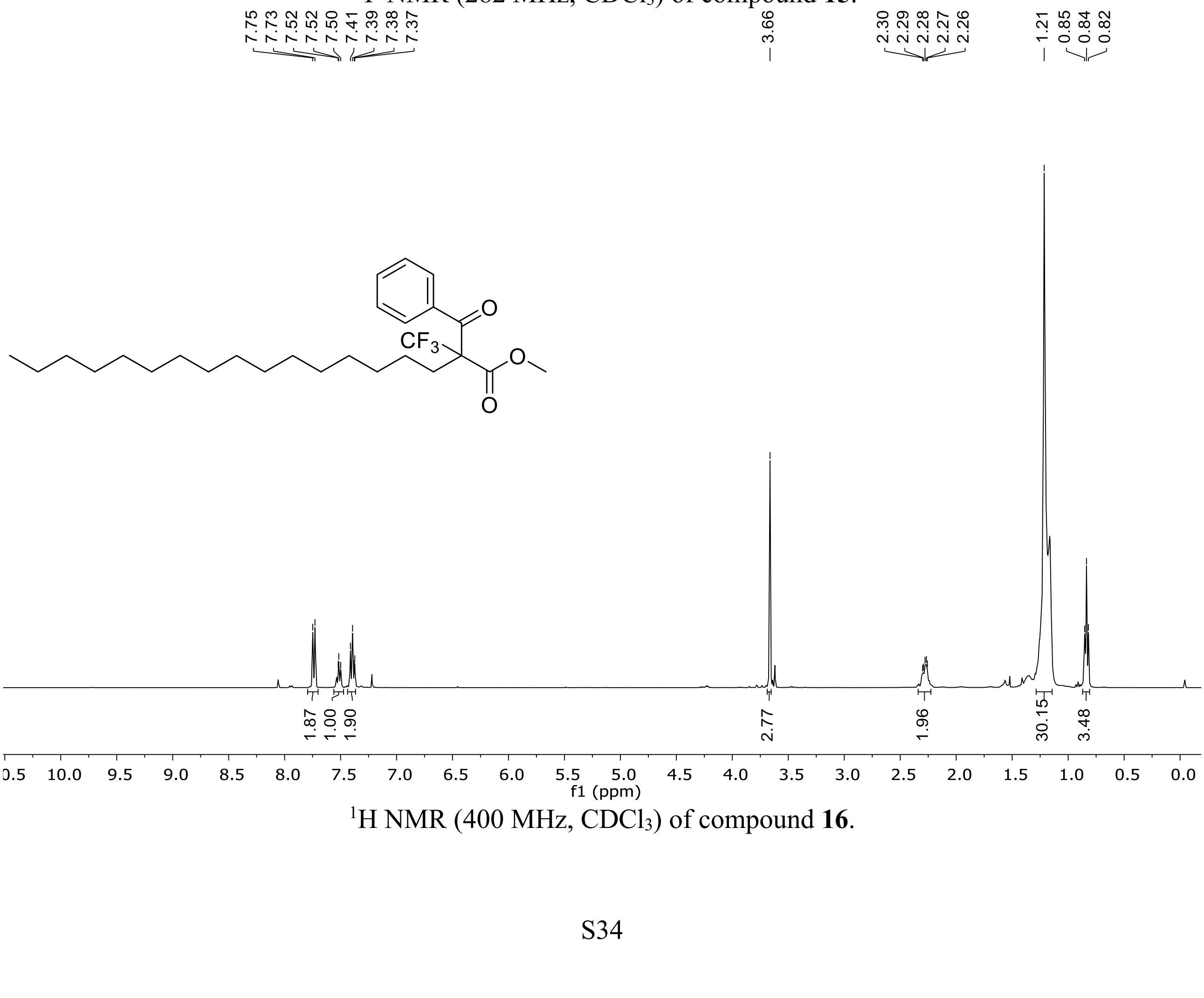

^{1}H NMR (400 MHz, $CDCl_3$) of compound **16**.

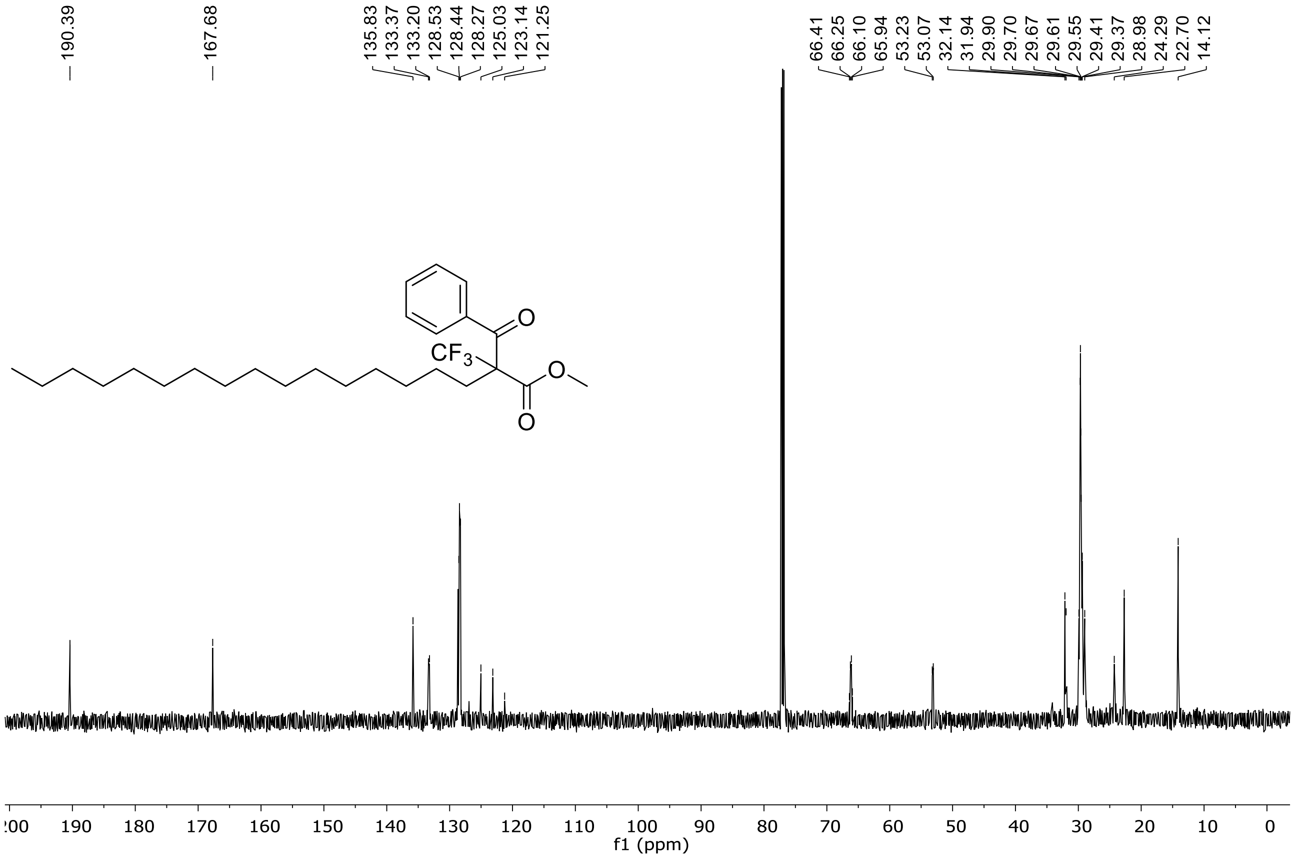

$^{13}C\{^{1}H\}$ NMR (151 MHz, $CDCl_3$) of compound **16**.

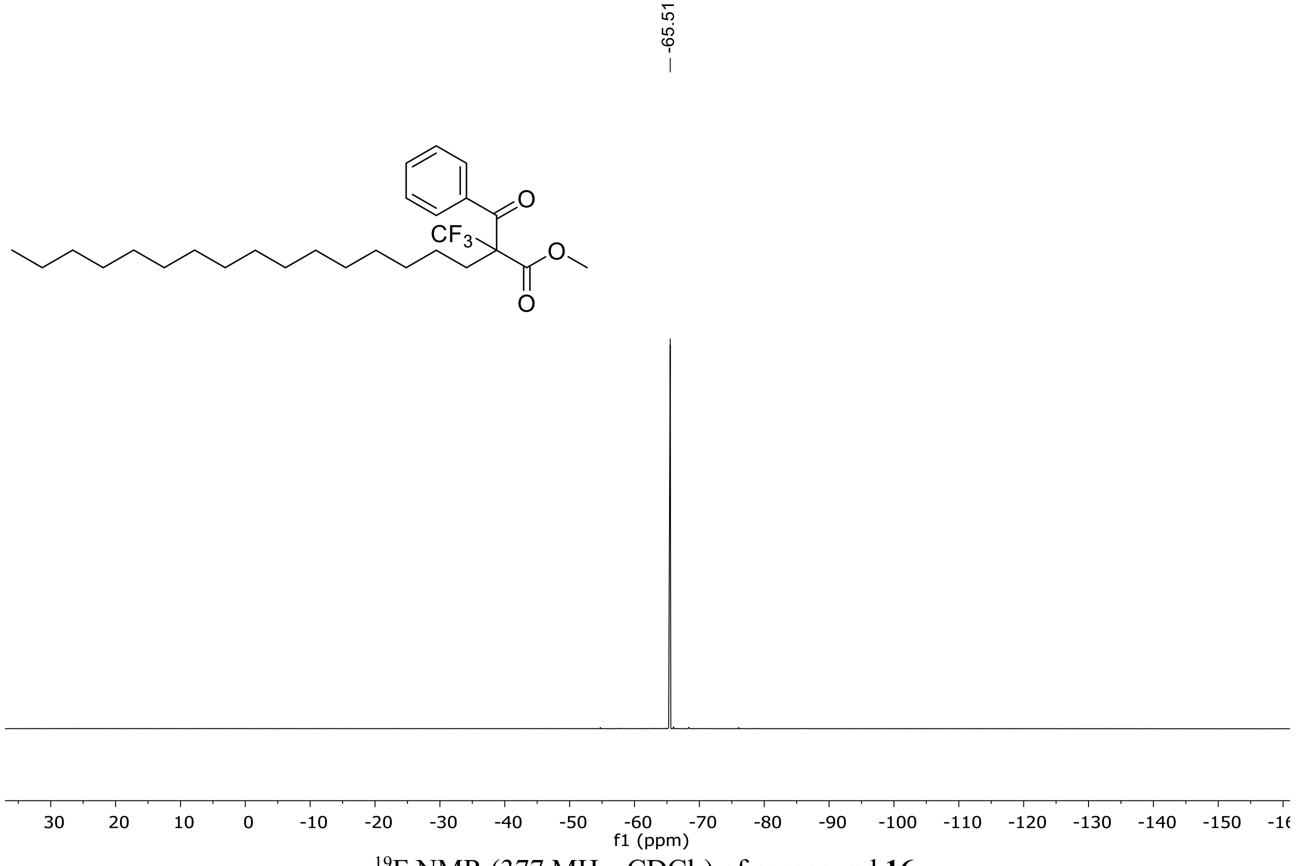

^{19}F NMR (377 MHz, $CDCl_3$) of compound **16**.

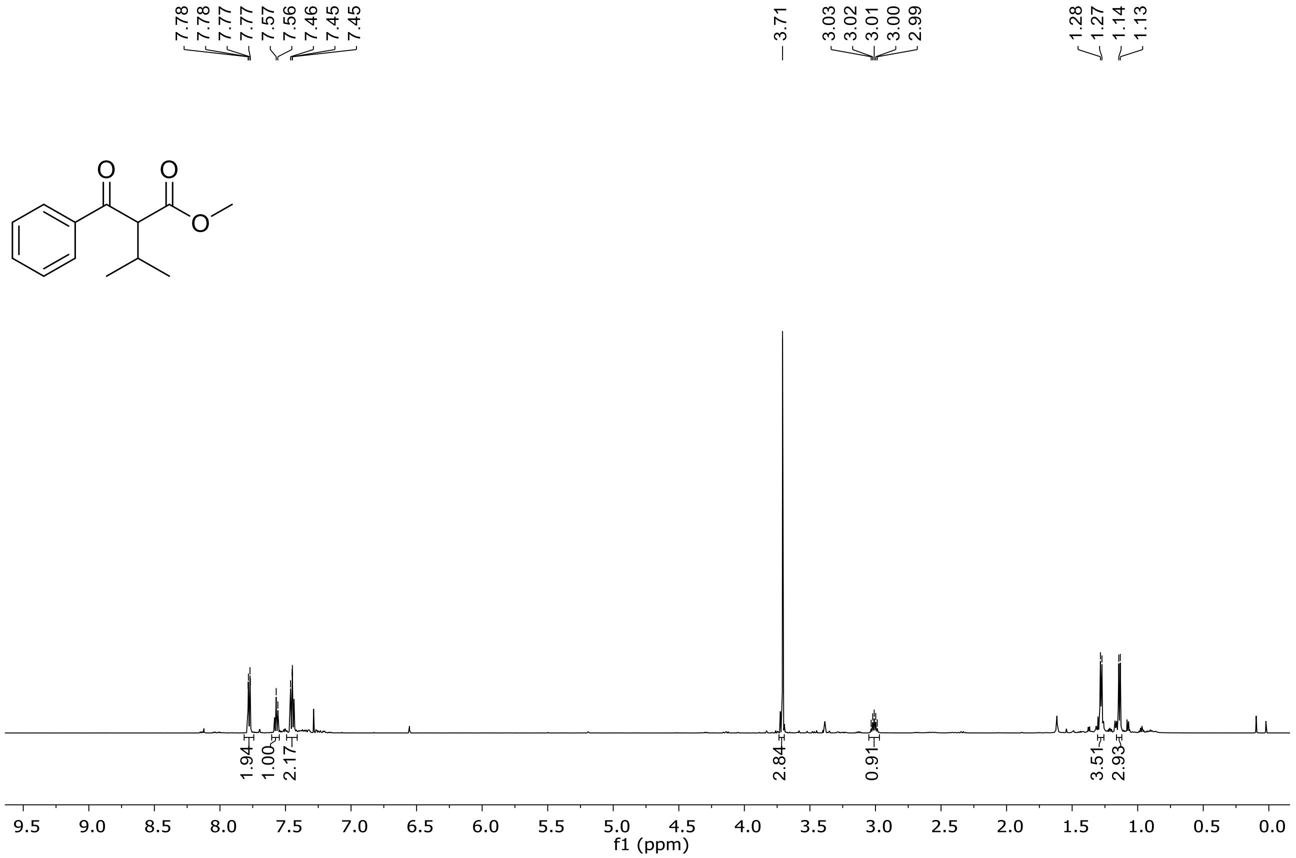

^{1}H NMR (600 MHz, $CDCl_3$) of compound **17**.

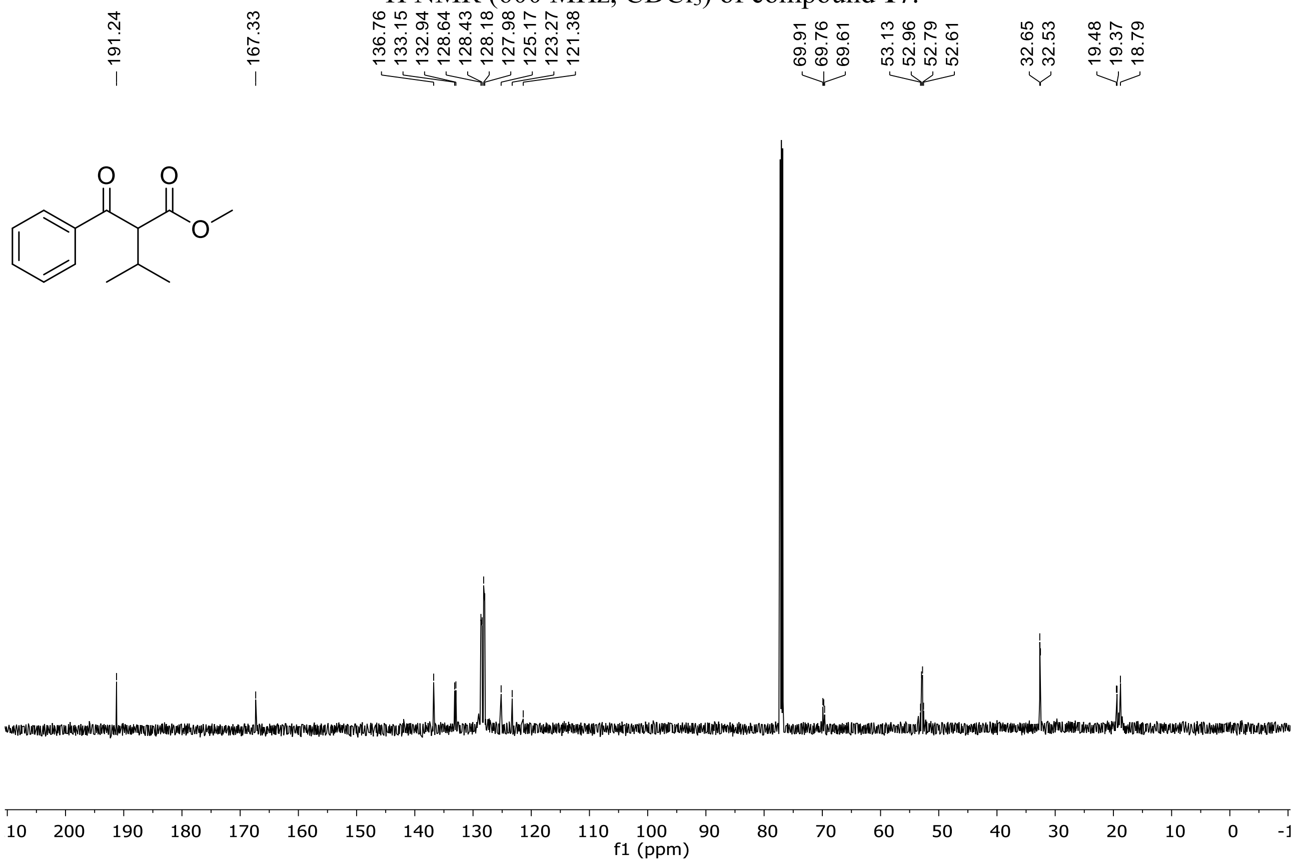

^{13}C{^{1}H} NMR (151 MHz, $CDCl_3$) of compound **17**.

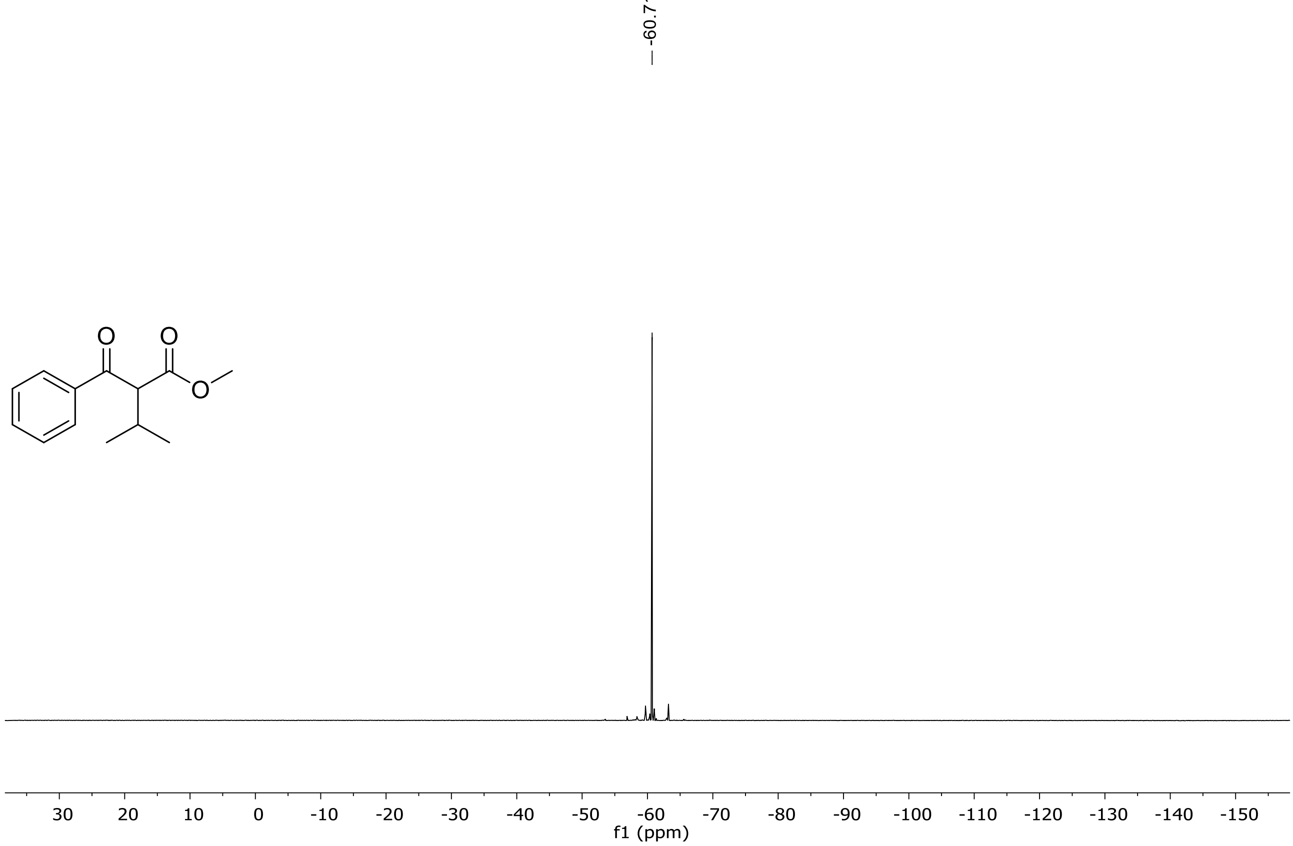

^{19}F NMR (282 MHz, $CDCl_3$) of compound **17**.

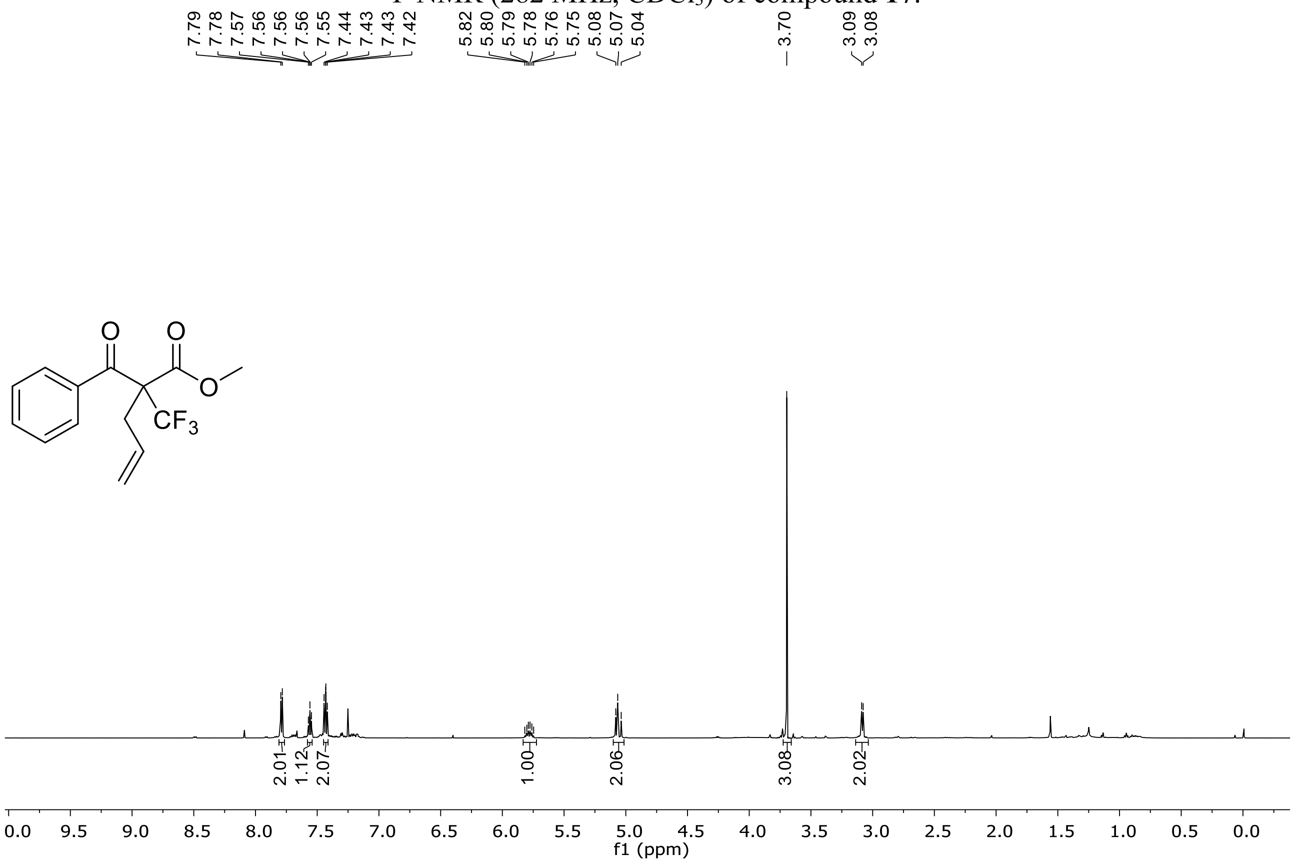

^{1}H NMR (600 MHz, $CDCl_3$) of compound **18**.

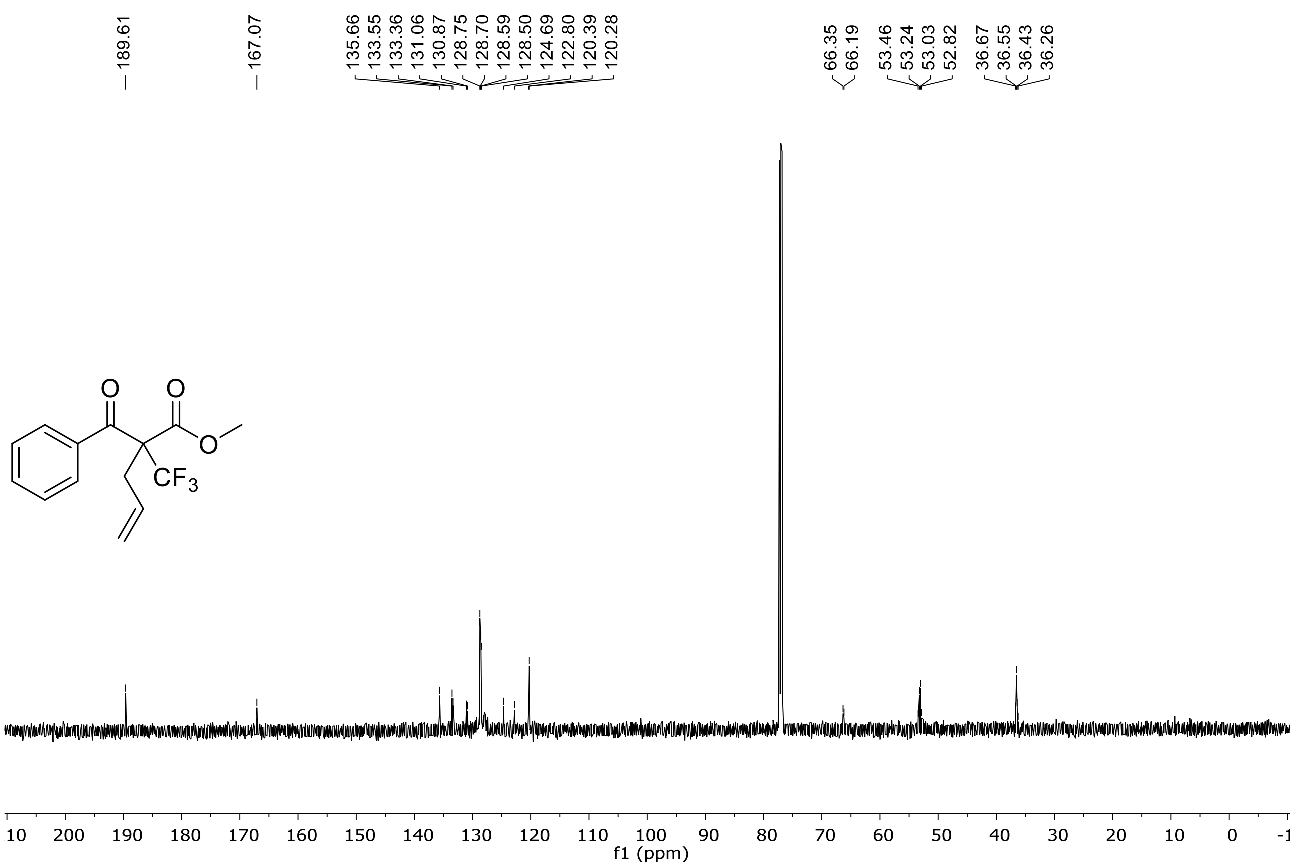

$^{13}C\{^{1}H\}$ NMR (151 MHz, $CDCl_3$) of compound **18**.

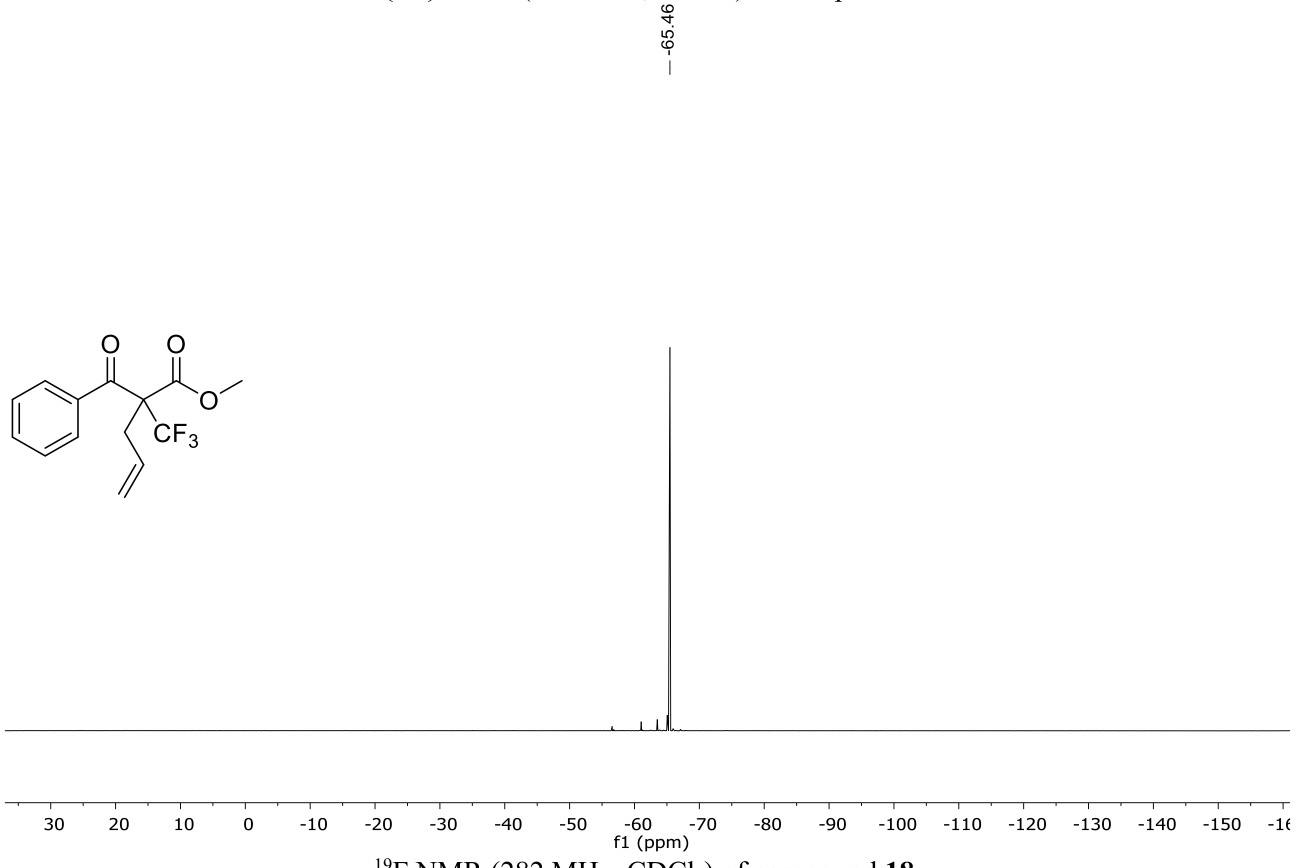

^{19}F NMR (282 MHz, $CDCl_3$) of compound **18**.

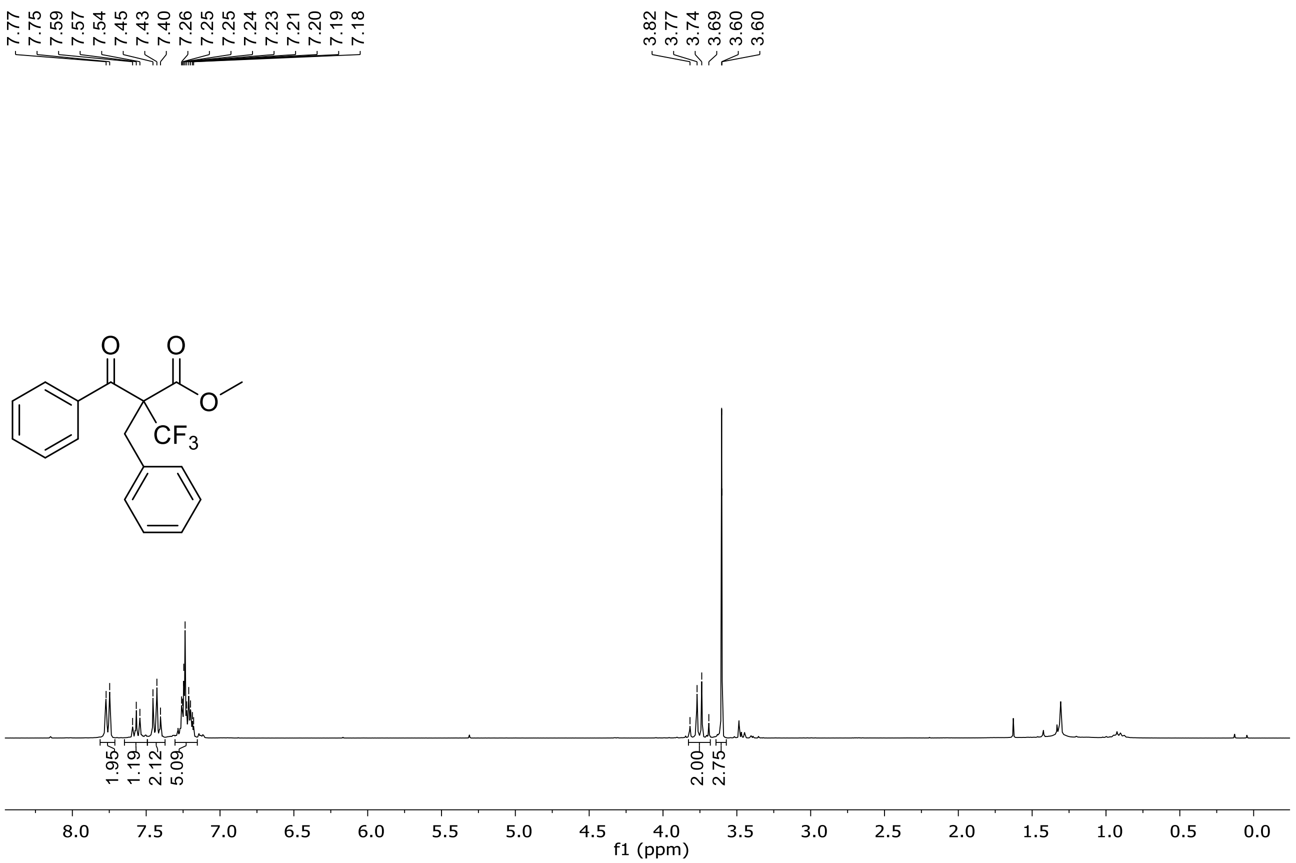

^{1}H NMR (300 MHz, $CDCl_3$) of compound **19**.

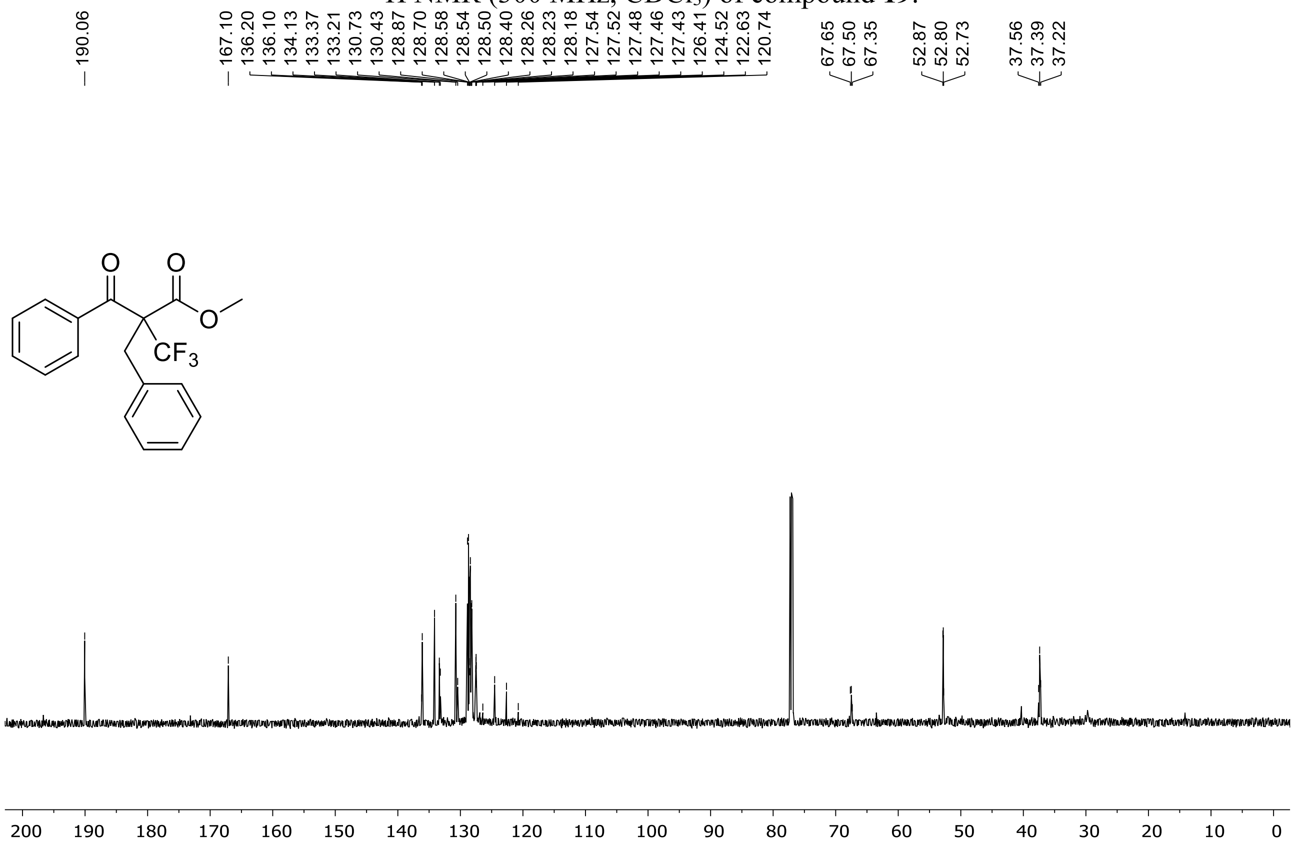

$^{13}C\{^{1}H\}$ NMR (151 MHz, $CDCl_3$) of compound **19**.

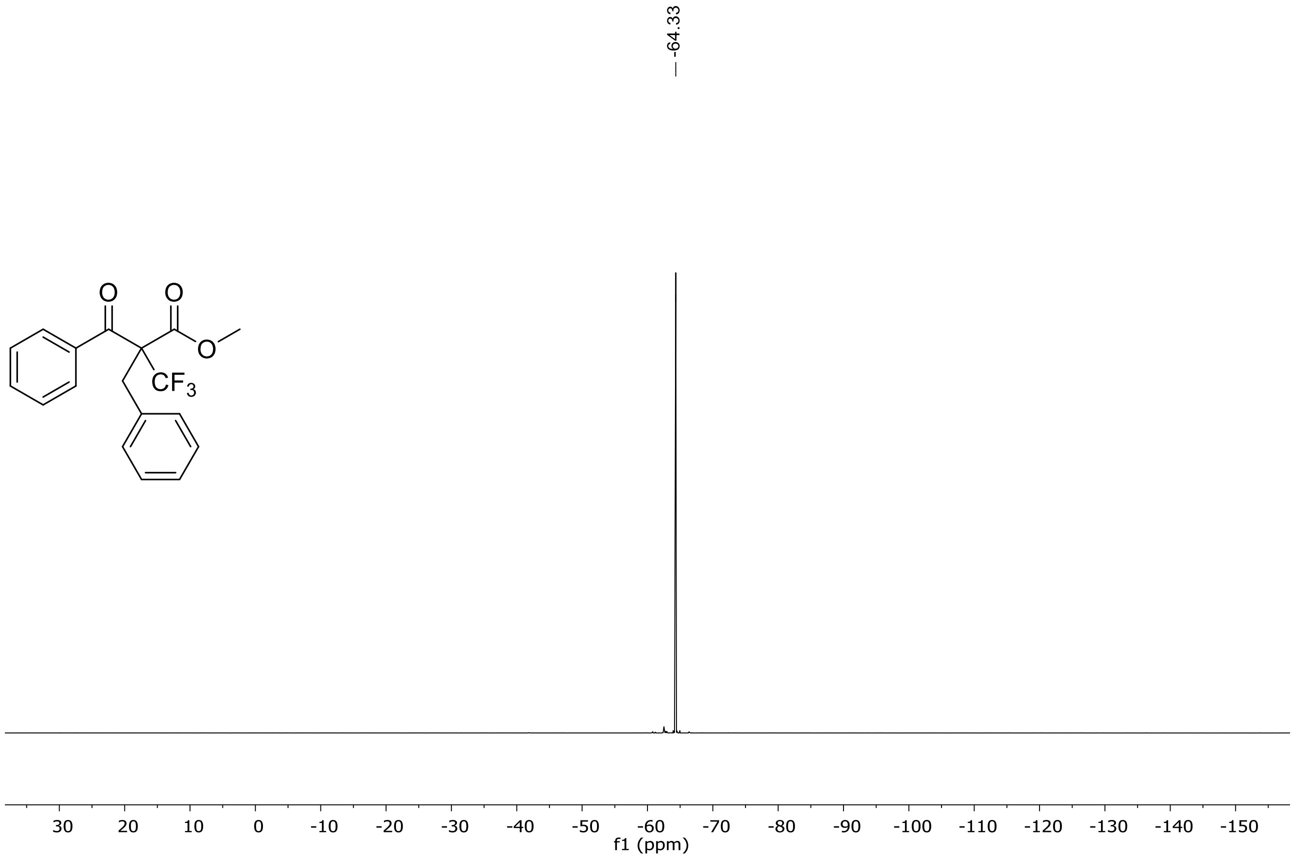

^{19}F NMR (282 MHz, CDCl$_3$) of compound **19**.

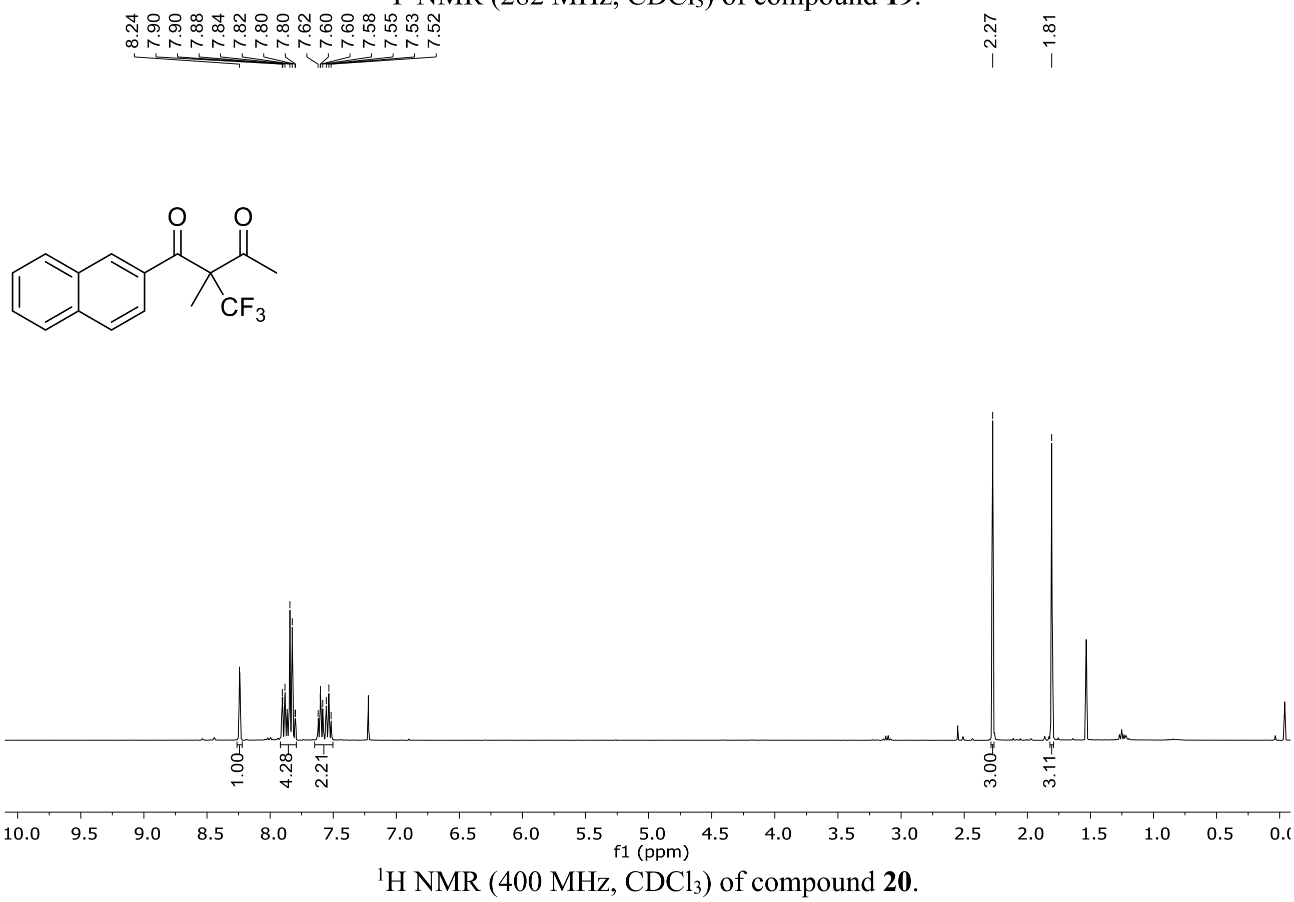

^{1}H NMR (400 MHz, CDCl$_3$) of compound **20**.

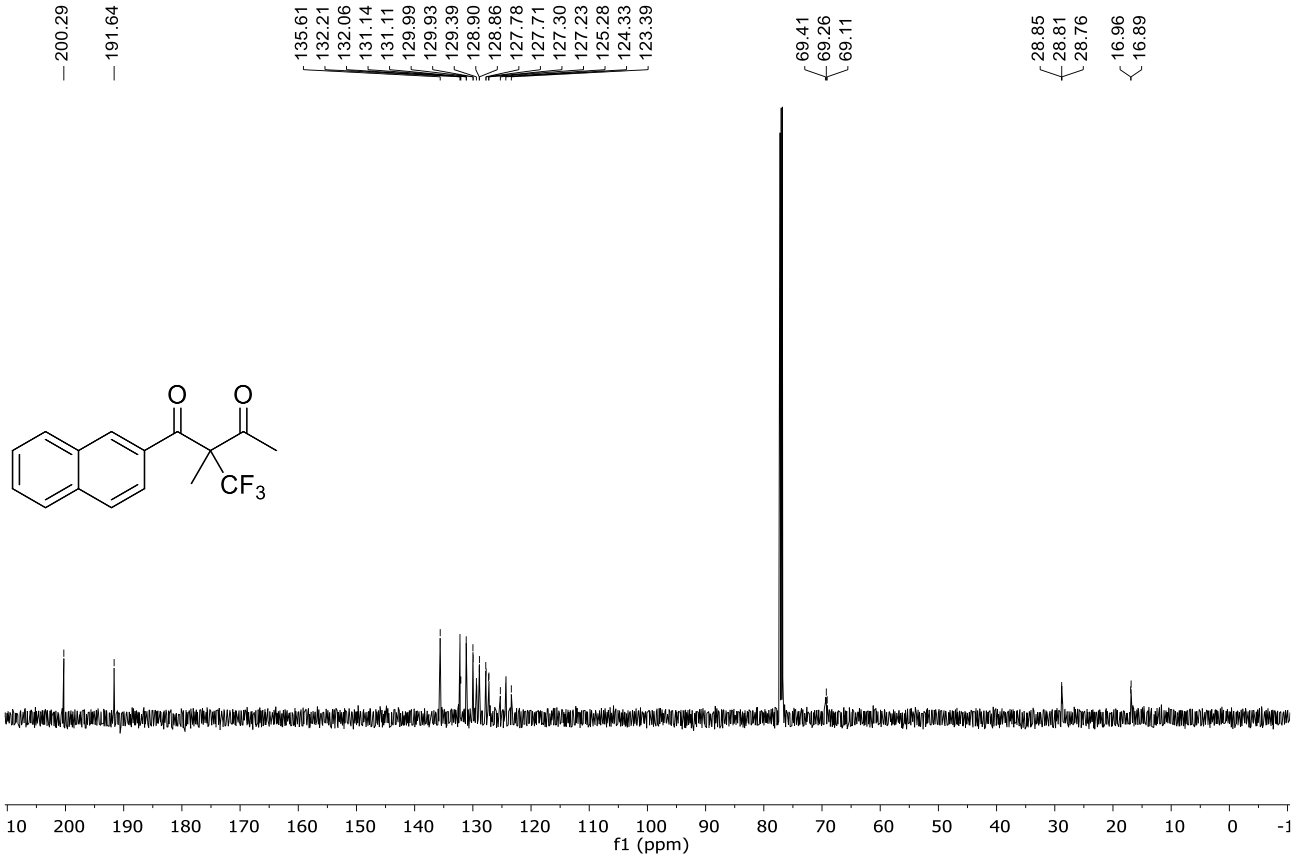

$^{13}C\{^1H\}$ NMR (151 MHz, $CDCl_3$) of compound **20**.

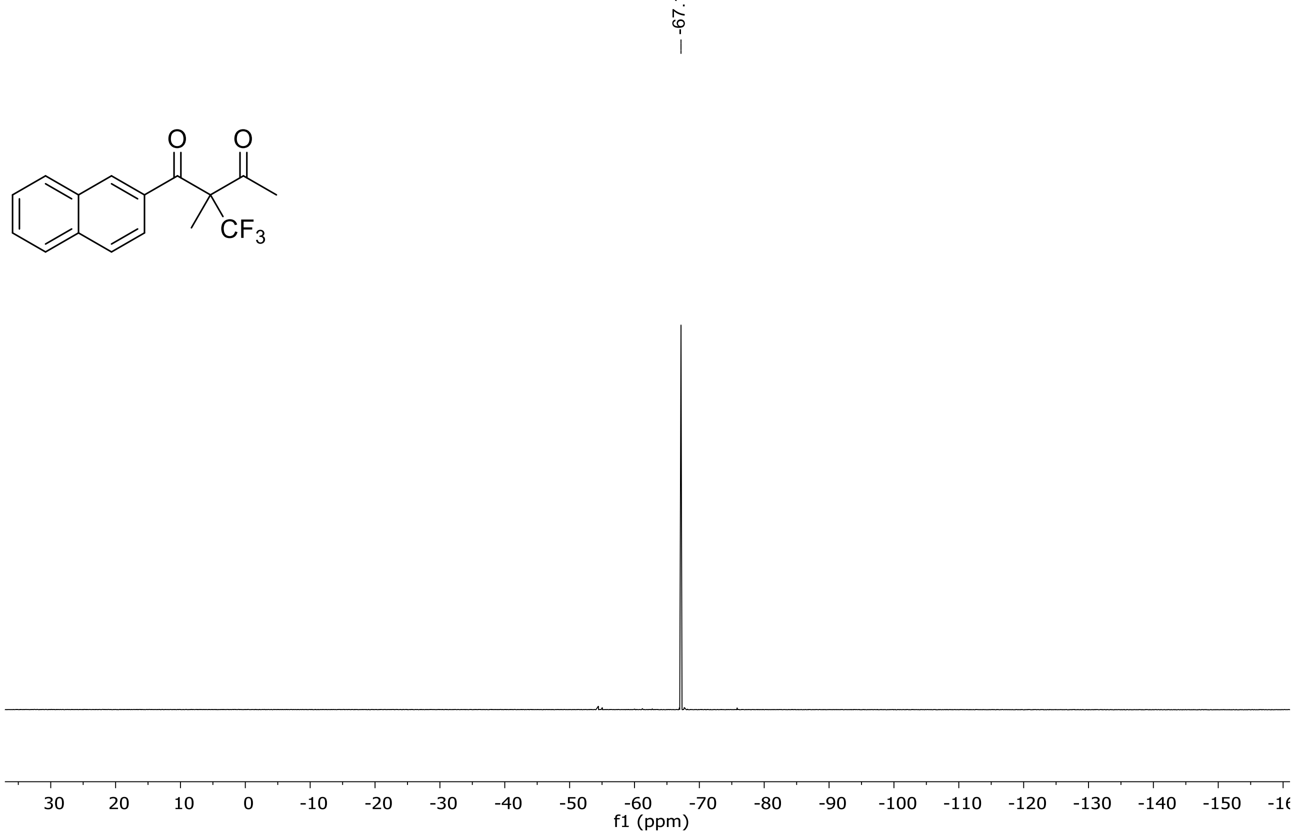

^{19}F NMR (282 MHz, $CDCl_3$) of compound **20**.

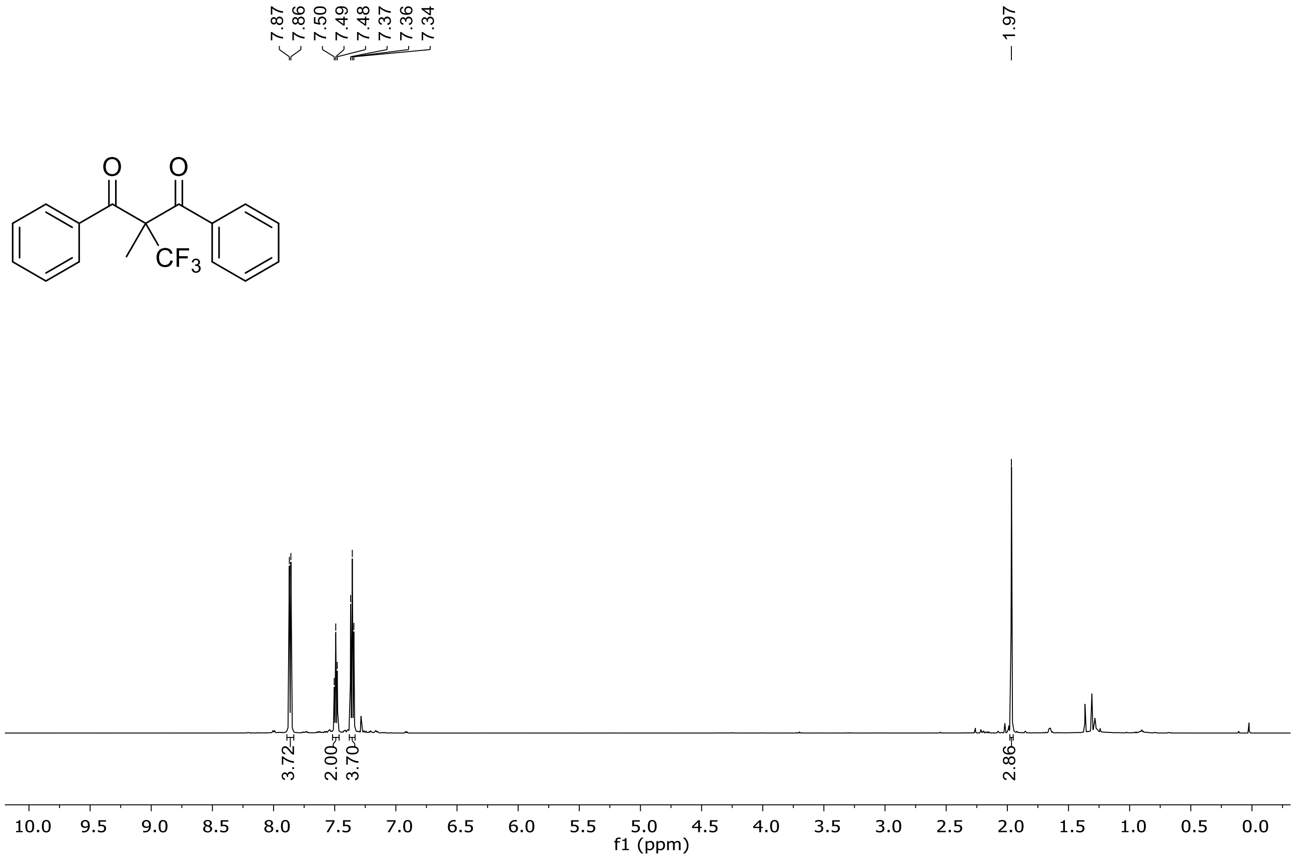

^{1}H NMR (600 MHz, $CDCl_3$) of compound **21**.

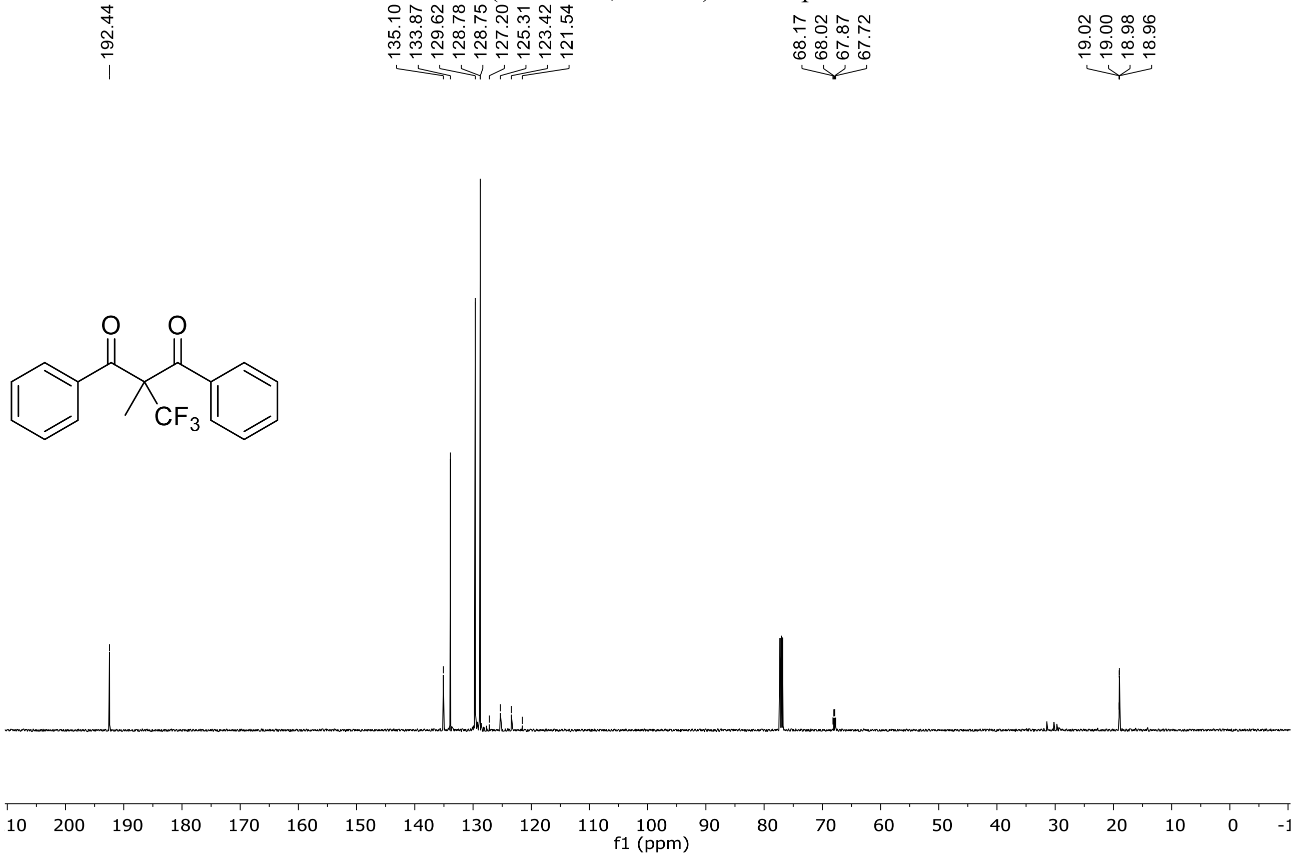

^{13}C{^{1}H} NMR (151 MHz, $CDCl_3$) of compound **21**.

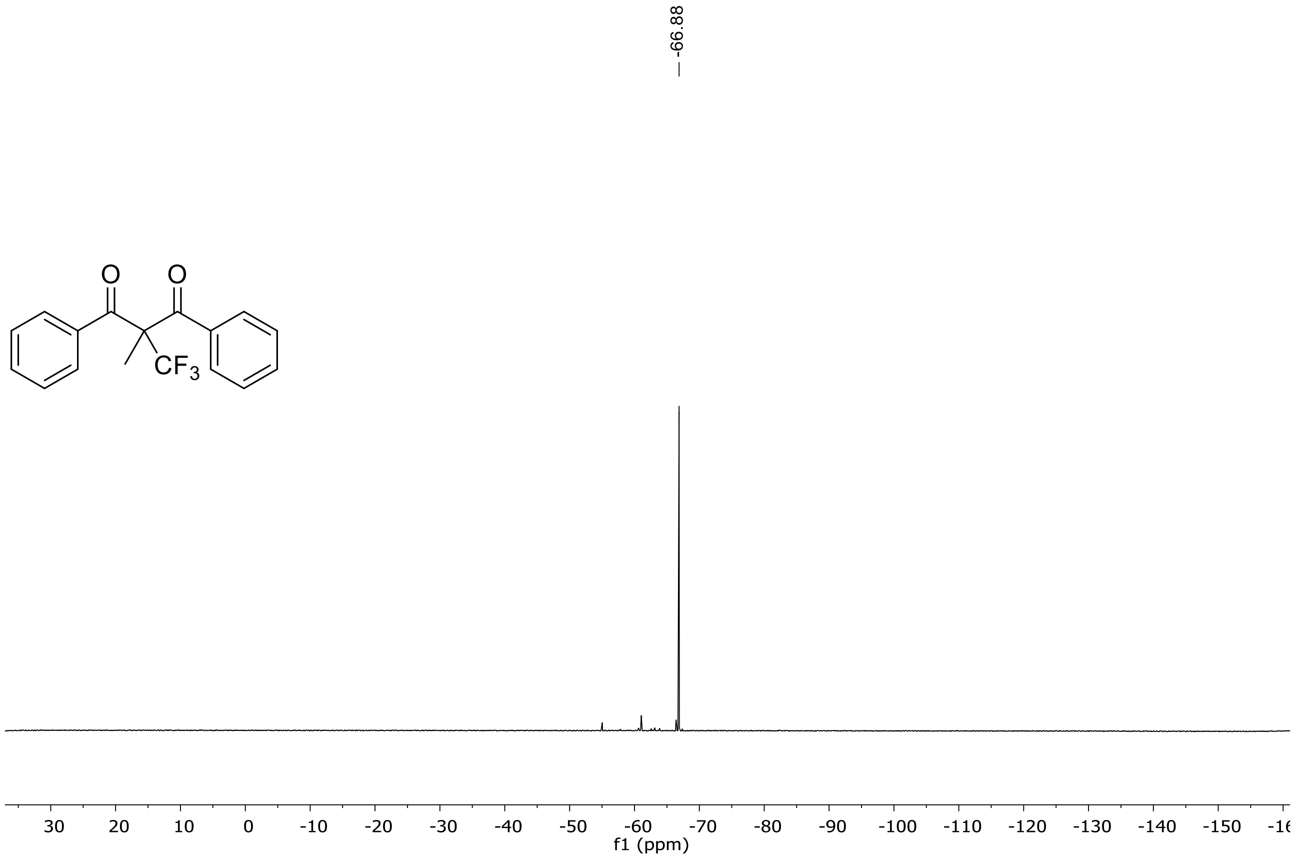

^{19}F NMR (377 MHz, CDCl3) of compound **21**

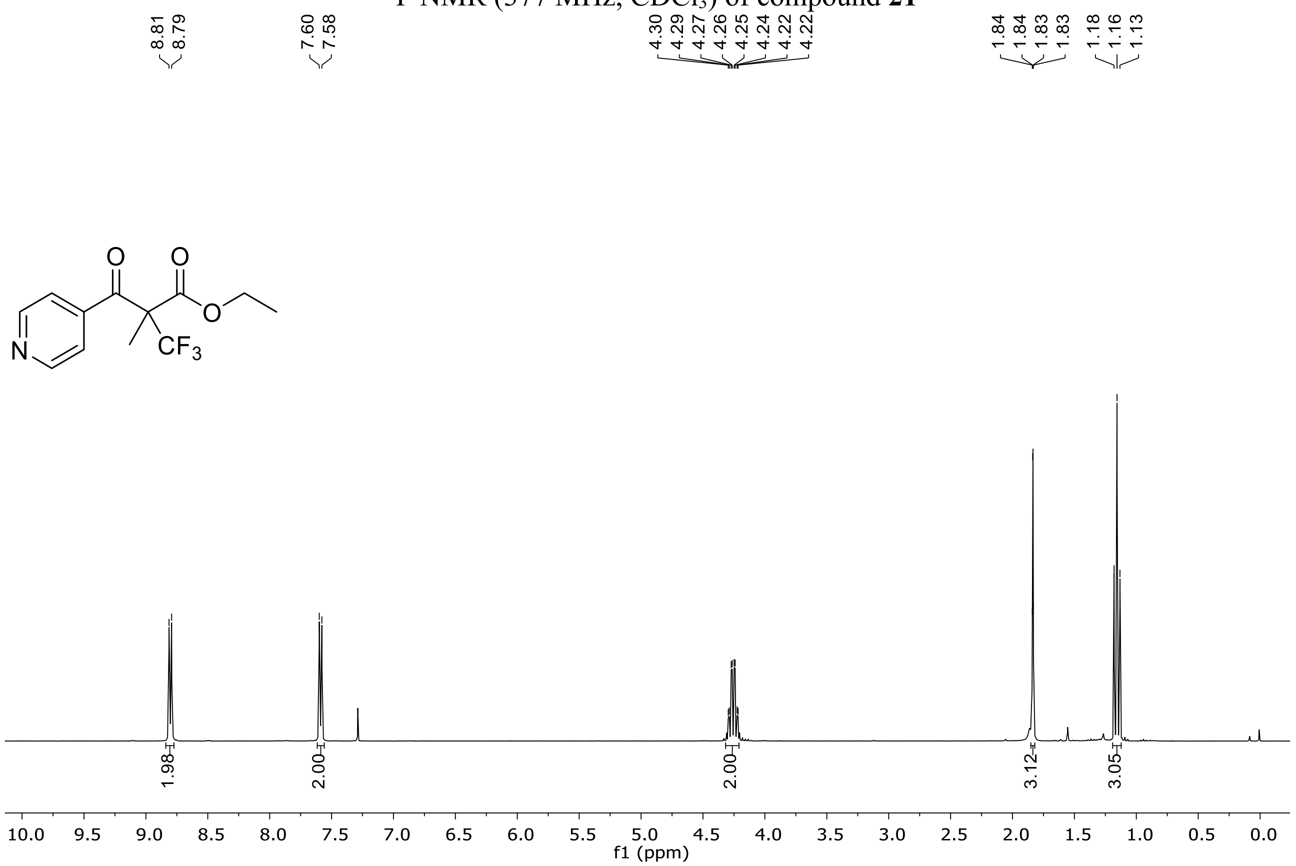

^{1}H NMR (300 MHz, CDCl3) of compound **22**

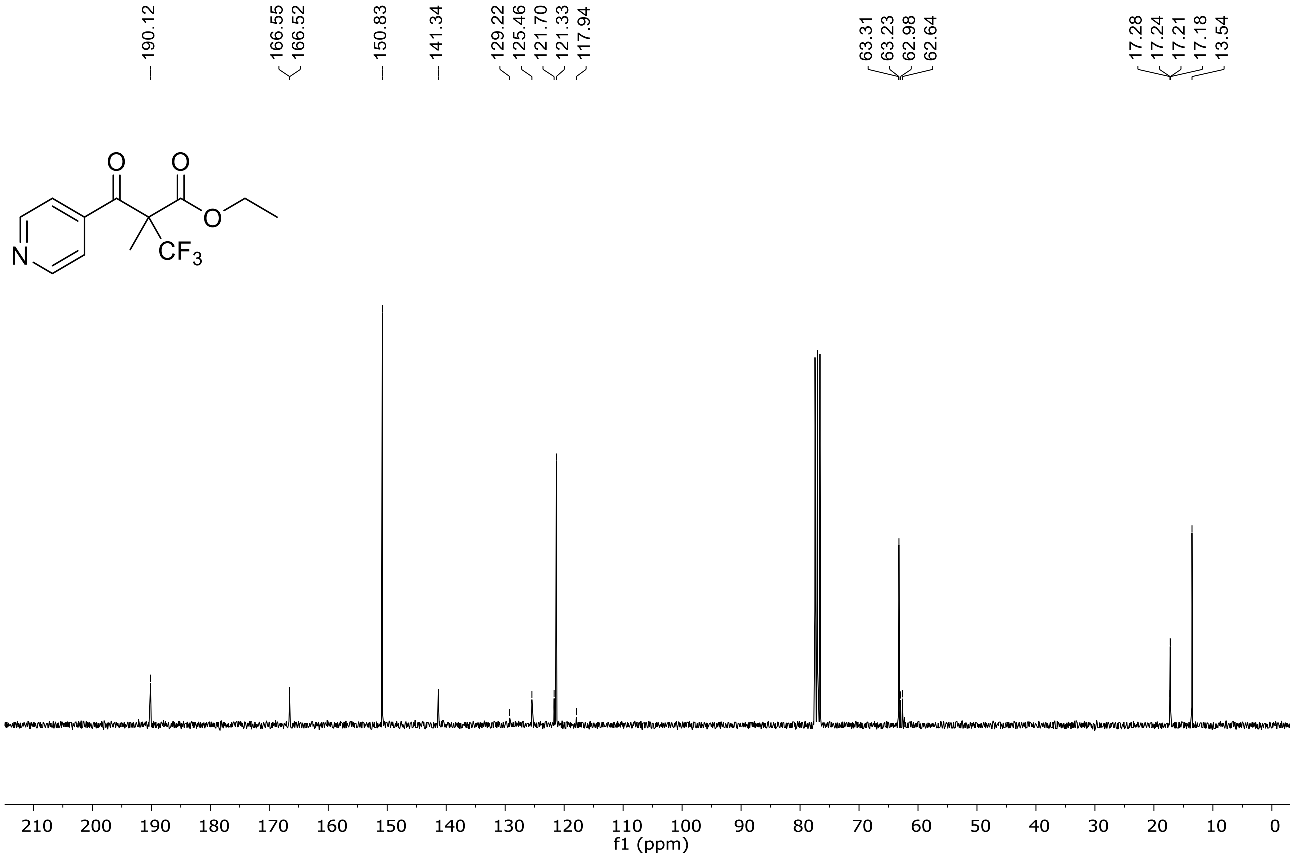

$^{13}C\{^{1}H\}$ NMR (75 MHz, $CDCl_3$) of compound **22**

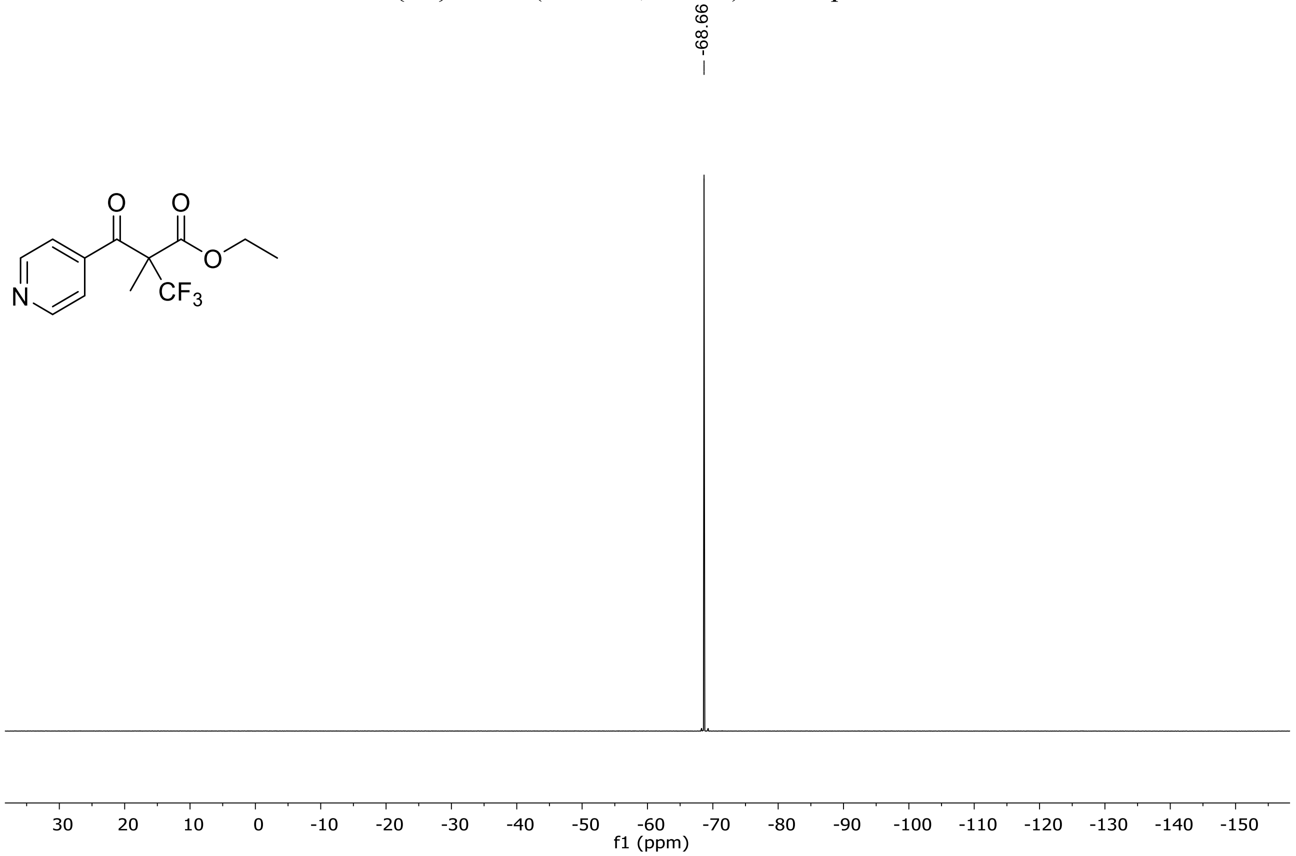

^{19}F NMR (377 MHz, $CDCl_3$) of compound **22**

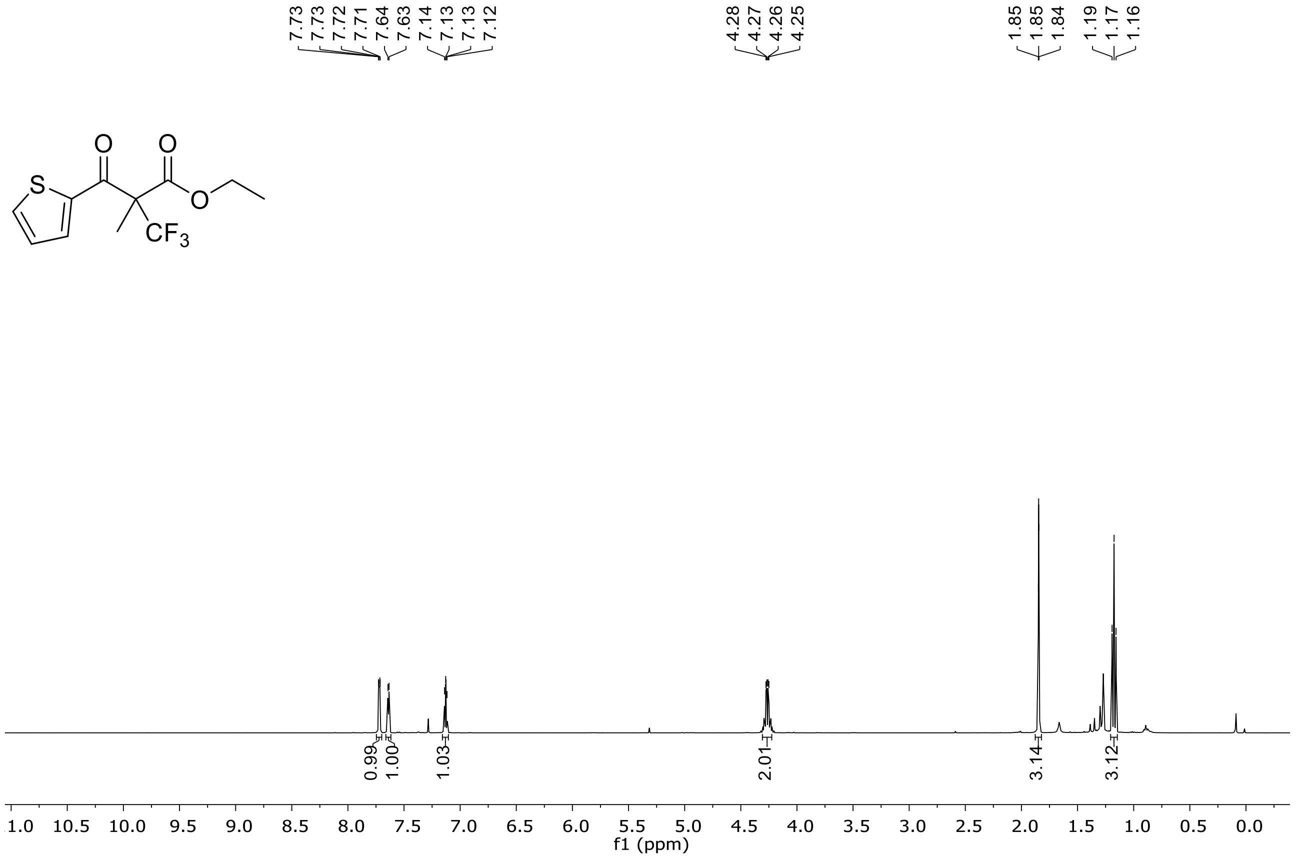

^{1}H NMR (400 MHz, $CDCl_3$) of compound **23**.

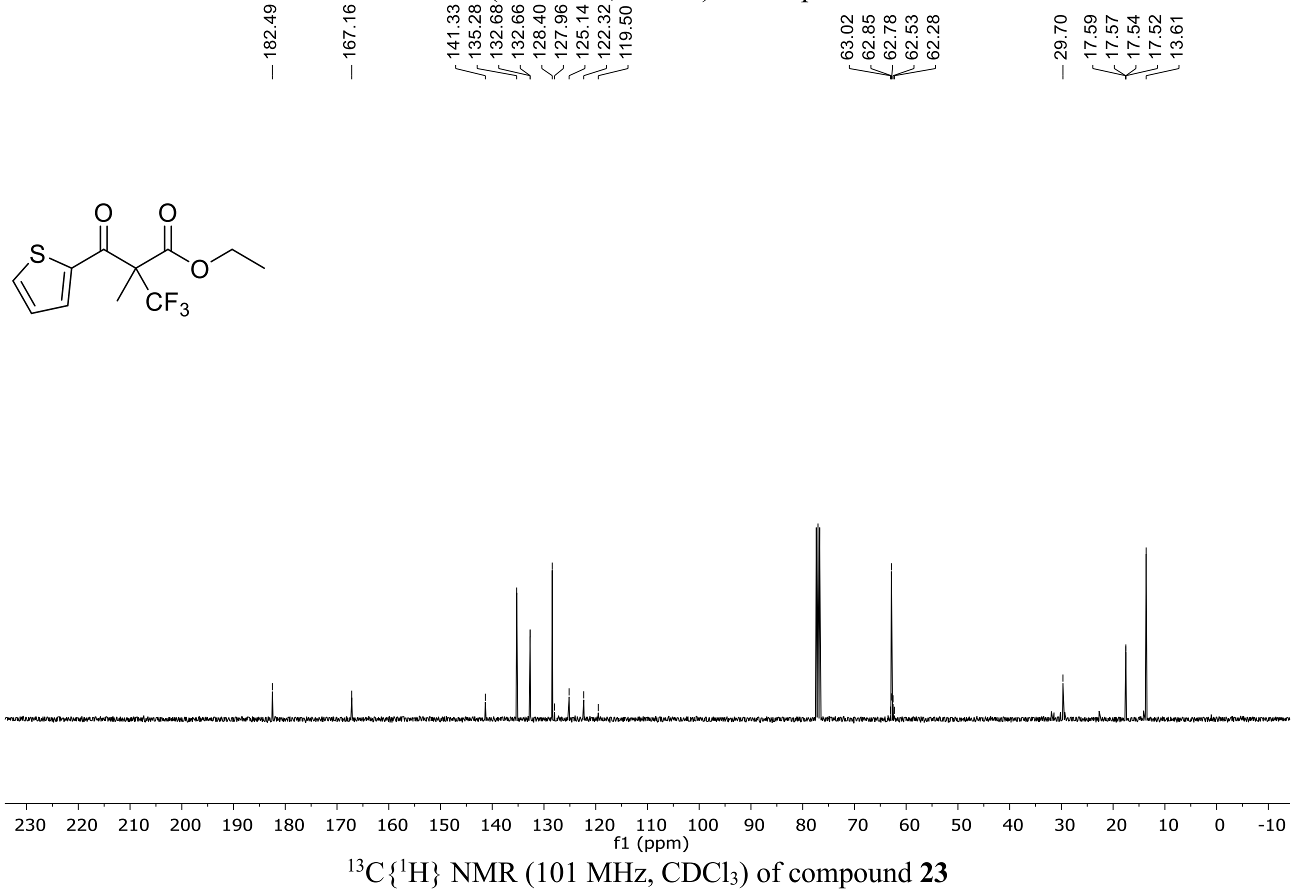

^{13}C{^{1}H} NMR (101 MHz, $CDCl_3$) of compound **23**

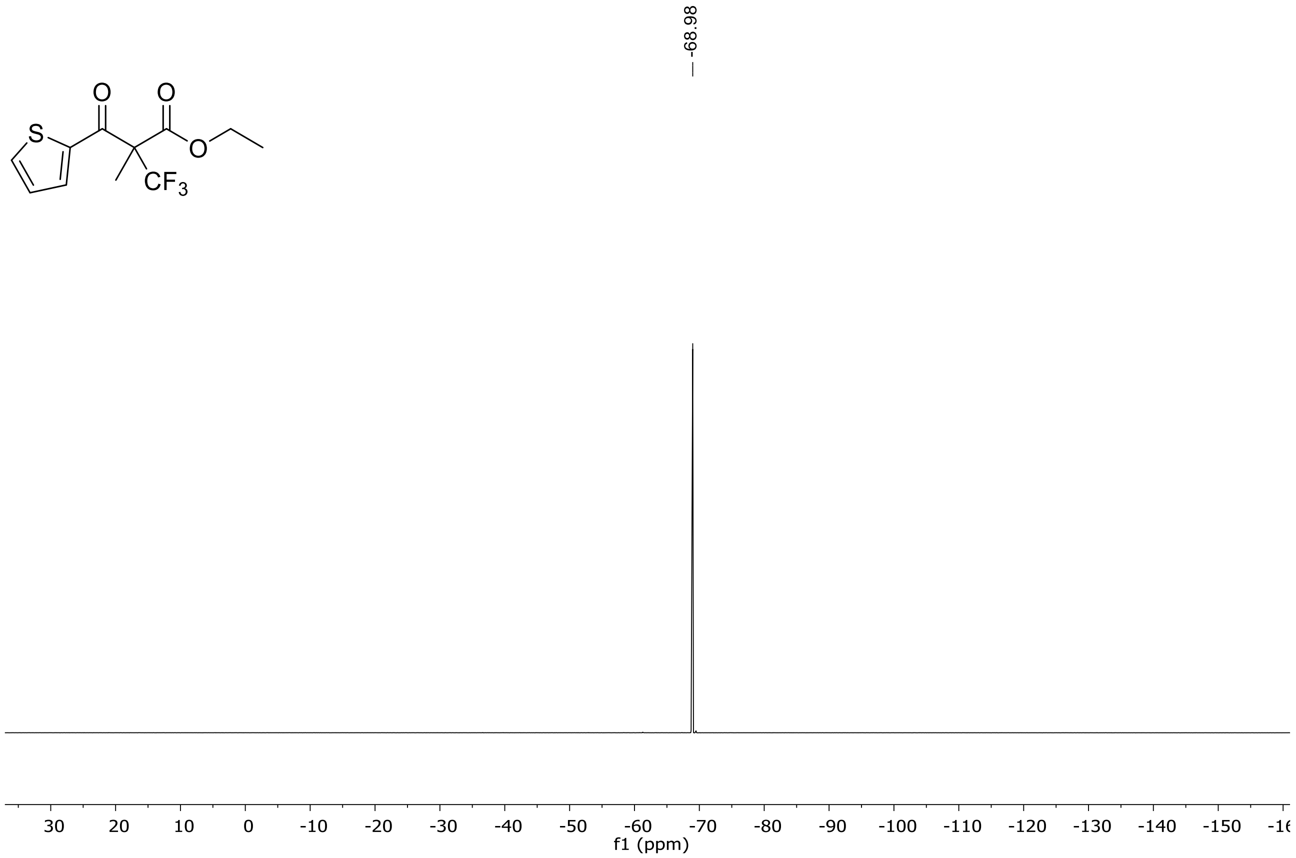

^{19}F NMR (377 MHz, CDCl$_3$) of compound **23**

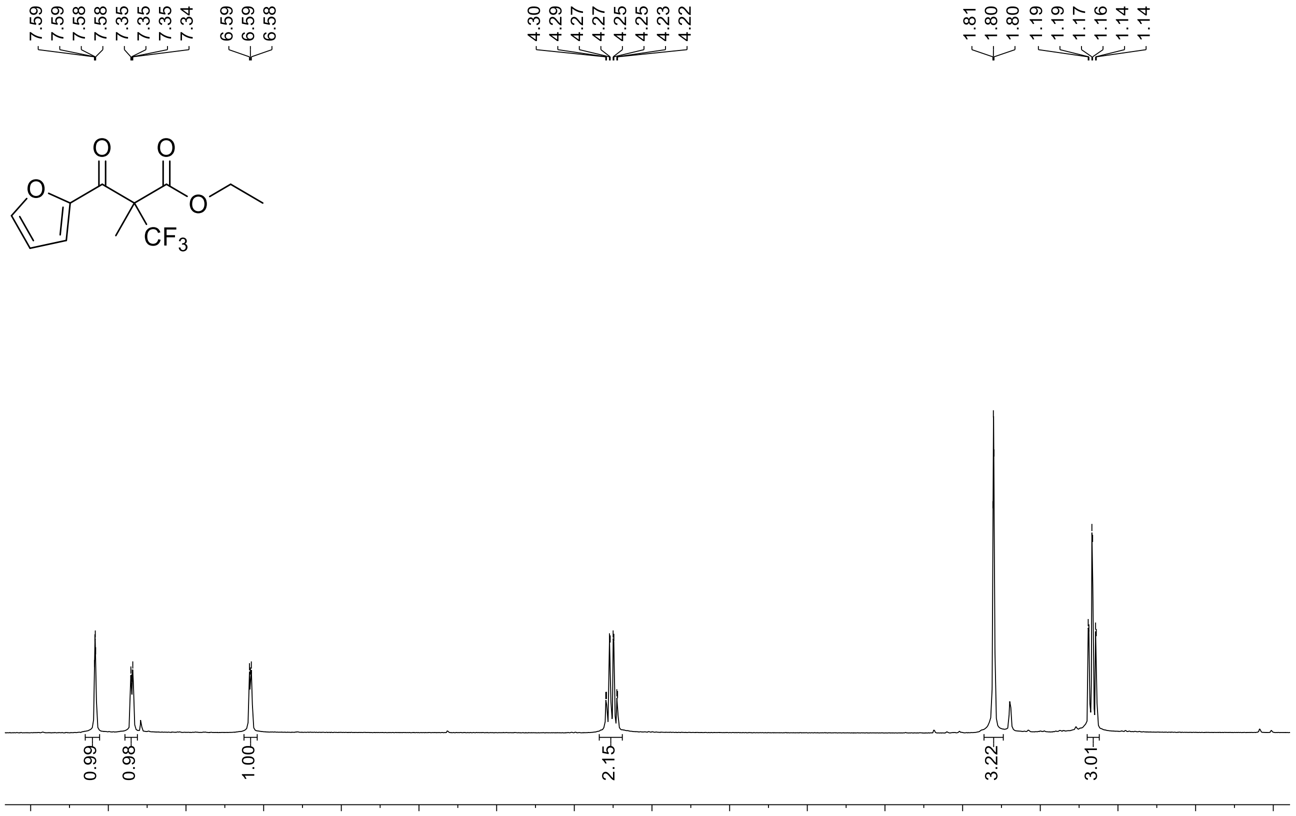

^{1}H NMR (300 MHz, CDCl$_3$) of compound **24**

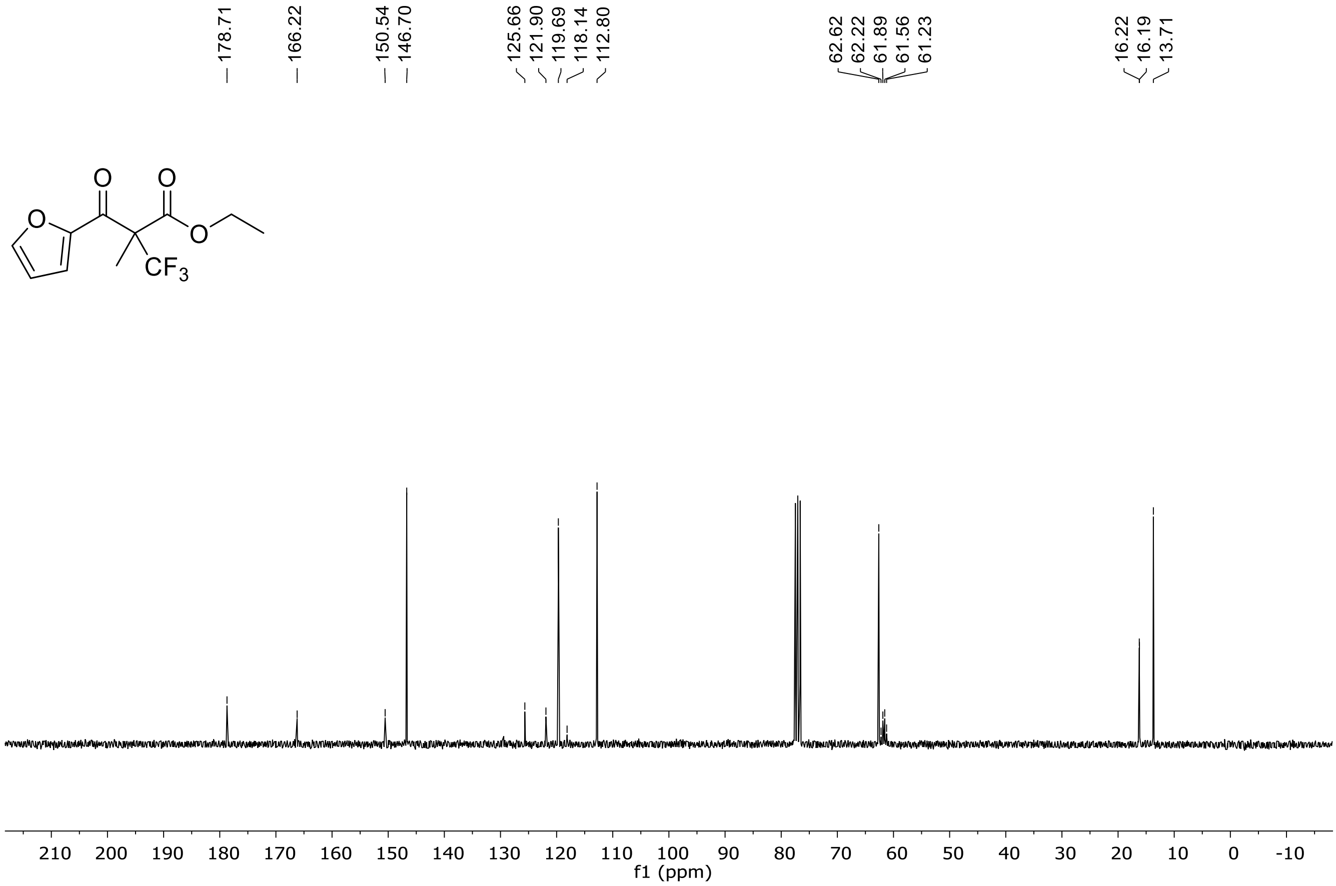

$^{13}C\{^{1}H\}$ NMR (75 MHz, $CDCl_3$) of compound **24**

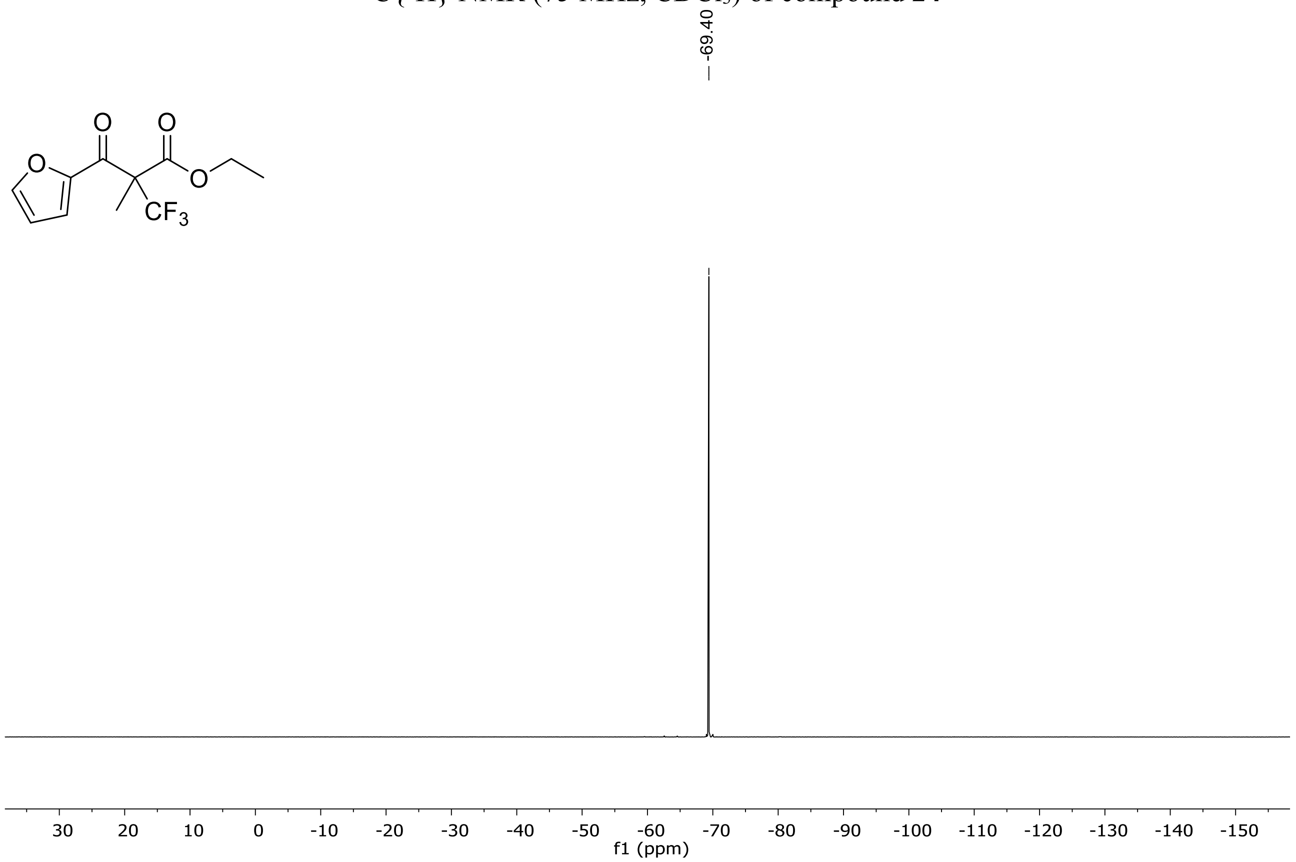

^{19}F NMR (282 MHz, $CDCl_3$) of compound **24**

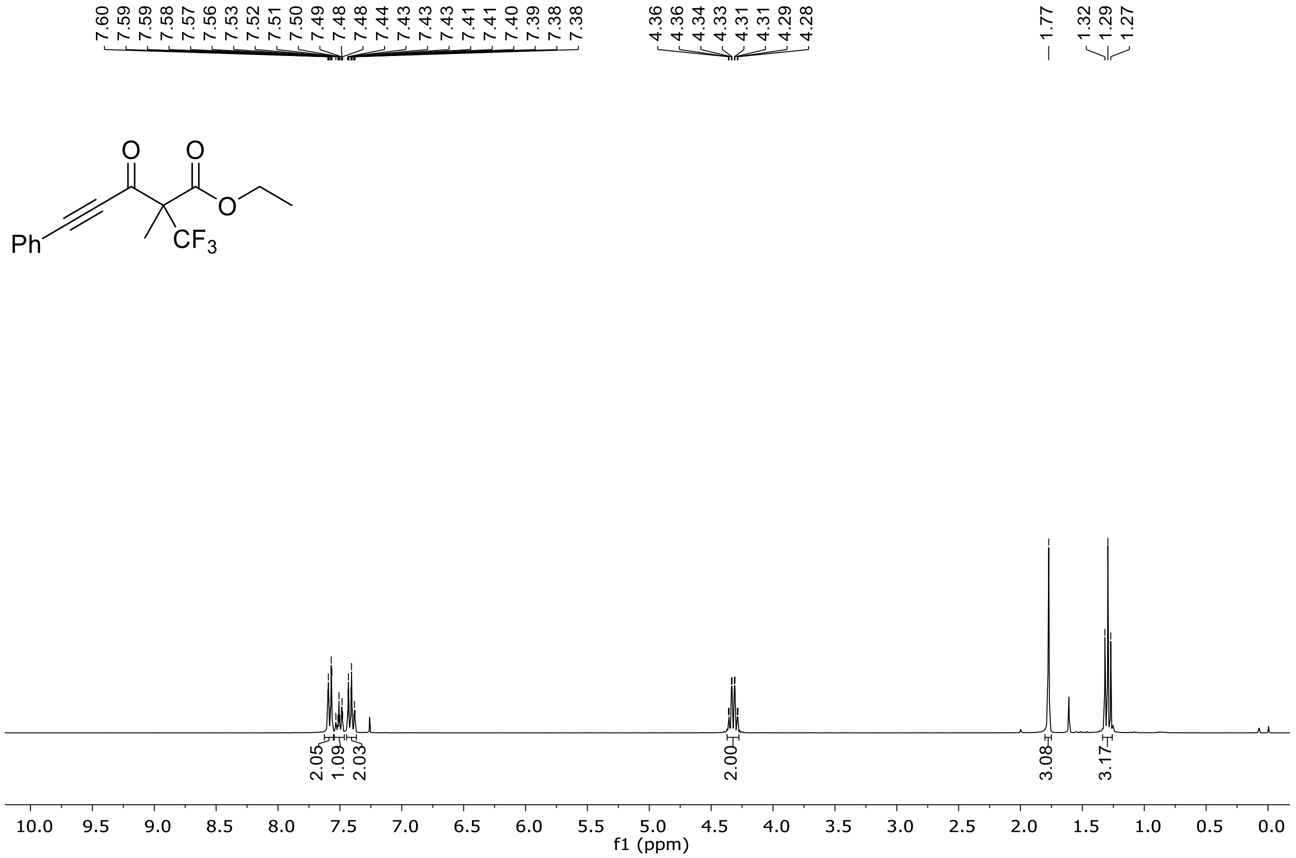

^{1}H NMR (300 MHz, $CDCl_3$) of compound **25**

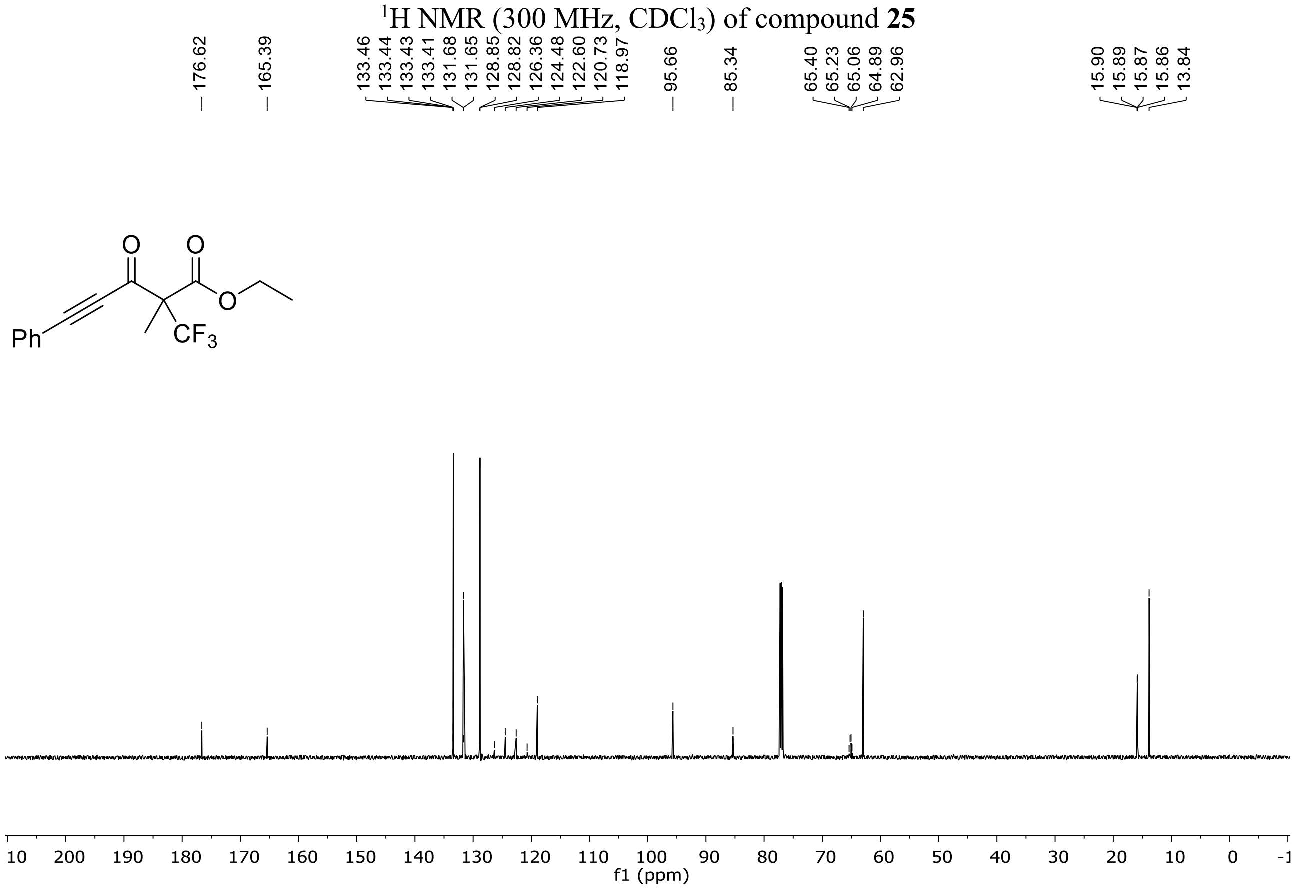

$^{13}C\{^1H\}$ NMR (151 MHz, $CDCl_3$) of compound **25**

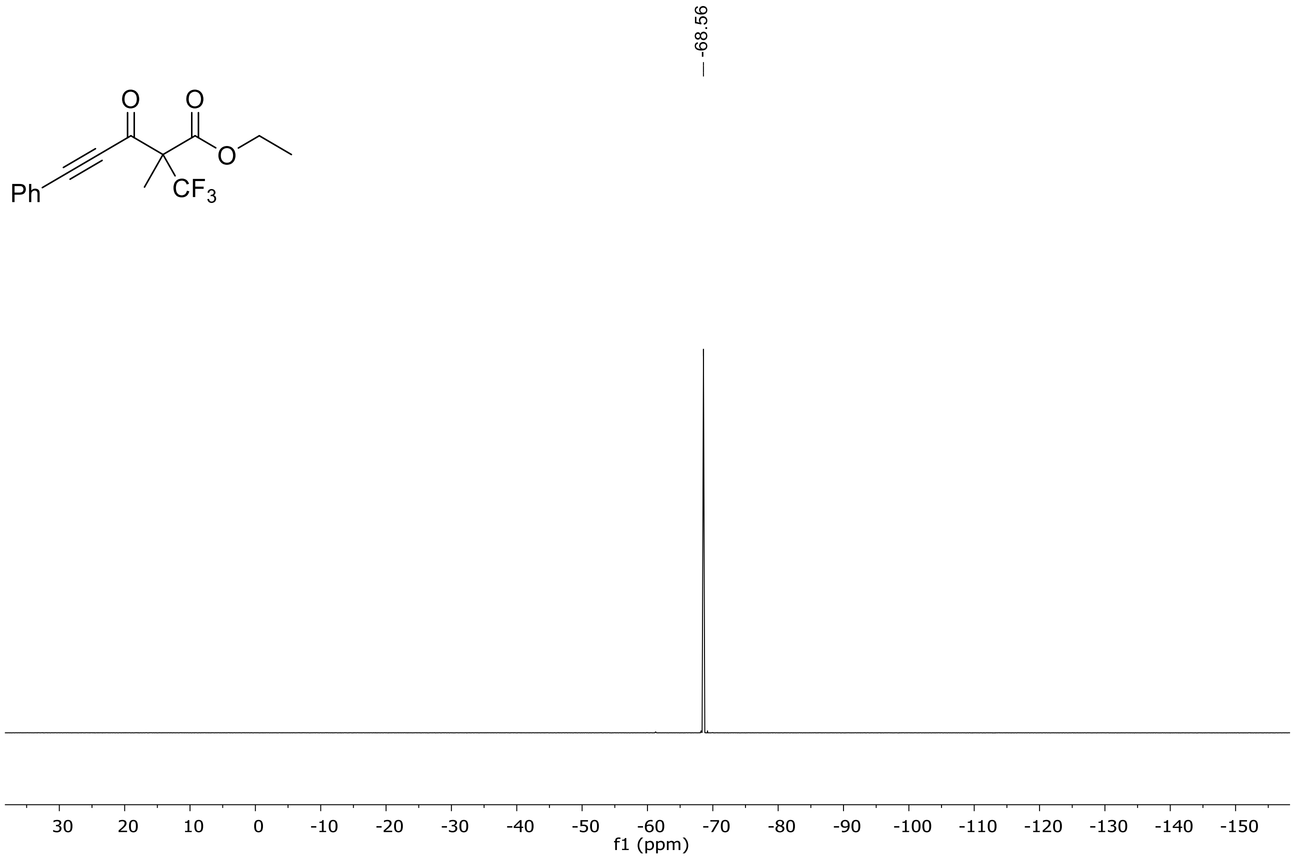

^{19}F NMR (282 MHz, $CDCl_3$) of compound **25**

6. References

1. Zhang, Z.; Liu, Y.; Gong, M:; Zhao, X.; Zhang, Y.; Wang, J. Palladium-Catalyzed Carbonylation/Acyl Migratory Insertion Sequence. *Angew. Chem. Int. Ed.* **2010**, *49*, 1139–1142.

2. Pericas, A.; Shafir, A.; Vallribera, A. Asymmetric Synthesis of L-Carbidopa Based on a Highly Enantioselective α-Amination. *Org. Lett.* **2013,** *15*, 1448–1451.

3. Granados, A.; del Olmo, A.; Peccati, F.; Billard, T.; Sodupe, M.; Vallribera, A. Fluorous L-Carbidopa Precursors: Highly Enantioselective Synthesis and Computational Prediction of Bioactivity *J. Org. Chem.* **2018**, *83*, 303−313.

4. Guo, S.; Wang, Q.; Jiang, Y.; Yu, J. *tert*-Butyl Peroxybenzoate-Promoted α-Methylation of 1,3-Dicarbonyl Compounds. *J. Org. Chem*. **2014,** 79, 11285-11289.

5. Taber, S.; You, K.; Song, Y. A Simple Preparation of alpha-Diazo Esters. *J. Org. Chem.* **1995** *60*, 1093-1094.

6. Wahl, B.; Bonin, H.; Mortreux, A.; Giboulot, S.; Liron, F.; Poli, G.; Sauthier, M. A General and Efficient Method for the Alkoxycarbonylation alfa-Chloro Ketones. *Adv. Synth. Catal.* **2012**, *354*, 3105 – 3114.

7. Chowdhury, S.; Koley, S.; Chanda, T.; Singh, M. In/I_2 mediated functional group transformation: a direct approach toward the selective conversion of dithioester to ester, *Tetrahedron Lett*. **2015**, *56*, 5553-555

8. Pericas, A.; Jiménez, R.; Granados, A.; Shafir, A.; Vallribera, A.; Roglans, A.; Molins, E. Lanthanides–pybox: An Excellent Combination for Highly Enantioselective Electrophilic α-Amination of Acyclic β-Keto Esters. Isolation of Ternary Pybox/Ln/β-Keto Ester Complexes. *ChemistrySelect* **2016**, *1*, 4305 – 4312.

9. Pericas, A.; Shafir, A.; Vallribera, A. Zinc(II) oxide: an Efficient Catalyst for Selective Transesterification of β- ketoesters *Tetrahedron*, **2008**, *64*, 9258-9263.

10. Poorsadeghi, S.; Endo, K.; Arimitsu, S. Enantioselective Fluorination of α-Substituted β-Diketones Using β,β-Diaryl Serines. *Org. Lett.* **2022**, *24*, 420-424.

11. Gøgsig, T.; Taaning, R.; Lindhardt, A.; Skrydstrup, T. Palladium-Catalyzed Carbonylative α-Arylation for Accessing 1,3-Diketones. *Angew. Chem. Int. Ed.* **2012**, *51*, 798-801.

12. Inagaki, S.; Saito, K.; Suto, S.; Aihara, H.; Sugawara, A.; Tamura, S.; Kawano, T. Synthesis of 5-Aryl-3(2H)-furanones Using Intramolecular Cyclization of Sulfonium Salts. *J. Org. Chem*. **2018**, *83*, 13834-13846.

13. Jia, H.; Häring, A.; Berger, F.; Zhang, L.; Ritter, T. Trifluoromethyl Thianthrenium Triflate: A Readily Available Trifluoromethylating Reagent with Formal CF_3^+, CF_3•, and CF_3^- Reactivity. *J. Am. Chem. Soc.* **2021** *143*, 7623-7628.

14. Matsnev, A.; Noritake, S.; Nomura, Y.; Tokunaga, E.; Nakamura, S.; Shibata, N. Efficient access to extended Yagupolskii-Umemoto-type reagents: triflic acid catalyzed intramolecular cyclization of ortho-ethynylaryltrifluoromethylsulfanes. *Angew. Chem., Int. Ed.* **2010**, *49*, 572–576.

15. Granados, A.; Rivilla, I.; Cossío, F.; Vallribera, A. Lanthanum-Catalyzed Enantioselective Trifluoromethylation by Using an Electrophilic Hypervalent Iodine Reagent. *Chem. Eur. J.* **2019**, *25*, 8214–8218.

16. Katritzky, A. R.; Wang, Z.; Wang, M.; Wilkerson, C. R.; Hall, C. D.; Akhmedov, N. G. . Preparation of β-Keto Esters and β-Diketones by C-Acylation/Deacetylation of Acetoacetic Esters and Acetonyl Ketones with 1-Acylbenzotriazoles. *J. Org. Chem.* **2004**, *69,* 6617–6622.

17. Kumaraswamy, G.; Narayanarao, V.; Shanigaram, P.; Balakishan, G. Diastereoselective synthesis of an advanced intermediate of the crocacin family using asymmetric transfer hydrogenation-DKR and Marshall allenylation as key reactions. *Tetrahedron.* **2015**, *71,* 8960–8964.
